# Baseline ctDNA gene alterations as a biomarker of survival after panitumumab and chemotherapy in metastatic colorectal cancer

**DOI:** 10.1038/s41591-023-02791-w

**Published:** 2024-02-12

**Authors:** Kohei Shitara, Kei Muro, Jun Watanabe, Kentaro Yamazaki, Hisatsugu Ohori, Manabu Shiozawa, Atsuo Takashima, Mitsuru Yokota, Akitaka Makiyama, Naoya Akazawa, Hitoshi Ojima, Yasuhiro Yuasa, Keisuke Miwa, Hirofumi Yasui, Eiji Oki, Takeo Sato, Takeshi Naitoh, Yoshito Komatsu, Takeshi Kato, Ikuo Mori, Kazunori Yamanaka, Masamitsu Hihara, Junpei Soeda, Toshihiro Misumi, Kouji Yamamoto, Riu Yamashita, Kiwamu Akagi, Atsushi Ochiai, Hiroyuki Uetake, Katsuya Tsuchihara, Takayuki Yoshino

**Affiliations:** 1 Department of Gastroenterology and Gastrointestinal Oncology, National Cancer Center Hospital East, Kashiwa, Japan; 2 Department of Immunology, Nagoya University Graduate School of Medicine, Aichi, Japan; 3 Department of Clinical Oncology, Aichi Cancer Center Hospital, Nagoya, Japan; 4 Department of Surgery, Gastroenterological Center, Yokohama City University Medical Center, Yokohama, Japan; 5 Division of Gastrointestinal Oncology, Shizuoka Cancer Center, Shizuoka, Japan; 6 Division of Medical Oncology, Japanese Red Cross Ishinomaki Hospital, Miyagi, Japan; 7 Department of Gastrointestinal Surgery, Kanagawa Cancer Center, Kanagawa, Japan; 8 Department of Gastrointestinal Medical Oncology, National Cancer Center Hospital, Tokyo, Japan; 9 Department of General Surgery, Kurashiki Central Hospital, Okayama, Japan; 10 Department of Hematology/Oncology, Japan Community Healthcare Organization, Fukuoka, Japan; 11 Cancer Center, Gifu University Hospital, Gifu, Japan; 12 Division of Animal Medical Science, Center for One Medicine Innovative Translational Research, Gifu, Japan; 13 Department of Gastroenterological Surgery, Sendai City Medical Center, Sendai Open Hospital, Miyagi, Japan; 14 Department of Gastroenterological Surgery, Gunma Prefectural Cancer Center, Gunma, Japan; 15 Department of Gastroenterological Surgery, Japanese Red Cross Tokushima Hospital, Tokushima, Japan; 16 Multidisciplinary Treatment Cancer Center, Kurume University Hospital, Kurume, Japan; 17 Department of Surgery and Science, Graduate School of Medical Sciences, Kyushu University, Fukuoka, Japan; 18 Research and Development Center for Medical Education, Department of Clinical Skills Education, Kitasato University School of Medicine, Sagamihara, Japan; 19 Department of Lower Gastrointestinal Surgery, Kitasato University School of Medicine, Sagamihara, Japan; 20 Division of Cancer Chemotherapy, Hokkaido University Hospital Cancer Center, Sapporo, Japan; 21 Department of Surgery, National Hospital Organization Osaka National Hospital, Osaka, Japan; 22 Japan Medical Affairs, Japan Oncology Business Unit, Takeda Pharmaceutical Company Ltd, Tokyo, Japan; 23 Pharmaceutical Research Division, Takeda Pharmaceutical Company Ltd, Kanagawa, Japan; 24 Department of Biostatistics, Yokohama City University School of Medicine, Yokohama, Japan; 25 Division of Translational Informatics, Exploratory Oncology Research & Clinical Trial Center, National Cancer Center, Chiba, Japan; 26 Division of Molecular Diagnosis and Cancer Prevention, Saitama Cancer Center, Saitama, Japan; 27 Research Institute for Biomedical Sciences, Tokyo University of Science, Tokyo, Japan; 28 National Hospital Organization, Disaster Medical Center, Tokyo, Japan

**Keywords:** Prognostic markers, Cancer genetics

## Abstract

Certain genetic alterations and right-sided primary tumor location are associated with resistance to anti-epidermal growth factor (EGFR) treatment in metastatic colorectal cancer (mCRC). The phase 3 PARADIGM trial (*n* = 802) demonstrated longer overall survival with first-line anti-EGFR (panitumumab) versus antivascular endothelial growth factor (bevacizumab) plus modified FOLFOX6 in patients with *RAS* wild-type mCRC with left-sided primary tumors. This prespecified exploratory biomarker analysis of PARADIGM (*n* = 733) evaluated the association between circulating tumor DNA (ctDNA) gene alterations and efficacy outcomes, focusing on a broad panel of gene alterations associated with resistance to EGFR inhibition, including *KRAS*, *NRAS,* *PTEN* and extracellular domain *EGFR* mutations, *HER2* and *MET* amplifications, and *ALK*, *RET* and *NTRK1* fusions. Overall survival was prolonged with panitumumab plus modified FOLFOX6 versus bevacizumab plus modified FOLFOX6 in patients with ctDNA that lacked gene alterations in the panel (that is, negative hyperselected; median in the overall population: 40.7 versus 34.4 months; hazard ratio, 0.76; 95% confidence interval, 0.62–0.92) but was similar or inferior with panitumumab in patients with ctDNA that contained any gene alteration in the panel (19.2 versus 22.2 months; hazard ratio, 1.13; 95% confidence interval, 0.83–1.53), regardless of tumor sidedness. Negative hyperselection using ctDNA may guide optimal treatment selection in patients with mCRC. ClinicalTrials.gov registrations: NCT02394834 and NCT02394795.

## Abstract (web summary)

In an exploratory preplanned biomarker analysis of the phase 3 PARADIGM trial, a lack of resistance gene alterations in baseline circulating tumor DNA (negative hyperselection) was associated with prolonged overall survival after first-line panitumumab with chemotherapy in patients with *RAS* wild-type metastatic colorectal cancer.

## Main

For patients with unresectable *RAS* wild-type (WT) recurrent or metastatic colorectal cancer (mCRC), standard first-line treatment includes chemotherapy combined with either an anti-epidermal growth factor receptor (EGFR) monoclonal antibody (for example, panitumumab or cetuximab) or an antivascular endothelial growth factor (VEGF) antibody (bevacizumab)^1–3^. The phase 3 PARADIGM trial (NCT02394795) in patients with unresectable *RAS* WT mCRC demonstrated longer overall survival (OS) with first-line panitumumab plus modified 5-fluorouracil, l-leucovorin, oxaliplatin (mFOLFOX6) versus bevacizumab plus mFOLFOX6 in patients with left-sided primary tumors (that is, tumors originating from the descending colon, sigmoid colon, rectosigmoid and rectum; median OS: 37.9 versus 34.3 months, respectively; hazard ratio (HR), 0.82; *P* = 0.03) and in the overall patient population (36.2 versus 31.3 months; HR, 0.84; *P* = 0.03)^4^. Exploratory analyses showed poorer survival (20.2–23.2 months) in patients with tumors originating from the right side of the colorectum^4^. These results support panitumumab plus mFOLFOX6 as a preferred treatment option for patients with *RAS* WT and left-sided primary mCRC.

Differences in outcomes with anti-EGFR treatment in mCRC may be attributed to tumor genomic and molecular profiles associated with primary resistance to EGFR inhibition, such as the *BRAF* V600E mutation and high microsatellite instability (MSI-H), among others^5–7^. Based on 2022 guidelines from the American Society of Clinical Oncology, the decision to initiate anti-EGFR treatment should be guided by the primary tumor location and testing for *BRAF* and *RAS* (*KRAS* and *NRAS*) mutations and deficient mismatch repair or MSI^2^. Several other less common molecular alterations have been linked to primary resistance to EGFR inhibitors, including mutations in *PTEN* and *EGFR* extracellular domain (ECD), amplifications of *HER2* and *MET*, and fusions of *ALK*, *RET* and *NTRK1* (refs. ^8–13^). Whereas individual molecular markers have guided drug development and improved patient selection for many targeted therapies, testing for a combination of multiple molecular markers has the potential to guide more precise therapy selection for patients with mCRC^14^.

To allow for molecular negative hyperselection of patients most likely to benefit from anti-EGFR treatment, previous studies have worked towards establishing a testing panel that includes a broad array of rare genomic alterations linked to primary resistance to EGFR inhibition^15–18^. These studies demonstrated that detection of any of the panel-prespecified genetic alterations in tumor biopsy samples, as well as primary tumor sidedness, were predictive of clinical outcomes on anti-EGFR therapy^15–18^. Genotyping of tumors based on testing of circulating tumor DNA (ctDNA) released from cancer cells into the plasma (that is, liquid biopsy) is a minimally invasive alternative to tissue biopsy that may be particularly advantageous for identifying patients with mCRC who may benefit from anti-EGFR therapy^19–21^. In this prespecified exploratory biomarker analysis of the phase 3 PARADIGM trial (NCT02394834), we tested ctDNA in baseline plasma samples from more than 700 patients with *RAS* WT mCRC to investigate the utility of ctDNA-based negative hyperselection for predicting treatment outcomes with mFOLFOX6 combined with either panitumumab or bevacizumab.

## Results

### Patients

**Fig. 1:**
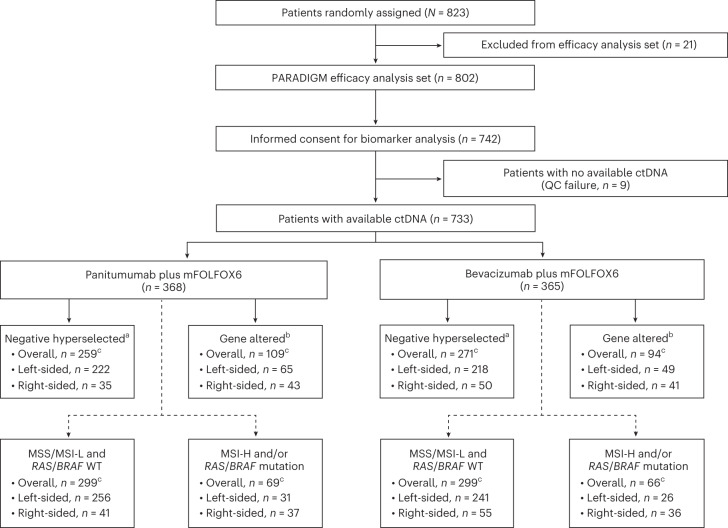
Patient flow chart for analysis of gene alteration status. ^a^‘Negative hyperselected’ was defined as plasma ctDNA being negative for all prespecified gene alterations, including mutations in *BRAF* V600E, *KRAS*, *PTEN*, *EGFR* ECD exons 1–16 and *NRAS*, amplifications of *HER2* and *MET*, and gene fusions of *RET*, *NRTK1* and *ALK*. ^b^‘Gene altered’ was defined as detection of any of the following in ctDNA: a mutation in *BRAF* V600E, *KRAS*, *PTEN*, *EGFR* ECD exons 1–16 and/or *NRAS*, amplification of *HER2* and/or *MET*, and gene fusion of *RET*, *NRTK1* and/or *ALK*. ^c^Some patients had multiple primary lesions on both the left and right sides. The dotted line represents an additional exploratory analysis assessing genetic alterations of MSS/MSI status and *RAS/BRAF* mutations based on guideline recommendations. ECD, extracellular domain; QC, quality control.

Of the 802 patients with *RAS* WT mCRC included in the PARADIGM efficacy analysis population, 733 patients (91.4%) provided informed consent for this biomarker study and had baseline blood plasma samples that were evaluable for ctDNA (Fig. 1). Among these 733 patients, 554 patients (75.6%) had left-sided primary tumors, 169 (23.1%) had right-sided primary tumors, and 10 (1.4%) had multiple primary lesions in both the left and right sides. For the biomarker-evaluable population, median follow-up as of the data cutoff date (14 January 2022) was 61.4 months (95% confidence interval (CI), 60.5–62.9 months) in the panitumumab + mFOLFOX6 group and 60.5 months (95% CI, 59.5–62.9 months) in the bevacizumab + mFOLFOX6 group.

Patient ctDNA was assessed for 90 mutations, 26 amplifications and 3 rearrangements in mCRC-related genes using a custom NGS-based panel (Methods). Maximum variant allele frequency is reported for all samples in Supplementary Table 1. We report results of a preplanned analysis for negative hyperselection, meaning plasma ctDNA was negative for all prespecified gene alterations associated with resistance to anti-EGFR antibody therapy^15,17,22–24^, including mutations in *BRAF* V600E, *KRAS*, *NRAS*, *PTEN* and *EGFR* ECD exons 1–16, amplifications of *HER2* and *MET*, and gene fusions of *RET*, *NRTK1* and *ALK*. A total of 530 patients (72.3%) met these negative hyperselection criteria (Table 1). Patients with left-sided primary tumors met negative hyperselection criteria at a higher rate (79.4%: 440 of 554 patients) than patients with right-sided primary tumors (50.3%: 85 of 169 patients).

**Table 1:** Demographics and baseline characteristics by negative hyperselection status

	Overall (*N* = 733)		Negative hyperselected^a^ (*n* = 530)		Gene altered^b^ (*n* = 203)	
	Panitumumab + mFOLFOX6 (*n* = 368)	Bevacizumab + mFOLFOX6 (*n* = 365)	Panitumumab + mFOLFOX6 (*n* = 259)	Bevacizumab + mFOLFOX6 (*n* = 271)	Panitumumab + mFOLFOX6 (*n* = 109)	Bevacizumab + mFOLFOX6 (*n* = 94)
Age category						
20–64 years	149 (40.5)	152 (41.6)	104 (40.2)	116 (42.8)	45 (41.3)	36 (38.3)
65–79 years	219 (59.5)	213 (58.4)	155 (59.8)	155 (57.2)	64 (58.7)	58 (61.7)
Sex						
Female	134 (36.4)	120 (32.9)	87 (33.6)	83 (30.6)	47 (43.1)	37 (39.4)
Male	234 (63.6)	245 (67.1)	172 (66.4)	188 (69.4)	62 (56.9)	57 (60.6)
ECOG PS						
0	304 (82.6)	288 (78.9)	220 (84.9)	213 (78.6)	84 (77.1)	75 (79.8)
1	63 (17.1)	77 (21.1)	39 (15.1)	58 (21.4)	24 (22.0)	19 (20.2)
Primary tumor location^c^						
Left side^d^ (*n* = 554)	287 (78.0)	267 (73.2)	222 (85.7)	218 (80.4)	65 (59.6)	49 (52.1)
Right side^e^ (*n* = 169)	78 (21.2)	91 (24.9)	35 (13.5)	50 (18.5)	43 (39.4)	41 (43.6)
Number of metastatic organs						
1	181 (49.2)	178 (48.8)	141 (54.4)	139 (51.3)	40 (36.7)	39 (41.5)
≥2	187 (50.8)	187 (51.2)	118 (45.6)	132 (48.7)	69 (63.3)	55 (58.5)
Metastatic site						
Liver	254 (69.0)	248 (67.9)	173 (66.8)	182 (67.2)	81 (74.3)	66 (70.2)
Liver only site of metastases	96 (26.1)	102 (27.9)	73 (28.2)	78 (28.8)	23 (21.1)	24 (25.5)
Previous primary tumor resection	222 (60.3)	244 (66.8)	166 (64.1)	184 (67.9)	56 (51.4)	60 (63.8)

Data are presented as *n* (%).

^a^‘Negative hyperselected’ was defined as plasma ctDNA being negative for all prespecified gene alterations, including mutations in *BRAF* V600E, *KRAS*, *PTEN*, *EGFR* ECD exons 1–16, and *NRAS*, amplifications of *HER2* and *MET*, and gene fusions of *RET*, *NRTK1* and *ALK*.

^b^‘Gene altered’ was defined as detection of any of the following in ctDNA: a mutation in *BRAF* V600E, *KRAS*, *PTEN*, *EGFR* ECD exons 1–16 and/or *NRAS*, amplification of *HER2* and/or *MET*, and gene fusion of *RET*, *NRTK1* and/or *ALK*.

^c^Some patients had multiple primary lesions on both the left and right sides.

^d^Primary tumors originating from the descending colon, sigmoid colon, rectosigmoid and rectum.

^e^Primary tumors originating from the right side of the colon, defined as cecum, ascending colon or transverse colon.

ECOG, Eastern Cooperative Oncology Group; PS, performance status.

**Fig. 2:**
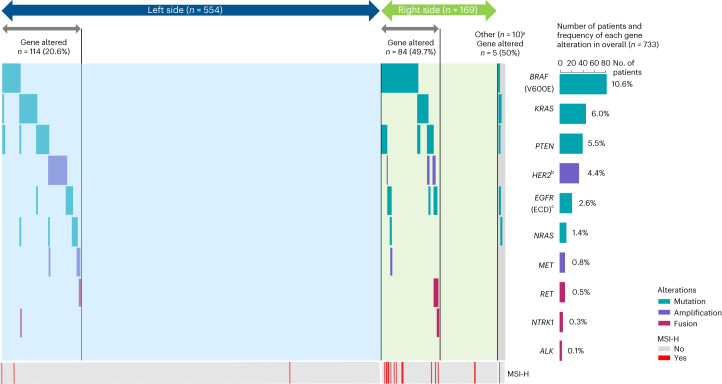
Oncoprint showing the incidence and co-occurrence of genomic alterations. ^a^Patients who had multiple primary lesions on both the left and right sides. ^b^The custom panel (Tak_Seq3) has a 1.25 threshold for *HER2* (thresholds were set based on noise in normal samples). ^c^*EGFR* (ECD): exons 1–16 (1–620).

Among the 203 (27.7%) patients with at least one gene alteration, the most common alterations were *BRAF* V600E mutation (10.6%), *KRAS* mutation (6.0%) and *PTEN* mutation (5.5%) (Fig. 2). Most patients had only one mutation in the left-sided (93.0%; 106 of 114 patients), right-sided (73.8%; 62 of 84 patients) and overall populations (84.2%; 171 of 203 patients), with co-occurrence of multiple mutations most common in right-sided mCRC (Fig. 2). The frequency of gene alterations is summarized by primary tumor location and treatment group in Extended Data Table 1.

### Outcomes by negative hyperselection status

#### Overall survival

**Fig. 3:**
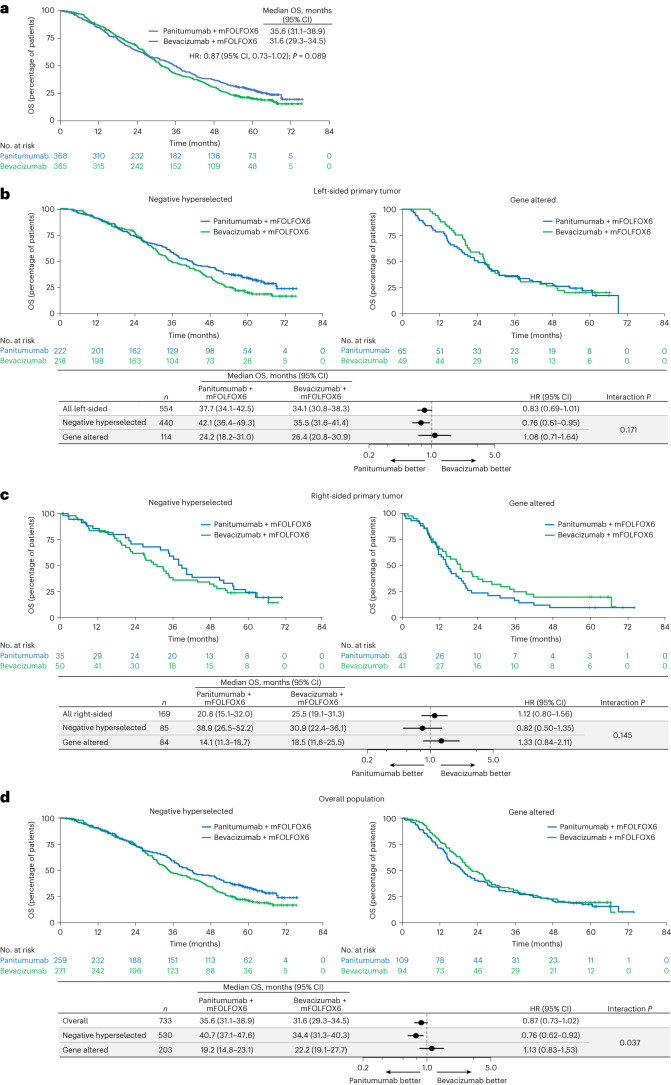
Overall survival in the biomarker-marker evaluable population overall and by negative hyperselection status. **a**, Kaplan–Meier estimates of OS in the overall biomarker-evaluable population (all ctDNA-evaluable patients). **b**–**d** Kaplan–Meier estimates of OS by negative hyperselection status in patients with left-sided primary tumors (**b**), patients with right-sided primary tumors (**c**) and the overall population (all ctDNA-evaluable patients) (**d**). The forest plots below the Kaplan–Meier plots in **b**, **c** and **d** show HR ± 95% CI. A Cox proportional hazard model without stratification factors was used to calculate HRs for group comparisons and *P* values for the interaction between negative hyperselection status and treatment group. Statistical tests were two-sided without adjustment for multiple comparisons.

For the total biomarker-evaluable population, median OS was 35.6 months (95% CI, 31.1–38.9 months) with panitumumab + mFOLFOX6 and 31.6 months (95% CI, 29.3–34.5 months) with bevacizumab + mFOLFOX6 (HR for death stratified by age and presence of liver metastasis: 0.87; 95% CI, 0.73–1.02; Fig. 3a). For patients meeting negative hyperselection criteria (that is, no gene alteration detected), OS was longer with panitumumab versus bevacizumab in patients with left-sided primary tumors (median 42.1 versus 35.5 months; HR, 0.76; 95% CI, 0.61–0.95; *P* value for interaction between treatment group and negative hyperselection status = 0.171; Fig. 3b), and there was a trend for longer OS with panitumumab versus bevacizumab in patients with right-sided tumors (38.9 versus 30.9 months; HR, 0.82; 95% CI, 0.50–1.35; interaction *P* = 0.145; Fig. 3c). In the overall negative hyperselected population, median OS was longer with panitumumab versus bevacizumab (40.7 versus 34.4 months; HR, 0.76; 95% CI, 0.62–0.92; interaction *P* = 0.037; Fig. 3d).

**Fig. 4:**
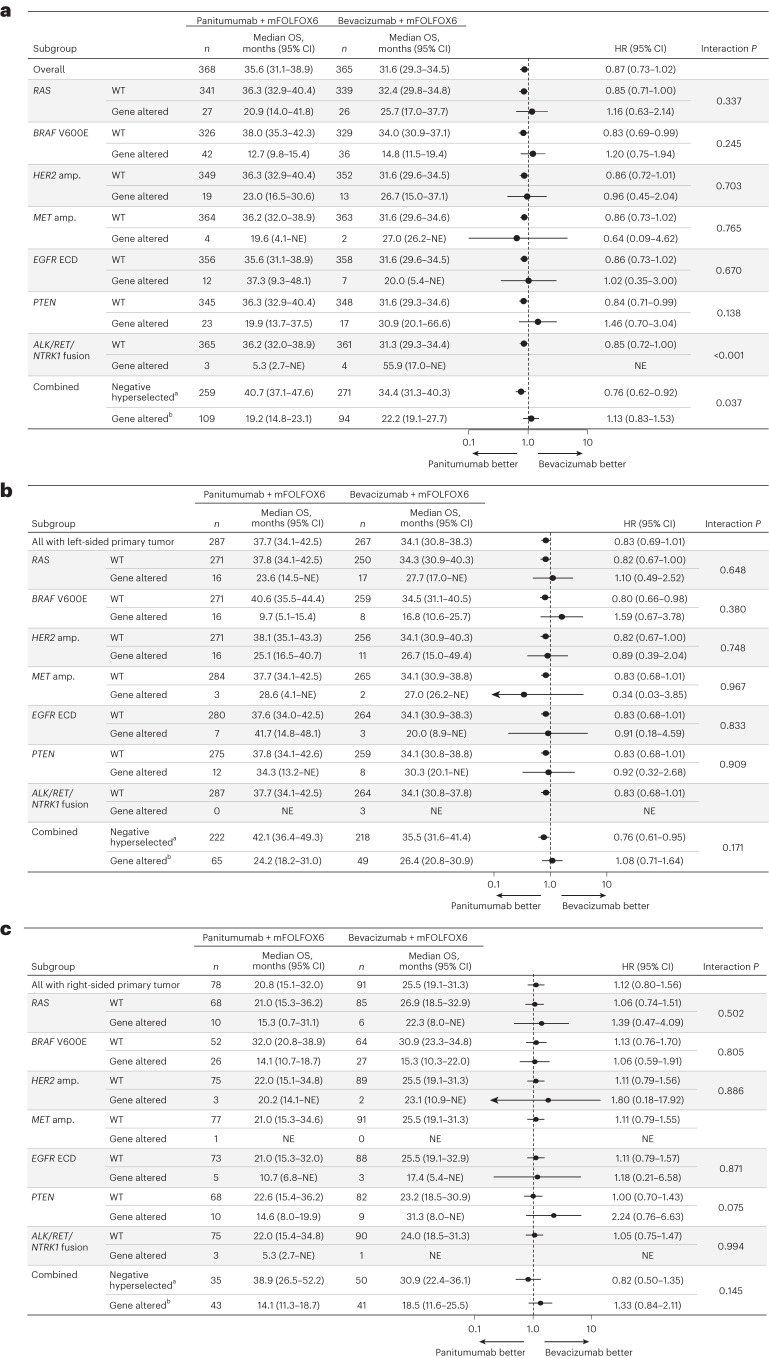
Overall survival by specific gene alteration. **a**–**c**, OS by specific gene alteration in the overall population (**a**), patients with left-sided primary tumors (**b**) and patients with right-sided primary tumors (**c**). Data plotted are HRs ± 95% CI. A Cox proportional hazard model without stratification factors was used to calculate HRs for group comparisons and *P* values for the interaction between negative hyperselection status and treatment group. Statistical tests were two-sided without adjustment for multiple comparisons. ^a^Negative hyperselected patients were WT for all of the following: *RAS*, *BRAF* V600E, *HER2* amp., *MET* amp., *EGFR* ECD, *PTEN* and *ALK/RET/NTRK1* fusion. ^b^Gene-altered patients had at least one of the following alterations: *RAS*, *BRAF* V600E, *HER2* amp., *MET* amp., *EGFR* ECD, *PTEN* or *ALK/RET/NTRK1* fusion. amp., amplification; NE, not estimable.

For patients with any gene alteration, median OS was similar or inferior with panitumumab versus bevacizumab regardless of primary tumor sidedness. Median OS with panitumumab versus bevacizumab in gene-altered patients was 24.2 versus 26.4 months in patients with left-sided primary tumors (HR, 1.08; 95% CI, 0.71–1.64; Fig. 3b), 14.1 versus 18.5 months in patients with right-sided primary tumors (HR, 1.33; 95% CI, 0.84–2.11; Fig. 3c) and 19.2 versus 22.2 months in the overall population (HR, 1.13; 95% CI, 0.83–1.53; Fig. 3d). Results of the subgroup analysis of OS by specific gene alterations are shown for the overall population in Fig. 4a, and for the left-sided and right-sided populations in Fig. 4b and 4c, respectively.

#### Progression-free survival

**Extended Data Fig. 1:**
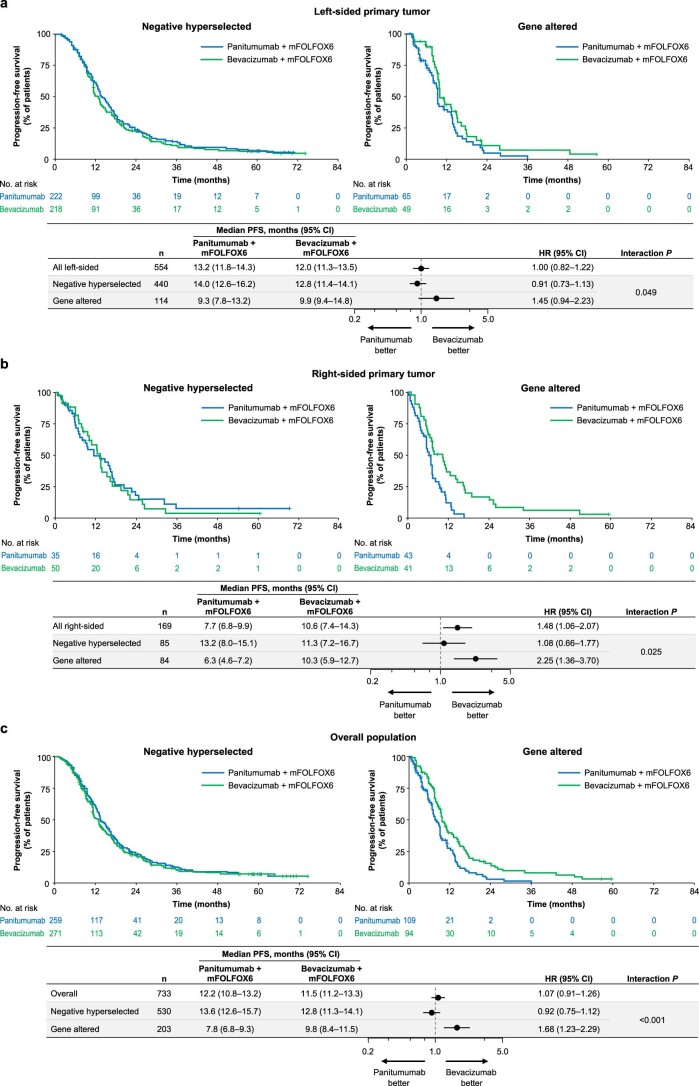
Progression-free survival (PFS) by negative hyperselection status. Kaplan–Meier estimates of PFS in (**a**) patients with left-sided primary tumors, (**b**) patients with right-sided primary tumors and (**c**) the overall population (all ctDNA-evaluable patients). The forest plots below each Kaplan–Meier plot show the HR ± 95% CI. A Cox proportional hazard model without stratification factors was used to calculate HRs for group comparisons and *P* values for the interaction between negative hyperselection status and treatment group. Statistical tests were two-sided without adjustment for multiple comparisons. ctDNA, circulating tumor DNA; HR, hazard ratio; mFOLFOX6, modified FOLFOX6.

For negative hyperselected patients, progression-free survival (PFS) was similar with panitumumab + mFOLFOX6 versus bevacizumab + mFOLFOX6 in the left-sided (14.0 versus 12.8 months; HR, 0.91; 95% CI, 0.73–1.13; *P* value for interaction between treatment group and negative hyperselection status = 0.049), right-sided (13.2 versus 11.3 months; HR, 1.08; 95% CI, 0.66–1.77; interaction *P* = 0.025) and overall populations (median, 13.6 versus 12.8 months; HR, 0.92; 95% CI, 0.75–1.12; interaction *P* < 0.001; Extended Data Fig. 1). For patients with any gene alteration, median PFS was similar with panitumumab and bevacizumab in the left-sided population (9.3 versus 9.9 months; HR, 1.45; 95% CI, 0.94–2.23) but shorter with panitumumab than bevacizumab in the right-sided (6.3 versus 10.3 months; HR, 2.25; 95% CI, 1.36–3.70) and overall populations (7.8 versus 9.8 months; HR, 1.68; 95% CI, 1.23–2.29; Extended Data Fig. 1).

#### Response rate

**Extended Data Fig. 2:**
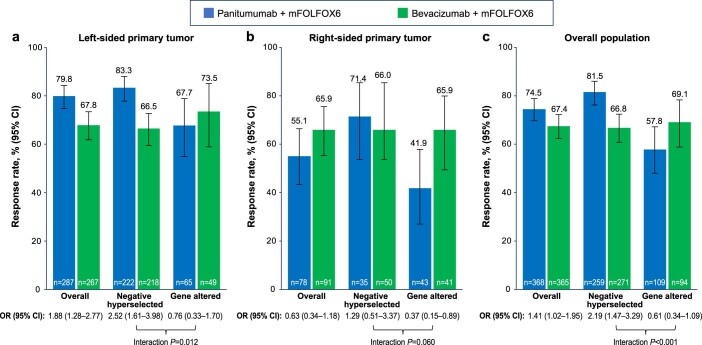
Response rate by negative hyperselection status. Response rates in (**a**) patients with left-sided primary tumors, (**b**) patients with right-sided primary tumors and (**c**) the overall population (all ctDNA-evaluable patients). Data plotted are percentages of patients with a response ± 95% CIs. ORs were calculated by logistic regression analysis. Statistical tests were two-sided without adjustment for multiple comparisons. ctDNA, circulating tumor DNA; mFOLFOX6, modified FOLFOX6; OR, odds ratio.

Among negative hyperselected patients, response rates were higher with panitumumab versus bevacizumab in the left-sided population (83.3% (95% CI, 77.8–88.0) versus 66.5% (95% CI, 59.8–72.7); odds ratio (OR), 2.52 (95% CI, 1.61–3.98); interaction *P* = 0.012), with a similar trend in the right-sided population (71.4% (95% CI, 53.7–85.4) versus 66.0% (95% CI, 51.2–78.8); OR, 1.29 (95% CI, 0.51–3.37); interaction *P* = 0.060; Extended Data Fig. 2), although the right-sided between-group difference was relatively small (+5.4%). In the overall negative hyperselected population, the response rate was higher with panitumumab (81.5% (95% CI, 76.2–86.0)) than with bevacizumab (66.8% (95% CI, 60.8–72.4)); OR, 2.19 (95% CI, 1.47–3.29); interaction *P* < 0.001). For patients with any gene alteration, the response rate was similar with panitumumab (67.7% (95% CI, 54.9–78.8)) versus bevacizumab (73.5% (95% CI, 58.9–85.1); OR, 0.76 (95% CI, 0.33–1.70)) in the left-sided population but lower with panitumumab (41.9% (95% CI, 27.0–57.9)) than bevacizumab (65.9% (95% CI, 49.4–79.9); OR, 0.37 (95% CI, 0.15–0.89)) in the right-sided population, with a similar trend in the overall gene-altered population (57.8% (95% CI, 48.0–67.2) versus 69.1% (95% CI, 58.8–78.3); OR, 0.61 (95% CI, 0.34–1.09); Extended Data Fig. 2).

#### Depth of response

**Extended Data Fig. 3:**
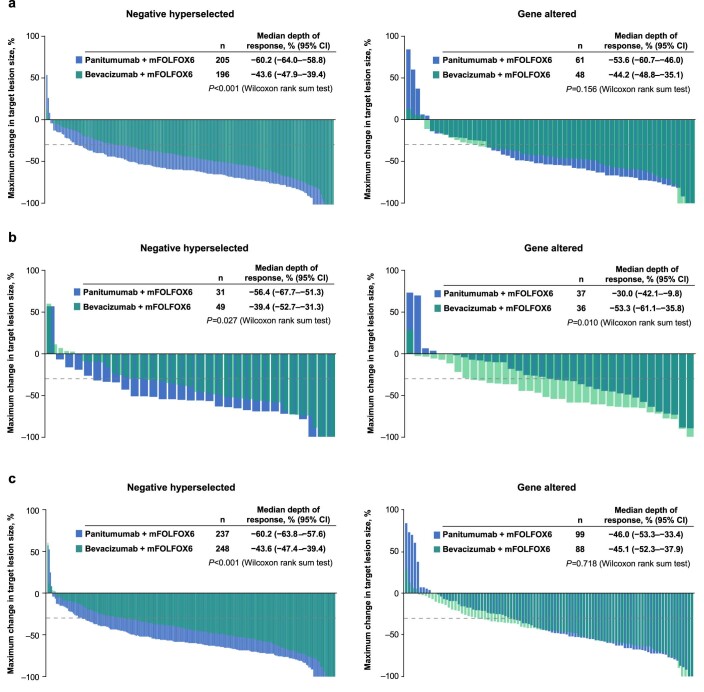
Depth of response by negative hyperselection status. Best change in target lesion size in negative hyperselected and gene altered (**a**) patients with left-sided primary tumors, (**b**) patients with right-sided primary tumors and (**c**) overall population (all ctDNA-evaluable patients). *P* values were calculated using a Wilcoxon rank sum test. Statistical tests were two-sided without adjustment for multiple comparisons. ctDNA, circulating tumor DNA; mFOLFOX6, modified FOLFOX6.

Median depth of response (maximum change in target lesion size) was greater with panitumumab versus bevacizumab among negative hyperselected patients with left-sided tumors (−60.2% (95% CI, −64.0 to −58.8) versus −43.6% (95% CI, −47.9 to −39.4)) and right-sided tumors (−56.4% (95% CI, −67.7 to −51.3) versus −39.4% (95% CI, −52.7 to −31.3)) and in the overall negative hyperselected population (−60.2% (95% CI, −63.8 to −57.6) versus −43.6% (95% CI, −47.4 to −39.4)). In gene-altered patients, depth of response was similar with panitumumab and bevacizumab in the left-sided (−53.6% (95% CI, −60.7 to −46.0) versus −44.2% (95% CI, −48.8 to −35.1)), right-sided (−30.0% (95% CI, −42.1 to −9.8) versus −53.3% (95% CI, −61.1 to −35.8)) and overall populations (−46.0% (95% CI, −53.3 to −33.4) versus −45.1% (95% CI, −52.3 to −37.9); Extended Data Fig. 3).

#### Curative resection rate

**Extended Data Fig. 4:**
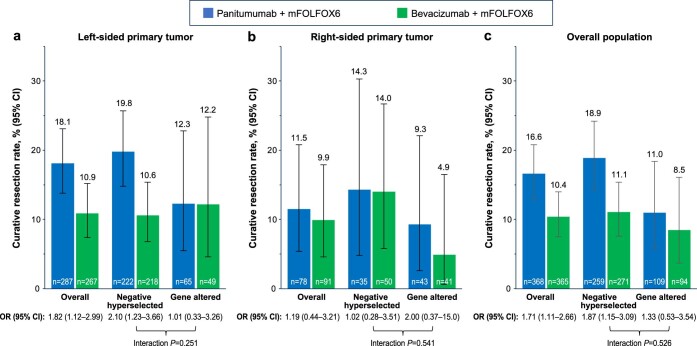
Curative resection rates by negative hyperselection status. Curative resection rates in (**a**) patients with left-sided primary tumors, (**b**) patients with right-sided primary tumors and (**c**) the overall population (all ctDNA-evaluable patients). Data plotted are percentages of patients with curative resection ± 95% CIs. ORs were calculated by logistic regression analysis. Statistical tests were two-sided without adjustment for multiple comparisons. ctDNA, circulating tumor DNA; mFOLFOX6, modified FOLFOX6; OR, odds ratio.

For negative hyperselected patients, the curative resection rate was higher with panitumumab versus bevacizumab in the left-sided population (19.8% (95% CI, 14.8–25.7) versus 10.6% (95% CI, 6.8–15.4); OR, 2.10 (95% CI, 1.23–3.66)) and similar between treatments in the right-sided population (14.3% (95% CI, 4.8–30.3) versus 14.0% (95% CI, 5.8–26.7); OR, 1.02 (95% CI, 0.28–3.51); Extended Data Fig. 4). In the overall negative hyperselected population, the curative resection rate was higher with panitumumab (18.9% (95% CI, 14.3–24.2)) than bevacizumab (11.1% (95% CI, 7.6–15.4); OR, 1.87 (95% CI, 1.15–3.09)). In patients with gene alterations, the curative resection rate was nearly identical with panitumumab and bevacizumab in the left-sided population (12.3% (95% CI, 5.5–22.8) versus 12.2% (95% CI, 4.6–24.8); OR, 1.01 (95% CI, 0.33–3.26)) but trended higher with panitumumab in the right-sided population (9.3% (95% CI, 2.6–22.1) versus 4.9% (95% CI, 0.6–16.5); OR, 2.00 (95% CI, 0.37–15.0); Extended Data Fig. 4). In the overall gene-altered population, the curative resection rate was 11.0% (95% CI, 5.8–18.4) with panitumumab and 8.5% (95% CI, 3.7–16.1) with bevacizumab (OR, 1.33 (95% CI, 0.53–3.54)).

### Outcomes by *RAS*/*BRAF* and microsatellite stability status

Current clinically adopted biomarkers (*RAS*/*BRAF* and microsatellite stable (MSS) status) in the first-line mCRC population were also explored. Among 733 ctDNA-evaluable patients, 598 patients (81.6%) were WT for *RAS* and *BRAF* and were MSS or had low microsatellite instability (MSI-L), including 497 (67.8%) with left-sided primary tumors and 96 (13.1%) with right-sided primary tumors (Fig. 1 and Supplementary Table 2). A total of 135 patients (18.4%) had *BRAF* V600E (78 patients (10.6%)) and/or *RAS* mutations (53 patients (7.2%)) and/or MSI-H (20 patients (2.7%)).

**Fig. 5:**
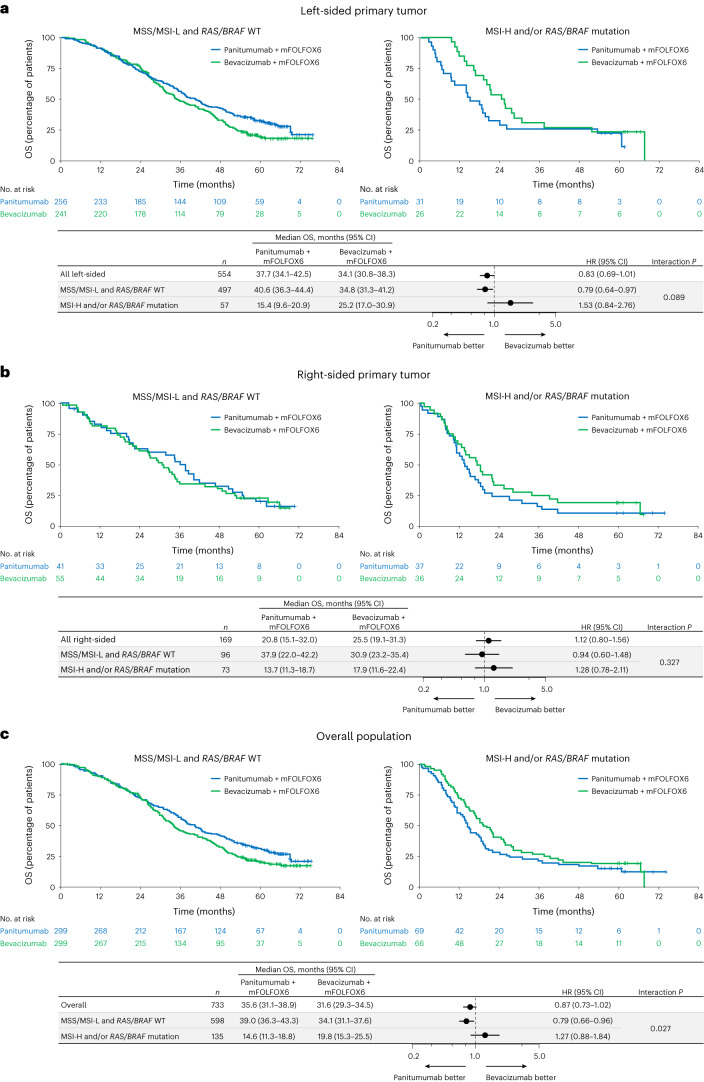
Overall survival by *RAS/BRAF* and MSS status. **a**–**c**, Kaplan–Meier estimates of OS in patients with left-sided primary tumors (**a**), patients with right-sided primary tumors (**b**) and the overall population (all ctDNA-evaluable patients) (**c**). The forest plots below each Kaplan–Meier plot show HR ± 95% CI. A Cox proportional hazard model without stratification factors was used to calculate HRs for group comparisons and *P* values for the interaction between negative hyperselection status and treatment group. Statistical tests were two-sided without adjustment for multiple comparisons.

Median OS between panitumumab versus bevacizumab in patients with *RAS/BRAF* WT and MSS/MSI-L was 40.6 versus 34.8 months (HR, 0.79; 95% CI, 0.64–0.97; interaction *P* = 0.089; Fig. 5a) in the left-sided, 37.9 versus 30.9 months (HR, 0.94; 95% CI, 0.60–1.48; interaction *P* = 0.327; Fig. 5b) in the right-sided and 39.0 versus 34.1 months (HR, 0.79; 95% CI, 0.66–0.96; interaction *P* = 0.027; Fig. 5c) in the overall populations. For patients with *RAS/BRAF* mutation or MSI-H, median OS was inferior or similar with panitumumab versus bevacizumab in the left-sided (15.4 versus 25.2 months; HR, 1.53; 95% CI, 0.84–2.76; Fig. 5a), right-sided (13.7 versus 17.9 months; HR, 1.28; 95% CI, 0.78–2.11; Fig. 5b) and overall populations (14.6 versus 19.8 months; HR, 1.27; 95% CI, 0.88–1.84; Fig. 5c).

**Extended Data Fig. 5:**
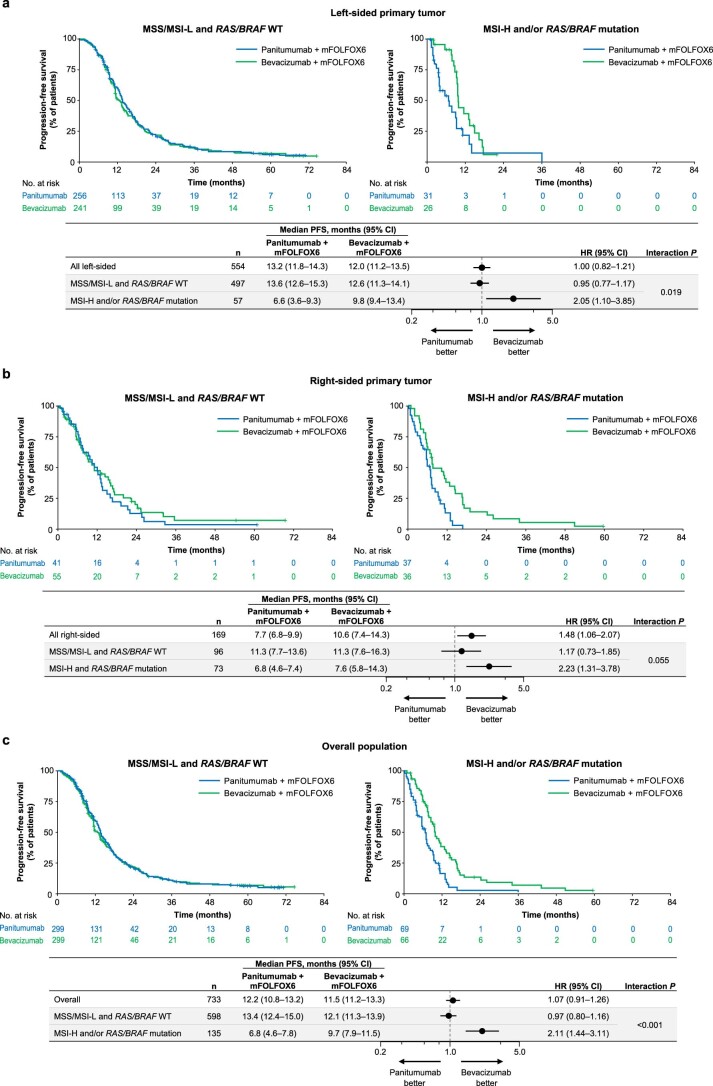
Progression-free survival (PFS) by *RAS*/*BRAF* and MSS status. Kaplan–Meier estimates of PFS in (**a**) patients with left-sided primary tumors, (**b**) patients with right-sided primary tumors and (**c**) the overall population (all ctDNA-evaluable patients). The forest plots below each Kaplan–Meier plot show the HR ± 95% CI. A Cox proportional hazard model without stratification factors was used to calculate HRs for group comparisons and *P* values for the interaction between negative hyperselection status and treatment group. Statistical tests were two-sided without adjustment for multiple comparisons. HR, hazard ratio; mFOLFOX6, modified FOLFOX6; MSI-H, microsatellite instability–high; MSI-L, microsatellite instability–low; MSS, microsatellite stable; WT, wild type.

**Extended Data Fig. 6:**
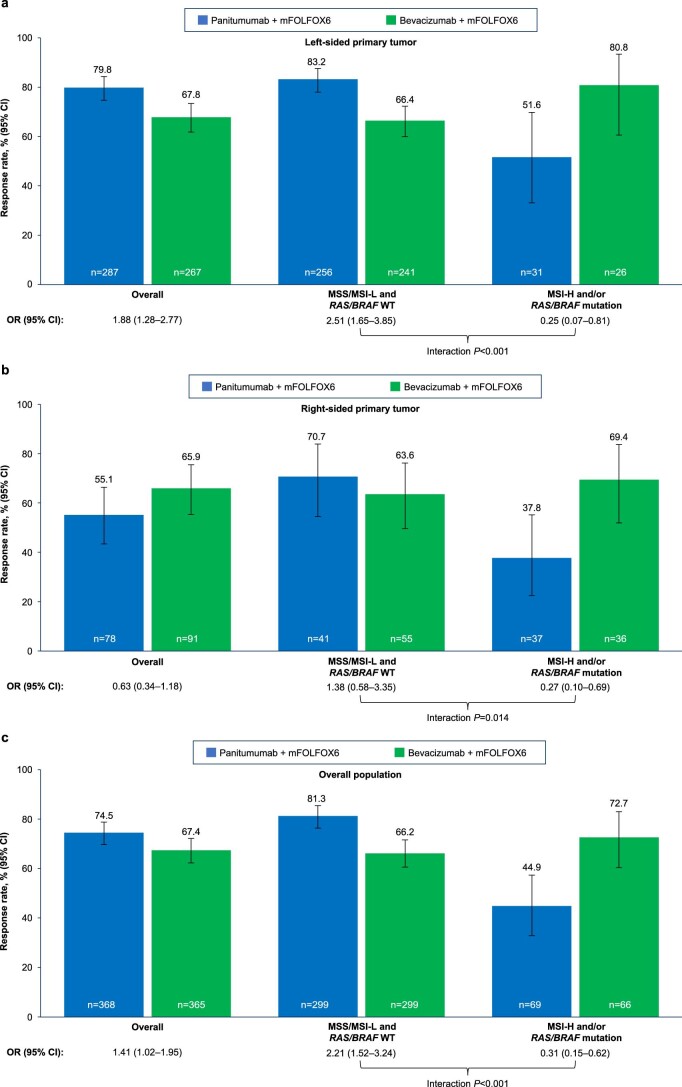
Response rates by *RAS*/*BRAF* and MSS status. Response rates in (**a**) patients with left-sided primary tumors, (**b**) patients with right-sided primary tumors and (**c**) the overall population (all ctDNA-evaluable patients). Data plotted are percentages of patients with a response ± 95% CIs. ORs were calculated by logistic regression analysis. Statistical tests were two-sided without adjustment for multiple comparisons. mFOLFOX6, modified FOLFOX6; MSI-H, microsatellite instability–high; MSI-L, microsatellite instability–low; MSS, microsatellite stable; OR, odds ratio; WT, wild type.

**Extended Data Fig. 7:**
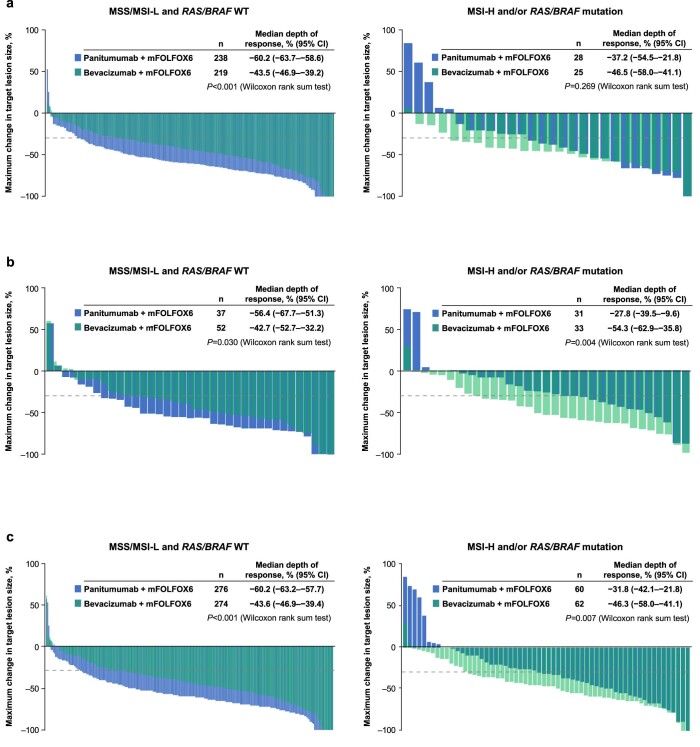
Depth of response by *RAS*/*BRAF* and MSS status. Best change in target lesion size in patients with MSS/MSI-L and *RAS*/*BRAF* WT or MSI-H and/or a *RAS*/*BRAF* mutation in (**a**) patients with left-sided primary tumors, (**b**) patients with right-sided primary tumors and (**c**) the overall population overall population (all ctDNA-evaluable patients). Dotted line at −30% indicated threshold for partial response per RECIST v1.1. *P* values were calculated using a Wilcoxon rank sum test. Statistical tests were two-sided without adjustment for multiple comparisons. ctDNA, circulating tumor DNA; mFOLFOX6, modified FOLFOX6; MSI-H, microsatellite instability–high; MSI-L, microsatellite instability–low; MSS, microsatellite stable; WT, wild type.

**Extended Data Fig. 8:**
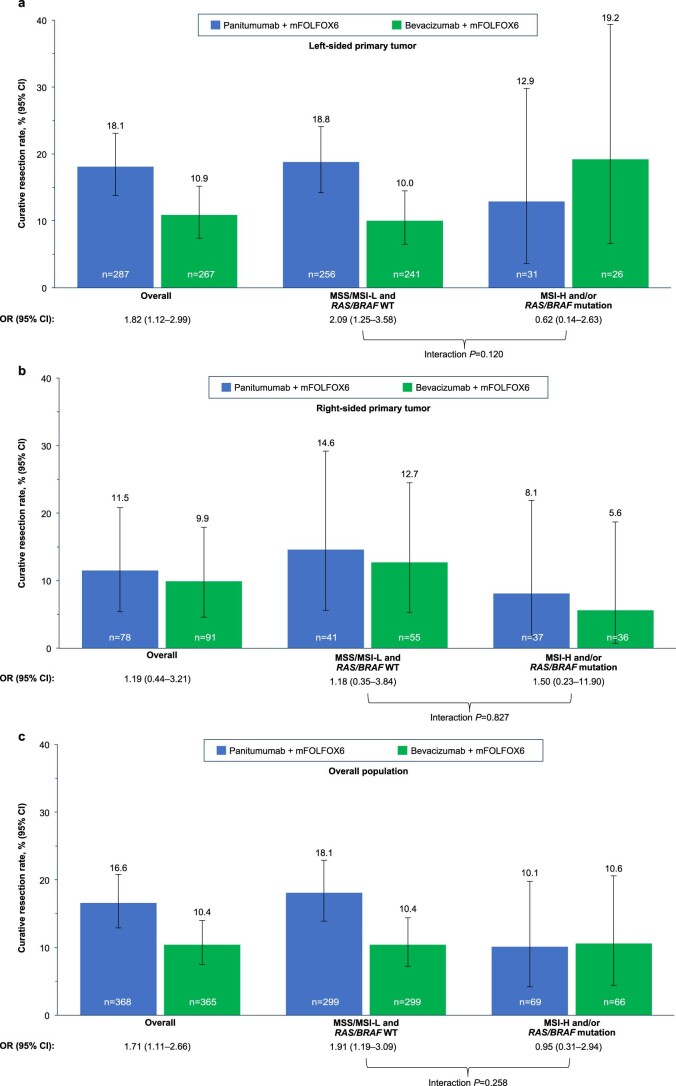
Curative resection rates by *RAS*/*BRAF* and microsatellite stability status. Curative resection rates in (**a**) patients with left-sided primary tumors, (**b**) patients with right-sided primary tumors and (**c**) the overall population (all ctDNA-evaluable patients). Data plotted are percentages of patients with curative resection ± 95% CIs. ORs were calculated by logistic regression analysis. Statistical tests were two-sided without adjustment for multiple comparisons. mFOLFOX6, modified FOLFOX6; MSI-H, microsatellite instability–high; MSI-L, microsatellite instability–low; MSS, microsatellite stable; WT, wild type.

Median PFS was comparable between panitumumab and bevacizumab for *RAS/BRAF* WT and MSS/MSI-L patients but tended to be shorter with panitumumab than bevacizumab in patients with a *RAS/BRAF* mutation and/or MSI-H, regardless of tumor sidedness (Extended Data Fig. 5). Antitumor response rates (Extended Data Fig. 6) and depth of response (Supplementary Table 3 and Extended Data Fig. 7) tended to improve with panitumumab versus bevacizumab in *RAS/BRAF* WT and MSS/MSI-L patients and poorer with panitumumab than bevacizumab for patients with a *RAS/BRAF* mutation and/or MSI-H, regardless of sidedness. Response rates are shown by specific gene alteration in Supplementary Fig. 1. Curative resection rates are shown by *RAS/BRAF* and MSS status in Extended Data Fig. 8.

### Safety

Adverse events occurred in 98.6% of patients in the biomarker population (Extended Data Table 2). The incidence of adverse events and grade 3 or higher adverse events was similar in negative hyperselected and gene-altered patients in each treatment group. A similar trend was observed when the triple-negative group (*RAS*/*BRAF* WT and MSS/MSI-L) was compared with the mutation (*RAS*/*BRAF* mutation and MSI-H) group (Supplementary Table 4).

## Discussion

In this prespecified exploratory biomarker analysis of the PARADIGM study, we investigated the potential prognostic and predictive role of hyperselecting patients for anti-EGFR treatment based on detection of a broad array of genetic alterations in plasma ctDNA in patients with *RAS* WT unresectable mCRC. Genetic alterations were chosen for evaluation due to reported associations with resistance to EGFR inhibition^8–13^, with an additional exploratory analysis assessing genetic alterations of MSS/MSI status and *RAS*/*BRAF* mutations based on guideline recommendations^2^. To our knowledge, this is the first report of negative hyperselection using ctDNA in a large phase 3 trial population (> 700 patients). Our results suggest that negative hyperselection using a validated and adequately sensitive plasma ctDNA assay may inform appropriate selection of patients for panitumumab treatment regardless of tumor sidedness (left versus right). For patients meeting negative hyperselection criteria, OS was prolonged with panitumumab + mFOLFOX6 compared with bevacizumab + mFOLFOX6 in patients with left-sided primary tumors (median 42.1 versus 35.5 months; HR, 0.76; 95% CI, 0.61–0.95). Higher rates of antitumor response (83.3% versus 66.5%), curative resection (19.8% versus 10.6%) and greater depth of response (median, −60.2% versus −43.6%) with panitumumab versus bevacizumab may have contributed to the improved OS in this negative hyperselected left-sided population.

The prevalence of any genetic alteration associated with resistance was higher among patients with right-sided (49.7%) versus left-sided (26.0%) primary tumors in this study, which is consistent with previous reports^6,7,15,16^. Of note, even in the patients with right-sided primary tumors, negative hyperselected patients showed numerically longer OS (38.9 versus 30.9 months; HR, 0.82; 95% CI, 0.50–1.35) as well as evidence of improved response rate (71.4% versus 66.6%) and depth of response (median, −56.4% versus −39.4%) with panitumumab versus bevacizumab. The wide 95% CIs for the HR in this correlation may be attributed to the low number of patients with right-sided tumors in our study population. Nevertheless, these results suggest that the primary tumor location may not be the sole determinant and support the notion that primary tumor location serves as a clinical surrogate marker reflecting the intricate molecular landscape of primary resistance to anti-EGFR antibodies. Although exploratory, our findings suggest that certain patients with right-sided colorectal cancer may benefit from first-line anti-EGFR antibodies with chemotherapy if negative hyperselection is feasible. Thus, while our data suggest that negative hyperselection status may be more informative for treatment selection than tumor sidedness, further investigations are necessary to confirm whether anti-EGFR antibody therapy is truly beneficial for negatively hyperselected patients with right-sided mCRC.

Although each of the candidate gene alterations in the negative hyperselection panel had a low frequency individually, prohibiting detection of an effect of individual mutations, it was possible to clarify the therapeutic effect by combining multiple gene alterations associated with resistance. Notably, few patients had multiple gene alterations, and the mutual exclusivity of the gene alterations indirectly supports their role as oncogenic drivers.

Anti-EGFR therapy with doublet chemotherapy is currently considered the treatment of choice for patients with left-sided, *RAS* and *BRAF* WT, and MSS mCRC^2,25^. When we stratified patients by the presence of MSI-H and/or a *RAS/BRAF* mutation, consistent with current American Society of Clinical Oncology and European Society for Medical Oncology guidelines^2,25^, patients appeared more appropriately selected for anti-EGFR therapy than when stratified by tumor sidedness alone. However, the ability to predict OS with panitumumab versus bevacizumab in the overall population appeared further improved with the more comprehensive negative hyperselection panel (median, 40.7 versus 34.4 months; HR, 0.76; 95% CI, 0.62–0.92) compared with the current gene testing recommendations (*RAS*/*BRAF* and MSS; median, 39.0 versus 34.1 months; HR, 0.79; 95% CI, 0.66–0.96). Moreover, among the right-sided population, the OS HR was changed from 0.94 in *RAS/BRAF* and MSS to 0.82 after negative hyperselection. Therefore, in addition to checking for the presence or absence of *RAS* and *BRAF* mutations and MSS status in both left- and right-sided primary tumors, expanding testing using the ctDNA-based gene panel evaluated in this study may prove useful for identifying patients for anti-EGFR antibody-based therapy.

There are some limitations to this study. Detailed analyses of tumor specimens have not yet been conducted. While the enrollment criteria required patients to have *RAS* WT tumor tissue based on local assessment using a validated test, a small proportion of patients was found to have *RAS* mutations (6.0% with *KRAS* and 1.4% with *NRAS* mutations) according to the ctDNA test. Patients with *RAS* alterations detected in ctDNA had poorer survival (median; panitumumab 20.9 months versus bevacizumab 25.7 months) than those with *RAS* WT in ctDNA (36.3 versus 32.4 months). A similar level of discordance between tumor tissue and ctDNA results was observed in the PERSEIDA trial, in which *RAS* mutations were detected in ctDNA at baseline in 12.6% of patients who were *RAS* WT according to tumor tissue biopsy at baseline^26^. There are three possible reasons for the observed discordance. First, spatial heterogeneity of tumor mutational profiles^27^ may have affected the tumor tissue results. Tissue assays using biopsy samples capture the tumor profile of a limited region, whereas ctDNA may capture a more complete set of tumor genetic information. Second, there were differences in the timing of sampling for tumor tissue and liquid biopsy. In some cases, tumor specimens were resected during the nonmetastatic stage, and *RAS* mutations may have emerged during the onset of metastatic disease after surgery. Third, assay sensitivities may have differed for the tissue and liquid biopsy assays. Whereas increasing the sensitivity of tissue next-generation sequencing (NGS) assays might improve the concordance rate, results would likely still be confounded by the spatial and timing differences in tumor and ctDNA sampling. Further investigation is required to better understand discrepancies in tumor tissue and ctDNA results and their implications. Nevertheless, previous studies have shown that plasma detection of *RAS* mutations has a high level of concordance with tissue biopsy results and a similar predictive level for benefit of anti-EGFR treatment as standard tumor tissue testing^19,20^. The detection of gene fusions and amplifications in ctDNA is technically challenging, limiting the ability of ctDNA assays to detect copy numbers and fusions. Factors related to clonal hematopoiesis of indeterminate potential were not specifically filtered, although ctDNA were cleaned using an algorithm validated to exclude false positives^28^. However, it was not possible to completely eliminate mutations related to clonal hematopoiesis. Furthermore, we cannot exclude the possibility that some patients may have been included in the negative hyperselection category because mutations were undetectable in plasma owing to low ctDNA shedding. However, the maximum variant allele frequency was ≥ 1.0% in 87% of samples, suggesting that the mutation profiles were consistent in ctDNA and tumor tissue in these cases^29^. Finally, this study was not statistically powered for comparisons between specific subgroups. Thus, additional studies are needed to confirm the findings.

In conclusion, our results show that negative hyperselection based on ctDNA-testing using a comprehensive panel of gene alterations associated with resistance to anti-EGFR therapy allows for the identification of patients with mCRC who may derive benefit from first-line treatment with panitumumab combined with chemotherapy.

## Methods

### Study design and patient population

The PARADIGM study (NCT02394795) was a randomized, open-label, phase 3 trial conducted at 197 sites in Japan between May 2015 and January 2022^4,30^. The study enrolled patients (age 20–79 years) with *RAS* WT unresectable adenocarcinoma originating in the colorectum who had not received previous chemotherapy for mCRC^30^. Screening for *KRAS and NRAS* mutations was performed using approved in vitro diagnostic tests^31^. *KRAS* and *NRAS* were required to be WT within exon 2 codons 12 and 13, exon 3 codons 59 and 61, and exon 4 codons 117 and 146 (refs. ^4,32^). Other key inclusion criteria were Eastern Cooperative Oncology Group performance status of 0 or 1 and presence of at least one evaluable lesion according to Response Evaluation Criteria in Solid Tumors version 1.1.

Patients were randomly allocated (1:1) to panitumumab + mFOLFOX6 or to bevacizumab + mFOLFOX6 (ref. ^4^). Randomization was stratified by study site, age (20–64 versus 65–79 years) and presence or absence of liver metastases^30^. The primary endpoint of PARADIGM was OS, which was tested hierarchically; first in patients with left-sided tumors and then in the overall population. Secondary endpoints were PFS, response rate, duration of response and curative resection rate. Depth of response was an exploratory endpoint. The data cutoff date for these data from the final analysis was 14 January 2022. Patient sex was determined by patient report and was not considered in the study design. Clinical data were collected using EDC Classic Rave (v.2020.2.0). Methods and clinical results of PARADIGM have been published previously^4,30^.

This exploratory biomarker analysis included patients who were enrolled in the main study (PARADIGM) and provided informed consent for the additional biomarker study (NCT02394834). The biomarker study protocol was approved by the institutional review boards or ethics committees at each participating center. The study was conducted in compliance with the protocol and ethical principles based on the Declaration of Helsinki, Ethical Guidelines on Medical Research Involving Human Subjects and International Council for Harmonisation of Technical Requirements for Pharmaceuticals for Human Use Guideline for Good Clinical Practice. The protocol and statistical analysis plan are available in the Supplementary Information.

### Molecular analyses

Baseline plasma ctDNA (> 10 ng ml^−1^ and > 10 nmol DNA) from patients enrolled in the biomarker study was assessed using a custom NGS-based panel (PlasmaSELECT-R 91, Personal Genome Diagnostics, Inc. Baltimore, MD). The panel was designed to detect 90 mutations, 26 amplifications and 3 rearrangements in mCRC-related genes, as well as MSI (Supplementary Table 5). The analytical sensitivity for detection was 92% and 83% for sequence mutations with mutant allele fraction of 0.10% and 0.20%, respectively, 100% (20% tumor purity) for high-level focal amplifications, 100% (0.10% tumor purity) for translocations, and 100% (0.50% tumor purity) for MSI. The specificity ranged from 99.9998% to 100.0%, depending on alteration type, and the reproducibility was 100% for liquid biopsy genotyping analyses for specimens meeting sample acceptance criteria. Targeted genomic regions spanned 250 kb. Prespecified gene alterations for negative hyperselection for anti-EGFR antibody therapy were *KRAS*, *NRAS*, *BRAF* (V600E), *PTEN* and ECD *EGFR* mutations (exons 1–16 (1–620)), *HER2* and *MET* amplifications, and *ALK*, *RET* and *NTRK1* fusions. The panel had a 1.25 threshold for *HER2*; thresholds were set based on noise in normal samples. An additional exploratory analysis based on current guideline recommendations regarding clinically relevant biomarkers assessed gene alterations of MSS/MSI-L versus MSI-H status and *RAS* (*KRAS*/*NRAS*) and *BRAF* V600E mutations.

### Statistical analysis

The association of negative hyperselection status (all negative versus gene altered (that is, any positive biomarker)) with OS, PFS, response rate, depth of response and curative resection rate was evaluated in patients who were included in the efficacy analysis and had evaluable baseline ctDNA samples. Efficacy outcomes were also evaluated according to *RAS*, *BRAF* (V600E) and MSI status (all negative versus any positive biomarker). OS was defined as the time from the day of randomization (day 1) until death from any cause. PFS was the time from the day of randomization until disease progression or death; for patients who underwent curative resection, the PFS period ended on the day when preoperative diagnostics confirmed no progressive disease. For patients who discontinued study treatment due to an adverse event or other reasons without disease progression or death, PFS was the time until progressive disease or death after subsequent therapy or the patient was censored at the last follow-up date^4^. The response rate was defined as the percentage of patients whose best overall response was either complete response or partial response. PFS and OS were analyzed according to the Kaplan–Meier method^4^.

Patient characteristics at baseline were summarized by treatment group, gene-altered status and tumor sidedness using descriptive statistics. A Cox proportional hazard model without stratification factors was used to calculate HRs, and 95% CIs for group comparisons and *P* values for the interaction between negative hyperselection status and treatment group. ORs for group comparisons of response rates and curative resection rates were calculated by logistic regression analysis. All statistical tests were two-sided without adjustment for multiple comparisons. Statistical analyses were conducted using R (v.4.0.5) using the following packages: gtsummary (v.1.7.0), survival (v.3.5-5), survminer (v.0.4.9), g gplot2 (v.3.4.2), forester (0.2.0) and Complex Heatmap (v.2.13.1).

### Reporting summary

Further information on research design is available in the Nature Portfolio Reporting Summary linked to this article.

## Online content

Any methods, additional references, Nature Portfolio reporting summaries, source data, extended data, supplementary information, acknowledgements, peer review information; details of author contributions and competing interests; and statements of data and code availability are available at 10.1038/s41591-023-02791-w.

### Supplementary information

Supplementary Information Supplementary Tables 1–5, Fig. 1, Protocol and Statistical Analysis Plan.

Reporting Summary

## Figures and Tables

**Extended Data Table 1:**
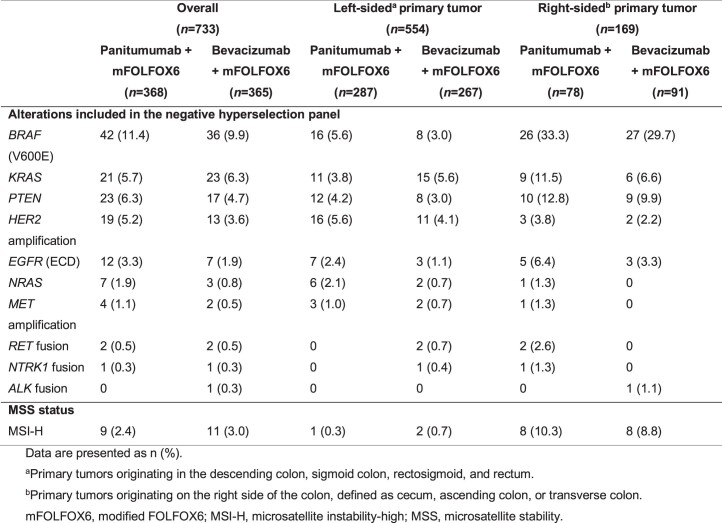
Incidence of genetic alterations by tumor sidedness and treatment group

**Extended Data Table 2:**
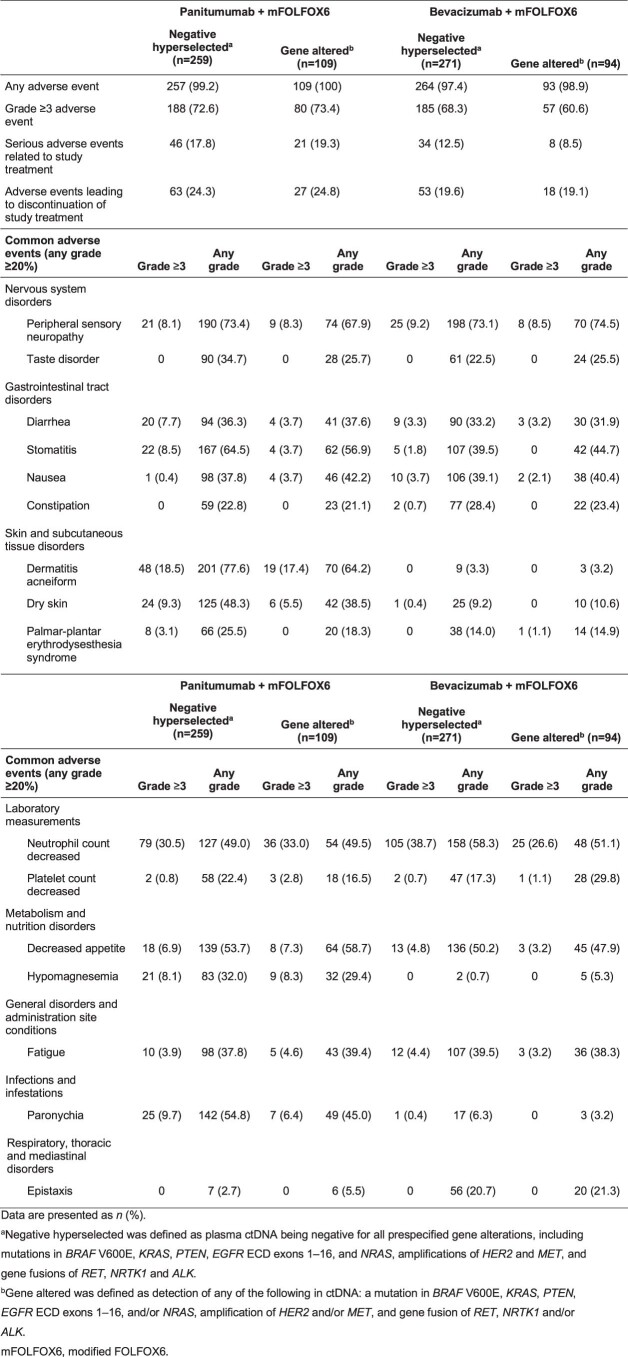
Adverse events

## Data Availability

The datasets, including individual participant data supporting the results reported in this article, will be made available within 3 months from initial request to researchers who provide a methodologically sound proposal. The initial contact for the request will be made with the corresponding author (K.S.). The data are not publicly available due to privacy/ethical restrictions and intellectual property reasons and will be provided after de-identification in compliance with applicable privacy laws, data protection and requirements for consent and anonymization. Researchers will be requested to execute the contract with Takeda Pharmaceutical Company Ltd. for the usage of the data.
