# Supplementary material for: Baseline ctDNA gene alterations as a biomarker of survival after panitumumab and chemotherapy in metastatic colorectal cancer
Source: Nat Med. 2024 Feb 12;30(3):730–9. doi: 10.1038/s41591-023-02791-w (PMC10957476; doi:10.1038/s41591-023-02791-w)
Supplement: Supplementary file 1 — Supplementary Tables 1–5, Fig. 1, Protocol and Statistical Analysis Plan. [file 41591_2023_2791_MOESM1_ESM.pdf]

nature medicine

Article https://doi.org/10.1038/s41591-023-02791-w

Baseline ctDNA gene alterations as a biomarker of survival after panitumumab and chemotherapy in metastatic colorectal cancer

In the format provided by the authors and unedited

SUPPLEMENTARY INFORMATION

SUPPLEMENTARY TABLES

Supplementary Table 1 | Maximum variant allele frequency (VAF) in all patient samples

Case #	Maximum VAF[a]
001	37.76%
002	4.53%
003	40.32%
004	39.07%
005	11.08%
006	52.26%
007	45.60%
008	6.53%
009	58.71%
010	17.00%
011	78.43%
012	25.11%
013	0.34%
014	5.39%
015	67.47%
016	51.47%
017	1.36%
018	36.72%
019	30.57%
020	5.97%
021	0.70%
022	31.22%
023	62.50%
024	2.66%
025	58.29%
026	29.24%
027	14.74%
028	41.77%
029	30.14%
030	48.22%
031	51.25%
032	11.90%
033	65.65%

Case #	Maximum VAF[a]
034	0.35%
035	67.25%
036	78.01%
037	36.78%
038	62.91%
039	55.23%
040	1.55%
041	40.44%
042	3.76%
043	1.29%
044	3.85%
045	32.19%
046	78.48%
047	17.86%
048	81.79%
049	1.49%
050	38.67%
051	2.75%
052	69.42%
053	1.16%
054	NA
055	1.76%
056	3.68%
057	9.18%
058	2.45%
059	43.21%
060	25.43%
061	0.37%
062	46.22%
063	39.42%
064	1.05%
065	50.51%
066	1.92%
067	NA
068	3.16%
069	5.19%
070	46.47%

Case #	Maximum VAF[a]
071	63.76%
072	7.62%
073	3.00%
074	9.05%
075	3.37%
076	7.36%
077	53.20%
078	33.32%
079	28.41%
080	0.28%
081	0.55%
082	76.80%
083	35.61%
084	12.96%
085	0.38%
086	22.20%
087	0.45%
088	46.02%
089	61.94%
090	59.72%
091	76.99%
092	0.30%
093	11.76%
094	3.27%
095	1.51%
096	40.49%
097	44.45%
098	44.48%
099	47.19%
100	25.92%
101	80.42%
102	41.47%
103	65.18%
104	63.73%
105	4.37%
106	46.17%
107	0.23%

Case #	Maximum VAF[a]
108	61.24%
109	34.84%
110	45.74%
111	63.25%
112	13.09%
113	65.82%
114	35.95%
115	1.14%
116	0.41%
117	43.30%
118	11.82%
119	23.85%
120	12.30%
121	81.39%
122	14.19%
123	42.26%
124	79.01%
125	45.47%
126	3.18%
127	43.68%
128	10.76%
129	40.12%
130	0.69%
131	2.29%
132	39.28%
133	35.93%
134	87.36%
135	38.82%
136	32.14%
137	26.77%
138	82.44%
139	60.80%
140	29.13%
141	2.60%
142	17.05%
143	0.52%
144	74.06%

**Case #**	**Maximum VAF[a]**
145	0.11%
146	1.58%
147	26.50%
148	6.94%
149	0.18%
150	49.34%
151	1.60%
152	NA
153	45.20%
154	12.47%
155	46.04%
156	99.78%
157	18.43%
158	24.10%
159	12.76%
160	1.89%
161	57.37%
162	47.23%
163	55.13%
164	1.11%
165	61.88%
166	48.90%
167	46.16%
168	0.68%
169	7.22%
170	36.33%
171	22.94%
172	56.45%
173	17.90%
174	17.40%
175	28.97%
176	1.31%
177	39.60%
178	70.46%
179	52.60%
180	11.42%
181	0.42%

**Case #**	**Maximum VAF[a]**
182	24.94%
183	62.00%
184	11.37%
185	39.62%
186	41.89%
187	12.77%
188	44.49%
189	0.24%
190	56.10%
191	30.82%
192	12.67%
193	0.87%
194	11.76%
195	66.74%
196	22.85%
197	45.30%
198	33.16%
199	45.34%
200	2.85%
201	52.62%
202	45.92%
203	16.10%
204	8.77%
205	68.99%
206	62.83%
207	0.32%
208	69.16%
209	5.42%
210	1.06%
211	0.70%
212	54.24%
213	16.46%
214	44.76%
215	5.97%
216	0.26%
217	13.31%
218	68.25%

Case #	Maximum VAF[a]
219	5.18%
220	15.74%
221	0.37%
222	NA
223	45.65%
224	0.96%
225	38.41%
226	2.64%
227	83.08%
228	6.80%
229	39.30%
230	68.33%
231	87.20%
232	0.59%
233	6.76%
234	12.80%
235	47.97%
236	59.11%
237	7.94%
238	9.75%
239	46.78%
240	45.37%
241	4.85%
242	78.26%
243	41.27%
244	89.27%
245	40.74%
246	2.55%
247	0.51%
248	42.05%
249	43.45%
250	26.23%
251	3.24%
252	0.25%
253	47.66%
254	45.18%
255	1.60%

Case #	Maximum VAF[a]
256	32.18%
257	36.94%
258	42.57%
259	13.75%
260	60.69%
261	46.70%
262	2.90%
263	2.07%
264	45.07%
265	NA
266	56.46%
267	33.84%
268	9.99%
269	39.50%
270	1.22%
271	4.00%
272	12.27%
273	1.92%
274	4.68%
275	4.99%
276	18.17%
277	7.25%
278	81.16%
279	94.15%
280	3.90%
281	38.52%
282	11.89%
283	2.76%
284	42.21%
285	63.75%
286	13.25%
287	11.15%
288	59.20%
289	4.37%
290	42.68%
291	10.76%
292	6.71%

Case #	Maximum VAF[a]
293	4.17%
294	42.22%
295	57.05%
296	39.68%
297	16.34%
298	71.14%
299	23.66%
300	39.10%
301	1.52%
302	0.25%
303	NA
304	67.29%
305	2.35%
306	0.27%
307	16.00%
308	23.61%
309	1.30%
310	4.43%
311	37.55%
312	44.29%
313	36.97%
314	35.36%
315	9.21%
316	47.05%
317	0.83%
318	59.91%
319	11.90%
320	39.17%
321	0.24%
322	99.61%
323	0.47%
324	20.55%
325	48.89%
326	42.85%
327	50.19%
328	0.31%
329	62.61%

**Case #**	**Maximum VAF[a]**
330	28.62%
331	5.84%
332	38.79%
333	2.76%
334	9.35%
335	43.09%
336	0.73%
337	71.53%
338	40.50%
339	33.82%
340	28.38%
341	0.49%
342	13.07%
343	46.61%
344	60.55%
345	9.07%
346	8.00%
347	10.25%
348	4.42%
349	47.95%
350	76.12%
351	32.45%
352	6.37%
353	47.63%
354	11.50%
355	19.77%
356	47.27%
357	40.85%
358	0.78%
359	0.17%
360	34.24%
361	30.09%
362	14.12%
363	31.74%
364	18.57%
365	21.63%
366	43.83%

**Case #**	**Maximum VAF[a]**
367	48.24%
368	40.38%
369	79.62%
370	0.30%
371	0.32%
372	32.66%
373	13.16%
374	0.80%
375	66.49%
376	5.53%
377	26.17%
378	39.34%
379	39.67%
380	NA
381	52.81%
382	55.73%
383	35.33%
384	45.75%
385	28.61%
386	3.33%
387	82.76%
388	4.41%
389	72.50%
390	2.57%
391	36.71%
392	1.52%
393	0.61%
394	70.26%
395	45.66%
396	NA
397	5.38%
398	50.89%
399	40.04%
400	57.65%
401	5.26%
402	38.76%
403	45.64%

Case #	Maximum VAF[a]
404	1.60%
405	NA
406	41.19%
407	61.71%
408	63.79%
409	1.42%
410	43.78%
411	0.27%
412	59.76%
413	45.05%
414	NA
415	5.58%
416	4.38%
417	2.12%
418	0.25%
419	21.39%
420	13.53%
421	2.49%
422	23.37%
423	21.04%
424	1.04%
425	12.19%
426	9.51%
427	26.42%
428	29.96%
429	8.01%
430	42.27%
431	4.82%
432	49.18%
433	38.59%
434	41.54%
435	47.49%
436	NA
437	1.24%
438	0.31%
439	4.26%
440	NA

**Case #**	**Maximum VAF[a]**
441	43.13%
442	56.35%
443	44.50%
444	31.69%
445	43.39%
446	NA
447	43.45%
448	11.74%
449	28.00%
450	1.04%
451	48.05%
452	16.80%
453	27.71%
454	54.93%
455	55.61%
456	0.36%
457	34.22%
458	73.00%
459	10.31%
460	54.62%
461	NA
462	4.78%
463	NA
464	45.28%
465	64.66%
466	29.58%
467	8.36%
468	0.29%
469	26.13%
470	55.97%
471	45.39%
472	67.89%
473	58.41%
474	40.31%
475	58.89%
476	0.79%
477	66.94%

Case #	Maximum VAF[a]
478	31.12%
479	57.05%
480	0.81%
481	15.03%
482	62.01%
483	79.20%
484	0.33%
485	40.50%
486	15.20%
487	65.41%
488	36.96%
489	45.13%
490	44.21%
491	47.88%
492	20.27%
493	85.33%
494	75.77%
495	4.50%
496	57.12%
497	11.14%
498	56.07%
499	35.01%
500	71.26%
501	58.17%
502	0.38%
503	2.84%
504	14.55%
505	67.69%
506	7.44%
507	21.22%
508	6.33%
509	1.53%
510	52.89%
511	54.97%
512	37.70%
513	4.44%
514	60.85%

Case #	Maximum VAF[a]
515	0.62%
516	25.13%
517	28.43%
518	19.73%
519	1.51%
520	8.58%
521	11.75%
522	32.70%
523	0.50%
524	0.40%
525	43.18%
526	68.06%
527	45.04%
528	47.82%
529	8.62%
530	77.02%
531	40.74%
532	56.39%
533	17.15%
534	55.76%
535	37.15%
536	99.80%
537	1.26%
538	0.92%
539	69.99%
540	3.01%
541	8.27%
542	0.72%
543	7.65%
544	20.90%
545	11.17%
546	0.45%
547	4.37%
548	27.67%
549	46.49%
550	8.88%
551	36.48%

Case #	Maximum VAF[a]
552	NA
553	34.68%
554	2.38%
555	78.56%
556	67.29%
557	44.88%
558	0.56%
559	0.26%
560	4.02%
561	0.36%
562	18.02%
563	1.15%
564	41.35%
565	63.52%
566	0.48%
567	1.92%
568	40.86%
569	65.38%
570	18.18%
571	36.91%
572	20.97%
573	8.14%
574	52.71%
575	46.23%
576	1.72%
577	5.05%
578	62.82%
579	29.24%
580	9.55%
581	2.29%
582	37.17%
583	38.14%
584	39.75%
585	41.70%
586	1.87%
587	45.95%
588	6.67%

Case #	Maximum VAF[a]
589	30.63%
590	1.54%
591	79.74%
592	40.51%
593	11.51%
594	53.92%
595	2.28%
596	74.78%
597	35.16%
598	38.30%
599	8.09%
600	66.36%
601	35.95%
602	0.28%
603	0.23%
604	58.76%
605	57.66%
606	2.62%
607	48.73%
608	48.27%
609	0.43%
610	63.08%
611	22.54%
612	0.75%
613	84.67%
614	37.73%
615	5.47%
616	30.43%
617	1.92%
618	39.33%
619	16.43%
620	47.21%
621	2.95%
622	4.81%
623	0.17%
624	0.48%
625	38.81%

Case #	Maximum VAF[a]
626	50.10%
627	5.08%
628	73.25%
629	6.49%
630	76.46%
631	60.43%
632	71.03%
633	0.83%
634	3.66%
635	4.40%
636	56.27%
637	43.81%
638	56.13%
639	0.72%
640	0.17%
641	0.62%
642	74.84%
643	62.28%
644	57.70%
645	15.99%
646	1.82%
647	26.13%
648	72.62%
649	52.15%
650	65.89%
651	47.05%
652	12.15%
653	46.69%
654	43.74%
655	2.97%
656	22.03%
657	99.83%
658	40.84%
659	7.82%
660	0.11%
661	39.68%
662	28.24%

Case #	Maximum VAF[a]
663	58.98%
664	4.42%
665	6.31%
666	38.53%
667	7.35%
668	44.75%
669	14.62%
670	15.45%
671	0.59%
672	23.29%
673	3.29%
674	10.53%
675	0.46%
676	4.02%
677	0.83%
678	13.77%
679	41.12%
680	2.14%
681	43.02%
682	9.50%
683	29.37%
684	40.61%
685	39.22%
686	0.76%
687	8.53%
688	45.88%
689	0.40%
690	0.39%
691	69.70%
692	18.51%
693	26.23%
694	NA
695	64.22%
696	41.72%
697	24.26%
698	15.17%
699	3.11%

Case #	Maximum VAF[a]
700	71.30%
701	41.35%
702	35.35%
703	6.19%
704	39.39%
705	28.40%
706	NA
707	0.60%
708	46.87%
709	68.19%
710	58.72%
711	43.33%
712	61.19%
713	1.29%
714	2.04%
715	1.35%
716	18.56%
717	42.71%
718	11.59%
719	46.55%
720	1.26%
721	13.42%
722	13.52%
723	1.21%
724	2.66%
725	6.24%
726	20.49%
727	0.41%
728	21.44%
729	40.61%
730	26.67%
731	0.68%
732	26.13%
733	37.96%

[a]The maximum variant allele frequency (VAF) was estimated using the surrogate for ctDNA fraction.

Supplementary Table 2 | Demographics and baseline characteristics by *RAS/BRAF*/MSS status

	Left-sided[a] primary tumor (n=554)				Right-sided[b] primary tumor (n=169)				Overall population (n=733)[c]			
	MSS/MSI-L and *RAS/BRAF* WT (n=497)		MSI-H and/or *RAS/BRAF* mutation (n=57)		MSS/MSI-L and *RAS/BRAF* WT (n=96)		MSI-H and/or *RAS/BRAF* mutation (n=73)		MSS/MSI-L and *RAS/BRAF* WT (n=598)		MSI-H and/or *RAS/BRAF* mutation (n=135)	
**Characteristic**	**PAN n=256**	**BEV n=241**	**PAN n=31**	**BEV n=26**	**PAN n=41**	**BEV n=55**	**PAN n=37**	**BEV n=36**	**PAN n=299**	**BEV n=299**	**PAN n=69**	**BEV n=66**
Age category, y												
20–64	109 (42.6)	102 (42.3)	15 (48.4)	12 (46.2)	13 (31.7)	25 (45.5)	12 (32.4)	11 (30.6)	122 (40.8)	128 (42.8)	27 (39.1)	24 (36.4)
65–79	147 (57.4)	139 (57.7)	16 (51.6)	14 (53.8)	28 (68.3)	30 (54.5)	25 (67.6)	25 (69.4)	177 (59.2)	171 (57.2)	42 (60.9)	42 (63.6)
Female	81 (31.6)	71 (29.5)	13 (41.9)	10 (38.5)	18 (43.9)	19 (34.5)	21 (56.8)	19 (52.8)	99 (33.1)	90 (30.1)	35 (50.7)	30 (45.5)
ECOG PS												
0	215 (84.0)	189 (78.4)	26 (83.9)	21 (80.8)	34 (82.9)	42 (76.4)	28 (75.7)	30 (83.3)	250 (83.6)	234 (78.3)	54 (78.3)	54 (81.8)
1	41 (16.0)	52 (21.6)	5 (16.1)	5 (19.2)	7 (17.1)	13 (23.6)	8 (21.6)	6 (16.7)	49 (16.4)	65 (21.7)	14 (20.3)	12 (18.2)
Primary tumor location												
Left side[a]	256 (100)	241 (100)	31 (100)	26 (100)	0	0	0	0	256 (85.6)	241 (80.6)	31 (44.9)	26 (39.4)
Right side[b]	0	0	0	0	41 (100)	55 (100)	37 (100)	36 (100)	41 (13.7)	55 (18.4)	37 (53.6)	36 (54.5)
Number of metastatic organs												
1	131 (51.2)	124 (51.5)	10 (32.3)	11 (42.3)	22 (53.7)	25 (45.5)	17 (45.9)	15 (41.7)	154 (51.5)	151 (50.5)	27 (39.1)	27 (40.9)
≥2	125 (48.8)	117 (48.5)	21 (67.7)	15 (57.7)	19 (46.3)	30 (54.5)	20 (54.1)	21 (58.3)	145 (48.5)	148 (49.5)	42 (60.9)	39 (59.1)
Liver metastasis	184 (71.9)	166 (68.9)	24 (77.4)	19 (73.1)	23 (56.1)	36 (65.5)	22 (59.5)	21 (58.3)	208 (69.6)	46 (66.7)	205 (68.6)	43 (65.2)
Liver as only metastatic site	75 (29.3)	72 (29.9)	7 (22.6)	8 (30.8)	8 (19.5)	15 (27.3)	5 (13.5)	4 (11.1)	84 (28.1)	89 (29.8)	12 (17.4)	13 (19.7)
Prior primary tumor resection	159 (62.1)	159 (66.0)	12 (38.7)	16 (61.5)	29 (70.7)	37 (67.3)	20 (54.1)	26 (72.2)	190 (63.5)	199 (66.6)	32 (46.4)	45 (68.2)

Data are presented as *n* (%).

[a]Primary tumors originating in the descending colon, sigmoid colon, rectosigmoid, and rectum.

[b]Primary tumors originating on the right side of the colon, defined as cecum, ascending colon, or transverse colon.

[c]Some patients had multiple primary lesions in both the left and right sides.

BEV, bevacizumab + mFOLFOX6; ECOG, Eastern Cooperative Oncology Group; mFOLFOX6, modified FOLFOX6; MSI-H, microsatellite instability–high; MSI-L, microsatellite instability–low; MSS, microsatellite stable; PAN, panitumumab + mFOLFOX6; PS, performance status; WT, wild type.

Supplementary Table 3 | Depth of response by tumor sidedness, treatment group, and *RAS/BRAF*/MSS status

	Median depth of response, % (95%CI)	
	**Panitumumab + mFOLFOX6**	**Bevacizumab + mFOLFOX6**
Any-sided, overall	(*n*=336)	(*n*=336)
	–57.6 (–60.2, –54.5)	–44.4 (-46.9, -41.1)
MSS/MSI-L and *RAS/BRAF* WT	(*n*=276)	(*n*=274)
	–60.2 (−63.2, −57.7)	–43.6 (-46.9, -39.4)
MSI-H and or *RAS/BRAF* mutation	(*n*=60)	(*n*=62)
	–31.8 (−42.1, −21.8)	–46.3 (-58.0, -41.1)
Left-sided, overall	(*n*=266)	(*n*=244)
	–59.4 (-61.7, -57.0)	–43.8 (-46.7, -40.7)
MSS/MSI-L and *RAS/BRAF* WT	(*n*=238)	(*n*=219)
	–60.2 (-63.7, -58.6)	–43.5 (-46.9, -39.2)
MSI-H and or *RAS/BRAF* mutation	(*n*=28)	(*n*=25)
	–37.2 (-54.5, -21.8)	–46.5 (-58.0, -41.1)
Right-sided, overall	(*n*=68)	(*n*=85)
	–43.9 (-54.8, -32.1)	–46.3 (-54.3, -35.7)
MSS/MSI-L and *RAS/BRAF* WT	(*n*=37)	(*n*=52)
	–56.4 (-67.7, -51.3)	–42.7 (-52.7, -32.2)
MSI-H and or *RAS/BRAF* mutation	(*n*=31)	(*n*=33)
	–27.8 (-39.5, -9.6)	–54.3 (-62.9, -35.8)

mFOLFOX6, modified FOLFOX6; MSI-H, microsatellite instability–high; MSI-L, microsatellite instability–low; MSS, microsatellite stable; WT, wild type.

Supplementary Table 4 | Adverse events by *RAS*/*BRAF* and MSI status

	Panitumumab + mFOLFOX6				Bevacizumab + mFOLFOX6			
	***RAS*/*BRAF* WT and MSS/MSI-L (n=299)**		**MSI-H and/or *RAS*/*BRAF* mutation (n=69)**		***RAS*/*BRAF* WT and MSS/MSI-L (n=299)**		**MSI-H and/or *RAS*/*BRAF* mutation (n=66)**	
Any adverse event	297 (99.3)		69 (100)		292 (97.7)		65 (98.5)	
Grade ≥3 adverse event	219 (73.2)		49 (71.0)		200 (66.9)		42 (63.6)	
Serious adverse events related to study treatment	56 (18.7)		11 (15.9)		36 (12.0)		6 (9.1)	
Adverse events leading to discontinuation of study treatment	74 (24.7)		16 (23.2)		59 (19.7)		12 (18.2)	
**Common adverse events (any grade ≥20%)**	**Grade ≥3**	**Any grade**	**Grade ≥3**	**Any grade**	**Grade ≥3**	**Any grade**	**Grade ≥3**	**Any grade**
Nervous system disorders								
Peripheral sensory neuropathy	23 (7.7)	216 (72.2)	7 (10.1)	48 (69.6)	25 (8.4)	220 (73.6)	8 (12.1)	48 (72.7)
Taste disorder	0	99 (33.1)	0	19 (27.5)	0	69 (23.1)	0	16 (24.2)
Gastrointestinal tract disorders								
Diarrhea	22 (7.4)	108 (36.1)	2 (2.9)	27 (39.1)	10 (3.3)	100 (33.4)	2 (3.0)	20 (30.3)
Stomatitis	24 (8.0)	191 (63.9)	2 (2.9)	38 (55.1)	5 (1.7)	121 (40.5)	0	28 (42.4)
Nausea	2 (0.7)	115 (38.5)	3 (4.3)	29 (42.0)	12 (4.0)	117 (39.1)	0	27 (40.9)
Constipation	0	66 (22.1)	0	16 (23.2)	0	82 (27.4)	0	17 (25.8)
Skin and subcutaneous tissue disorders								
Dermatitis acneiform	59 (19.7)	230 (76.9)	8 (11.6)	41 (59.4)	0	10 (3.3)	0	2 (3.0)
Dry skin	25 (8.4)	138 (46.2)	5 (7.2)	29 (42.0)	1 (0.3)	29 (9.7)	0	6 (9.1)
Palmar-plantar erythrodysesthesia syndrome	8 (2.7)	75 (25.1)	0	11 (15.9)	1 (0.3)	41 (13.7)	0	11 (16.7)

	**Panitumumab + mFOLFOX6**				**Bevacizumab + mFOLFOX6**			
	***RAS*/*BRAF* WT and MSS/MSI-L (n=299)**		**MSI-H and/or *RAS*/*BRAF* mutation (n=69)**		***RAS*/*BRAF* WT and MSS/MSI-L (n=299)**		**MSI-H and/or *RAS*/*BRAF* mutation (n=66)**	
**Common adverse events (any grade ≥20%)**	**Grade ≥3**	**Any grade**	**Grade ≥3**	**Any grade**	**Grade ≥3**	**Any grade**	**Grade ≥3**	**Any grade**
Laboratory measurements								
Neutrophil count decreased	91 (30.4)	150 (50.2)	24 (34.8)	31 (44.9)	110 (36.8)	169 (56.5)	20 (30.3)	37 (56.1)
Platelet count decreased	3 (1.0)	66 (22.1)	2 (2.9)	10 (14.5)	2 (0.7)	56 (18.7)	1 (1.5)	19 (28.8)
Metabolism and nutrition disorders								
Decreased appetite	22 (7.4)	164 (54.8)	4 (5.8)	39 (56.5)	15 (5.0)	148 (49.5)	1 (1.5)	33 (50.0)
Hypomagnesemia	27 (9.0)	96 (32.1)	3 (4.3)	19 (27.5)	0	2 (0.7)	0	5 (7.6)
General disorders and administration site conditions								
Fatigue	11 (3.7)	114 (38.1)	4 (5.8)	27 (39.1)	13 (4.3)	120 (40.1)	2 (3.0)	23 (34.8)
Infections and infestations								
Paronychia	29 (9.7)	163 (54.5)	3 (4.3)	28 (40.6)	1 (0.3)	18 (6.0)	0	2 (3.0)
Respiratory, thoracic and mediastinal disorders								
Epistaxis	0	10 (3.3)	0	3 (4.3)	0	62 (20.7)	0	14 (21.2)

Data are presented as *n* (%).

mFOLFOX6, modified FOLFOX6; MSI-H, microsatellite instability–high; MSI-L, microsatellite instability–low; MSS, microsatellite stable; WT, wild type.

Supplementary Table 5 | Gene alterations assessed in the PGDx colorectal custom PlasmaSELECT Panel

**Sequence mutation analysis**					
ACVR2A	*CCND1*	*EZH2*	*IL6*	*MYC*	*ROS1*
AKT1	*CCND2*	*FBXW7*	*IRS2*	*NPM1*	*SMAD2*
ALK	*CCND3*	*FGFR1*	*JAK2*	*NRAS*	*SMAD3*
AMER1	*CDH1*	*FGFR2*	*JAK3*	*PDGFRA*	*SMAD4*
APC	*CDK4*	*FGFR3*	*KDR*	*PIK3CA*	*SMO*
AR	*CDK6*	*FLT3*	*KIT*	*PIK3CB*	*SOX9*
ARID1A	*CDKN2A*	*GAS6*	*KRAS*	*PIK3R1*	*SRC*
ARID1B	*CSF1R*	*GNA11*	*MAP2K1*	*POLD1*	*STAT3*
ARID2	*CTNNB1*	*GNAQ*	*MCL1*	*POLE*	*TCF7L2*
ATM	*DNMT3A*	*GNAS*	*MET*	*PTCH1*	*TERT*
AXIN2	*EGFR*	*HNF1A*	*MLH1*	*PTEN*	*TET2*
AXL	*ERBB2*	*HRAS*	*MPL*	*PTPN11*	*TGFBR1*
BCL2L1	*ERBB3*	*IDH1*	*MSH3*	*RB1*	*TGFBR2*
BRAF	*ERBB4*	*IDH2*	*MSH6*	*RET*	*TP53*
BRCA2	*ESR1*	*IGF2*	*MTOR*	*RNF43*	*VHL*
**Copy number analysis for assessment of amplifications**					
ALK	*CCND2*	*ERBB2*	*GAS6*	*MCL1*	*STAT3*
AXIN2	*CDK4*	*FGFR1*	*IGF2*	*MET*	
AXL	*CDK6*	*FGFR2*	*IL6*	*MYC*	
BCL2L1	*CSF1R*	*FGFR3*	*IRS2*	*PIK3CA*	
CCND1	*EGFR*	*FLT3*	*JAK3*	*SRC*	
**Rearrangement analysis**					
ALK	*NTRK1*	*RET*			
**Microsatellite instability analysis**					
BAT-25	*BAT-26*	*NR-21*	*NR-24*	*MONO-27*	

SUPPLEMENTARY FIGURE

Supplementary Fig. 1 | Response rate by specific gene alteration in the overall population. Data plotted are percentages of patients with a response ± 95% CIs. ORs were calculated by logistic regression analysis. Statistical tests were two-sided without adjustment for multiple comparisons. mFOLFOX6, modified FOLFOX6; MSI-H, microsatellite instability–high; MSI-L, microsatellite instability–low; MSS, microsatellite stable; OR, odds ratio; WT, wild type.

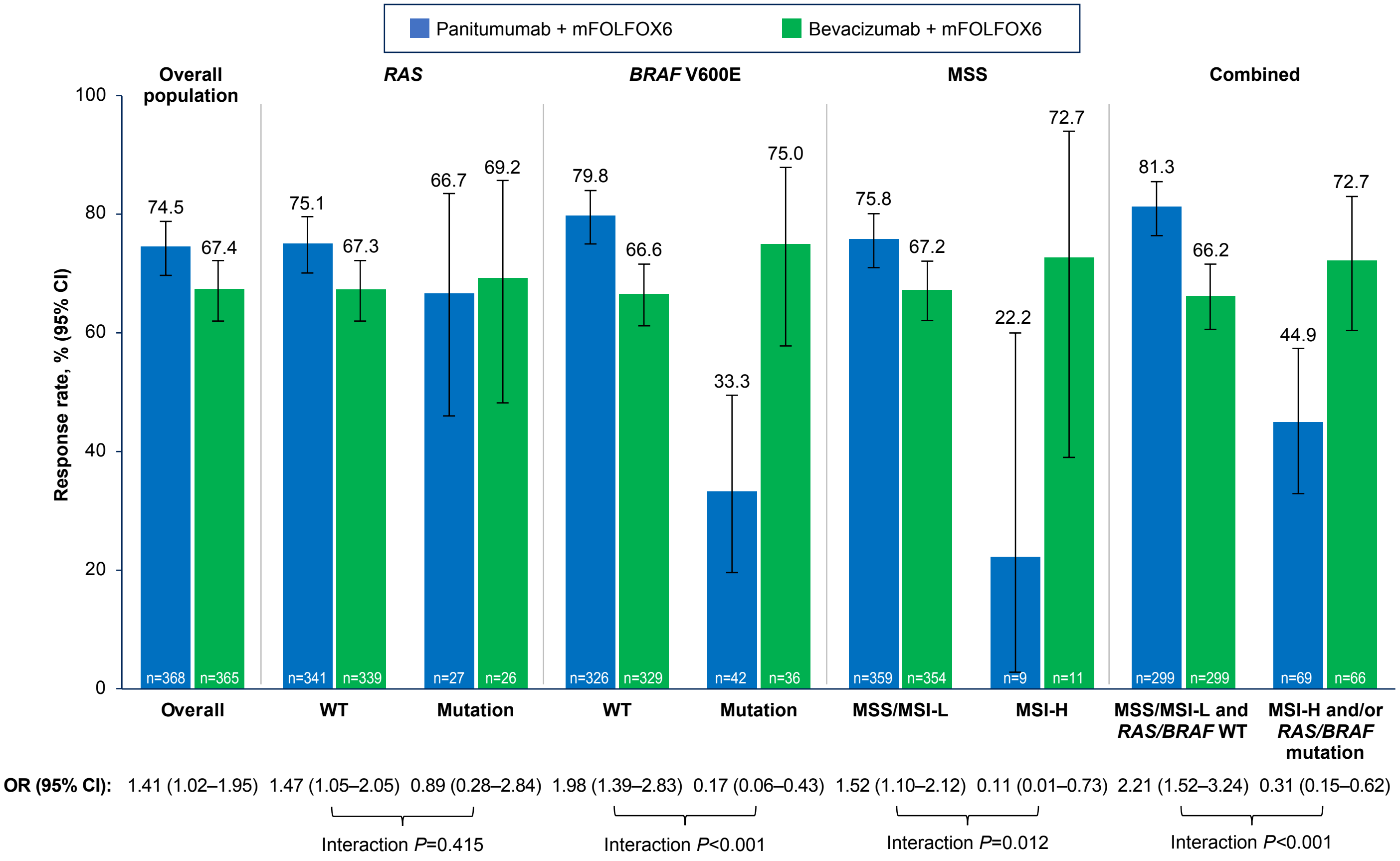

Protocol

An exploratory study of treatment sensitivity and prognostic factors in a phase 3, randomized, controlled study comparing the efficacy and safety of mFOLFOX6 + bevacizumab therapy versus mFOLFOX6 + panitumumab therapy in patients with chemotherapy-naïve, *RAS* (*KRAS/NRAS*) wild-type, unresectable, advanced/recurrent colorectal cancer

Exploratory Analysis of Biomarkers in the **PARADIGM** Study

(Exploratory analysis of predictive and prognostic biomarkers in the PARADIGM Study)

**Sponsor**	Takeda Pharmaceutical Company Limited
**Protocol number**	Panitumumab-4004
**Version**	Version 3
**Product name**	Panitumumab
**Creation date**	June 18, 2018

CONFIDENTIAL

CONFIDENTIAL PROPERTY

This document is confidential information of Takeda Pharmaceutical Company Limited. Acceptance of this document constitutes the agreement by the recipient that no information contained herein will be published or disclosed without written authorization from Takeda except for the occasions necessary for the recipient to obtain informed consent from patients to whom the drug is administered. This document is prepared solely for the recipients, staff, and Institutional Review Board, etc. to evaluate and review the contents and to conduct the industry-sponsored clinical study (hereinafter referred to as "study").

1.0 STUDY PRINCIPLES AND STUDY MANAGEMENT INFORMATION

1.1 Study principles

Out of consideration for individual participants, this study will be conducted in compliance with this study protocol and the following requirements:

- Ethical principles based on the Declaration of Helsinki
- "Ethical Guidelines on Medical Research Involving Human Subjects" (Ministry of Health, Labour and Welfare, December 22, 2014)
- Good Clinical Practice (hereinafter referred to as ICH-GCP)
- All applicable laws and regulations (including data privacy laws and conflict of interest guidelines and rules)

1.2 Study administrative structure

This study will be conducted with the following administrative structure.

Study Steering Committee

Study Steering Committee Chairpersons (General Managers):

Hiroyuki Uetake, Medical Hospital, Tokyo Medical and Dental University

Katsuya Tsuchihara, National Cancer Center

Study Steering Committee Members:

Takeshi Kato, National Hospital Organization Osaka National Hospital

Yoshito Komatsu, Hokkaido University Hospital

Eiji Oki, Kyushu University Hospital

Takeo Sato, Kitasato University Hospital

Takeshi Naito, Tohoku University Hospital

Kei Muro, Aichi Cancer Center Hospital

Takayuki Yoshino, National Cancer Center Hospital East

Takeharu Yamanaka, Yokohama City University

Kentaro Yamazaki, Shizuoka Cancer Center

Kouhei Shitara, National Cancer Center Hospital East

Pathology Specialist

Atsushi Ochiai, National Cancer Center

Clinical Genetics Specialist

Kiwamu Akagi, Saitama Cancer Center

Terms in the study protocol are defined as follows:

Study site:

A study site is a corporation, an administrative body, or a sole proprietor that conducts the study and it excludes those that are contracted to perform only a part of study-related duties, such as storage of samples and information, statistical processing, etc.

Collaborating study site:

A collaborating study site is a study site jointly performing the clinical study according to the study protocol, and it includes institutions that obtain new samples and information for the study from patients and provide other institutions with the samples and information.

Researchers:

Researchers are the investigator and other personnel involved in the conduct of the study (including the conduct of duties at an institution that collects and issues samples and information) and exclude those who only provide existing samples and information outside the study site and those who are contracted to perform only a part of the study-related duties.

Investigator:

An investigator is a person involved in the conduct of the study who exercises control over the study-related duties at the study site to which he/she belongs.

Head of the study site:

The head of the study site is a representative of a corporation, head of an administrative body, or a sole proprietor who conducts the study.

Participants:

Participants are persons who fall under either of the following categories (including the dead):

1. Persons who participate in the study (including those who wish to participate in the study)
2. Persons from whom existing sample/information has been obtained to be used in the study

1.3 Contact information regarding the protocol

Study office: Linical Co, Ltd

Email: protocol@paradigm-study.jp

TEL: 03-6215-8005

Reception hours: Monday to Friday, 9:00–18:00 (excluding Saturday, Sunday, public holidays, and Dec 29–Jan 4)

1.4 Study website

http://www.paradigm-study.jp/

1.5 Sponsor

Takeda Pharmaceutical Company Limited

(Medical Research, Global Medical Affairs–Japan Department, Japan Oncology Business Unit)

TABLE OF CONTENTS

1.0 STUDY PRINCIPLES AND STUDY MANAGEMENT INFORMATION........................3
1.1 Study principles 3
1.2 Study administrative structure 3
1.3 Contact information regarding the protocol 4
1.4 Study website 5
1.5 Sponsor 5
2.0 STUDY SUMMARY 9
3.0 LIST OF ABBREVIATIONS 12
4.0 INTRODUCTION 13
4.1 Background 13
4.2 Rationale for planning the additional study 13
5.0 OBJECTIVES AND ENDPOINTS OF THE STUDY 16
5.1 Objectives 16
5.2 Planned endpoints 16
5.2.1 Primary endpoint 16
5.2.2 Secondary endpoints 16
5.2.3 Exploratory endpoints 16
5.3 Rationale for selection of the endpoints 17
5.3.1 Primary endpoint 17
5.3.2 Secondary endpoints 17
5.3.3 Exploratory endpoints 17
6.0 STUDY DESIGN AND MEASUREMENT ITEMS 18
6.1 Study design 18
6.2 Participants 18
6.3 Measurement items 18
6.4 Rationale for selection of the measurement items 19
6.5 Planned sample size 20
6.6 Discontinuation of the entire study or of the study at a study site 20
6.6.1 Discontinuation of the entire study 20
6.6.2 Criterion for discontinuation of protocol treatment at a study site 20
6.6.3 Procedures of study suspension and discontinuation of entire study or study at a study site 20

6.7 Procedures for revision of the protocol 21
7.0 INCLUSION/EXCLUSION CRITERIA FOR PARTICIPANTS 22
7.1 Inclusion criterion 22
7.2 Exclusion criterion 22
8.0 PROTOCOL, ENDPOINTS, AND OBSERVATION PROCEDURE 23
8.1 Procedures during the study period 23
8.1.1 Acquisition of informed consent 24
8.1.2 Patient background characteristics and laboratory tests 24
8.1.3 Tumor samples 25
8.1.4 Blood samples 25
9.0 MEASUREMENT OF SAMPLE 27
9.1 Storage and disposal of samples 27
10.0 COMMITTEES ESTABLISHED FOR THE ADDITIONAL STUDY 28
10.1 Study Steering Committee 28
10.2 Pathology specialist 28
10.3 Clinical genetics specialist 28
11.0 RETENTION OF RECORDS 29
11.1 Case report form 29
11.2 Storage of records 29
12.0 STATISTICAL PROCEDURES 30
12.1 Statistical analysis plan 30
12.1.1 Analysis population 30
12.1.2 Analyses of demographic and other baseline characteristics 30
12.1.3 Analysis of the primary endpoint 30
12.2 Interim analysis and criteria for premature termination 30
12.3 Determination of the planned sample size 30
13.0 QUALITY CONTROL AND QUALITY ASSURANCE 31
13.1 Monitoring of the study sites 31
13.1.1 Central monitoring 31
13.1.2 Site visit monitoring 31
13.2 Deviation from Ethical Guidelines on Medical Research Involving Human Subjects and study protocol 31
13.3 Quality assurance and investigation by the regulatory authority 32

14.0 ETHICAL CONDUCT OF THE STUDY 33
14.1 Conflict of interest 33
14.2 Approval of committees at the study site such as the IRB 33
14.3 Informed consent document and consent of patients 33
14.4 Patient confidentiality 35
14.5 Consultation services for patients and persons involved 35
14.6 Advantages and disadvantages to participants 36
14.6.1 Advantages to participants 36
14.6.2 Disadvantages to participants 36
14.6.3 Disclosure of test results 36
14.7 Guidelines for publication, disclosure, and study registration 36
14.7.1 Report, publication, and disclosure of test results 36
14.7.2 Clinical study registration 37
14.7.3 Disclosure of study results 37
14.8 Attribution of study results and intellectual property right 37
14.9 Insurance/compensation for damages 37
15.0 REFERENCES 38
APPENDIX A RESPONSIBILITIES OF THE INVESTIGATOR 39

LIST OF IN-TEXT FIGURES AND TABLES

Figure 6.a Outline of study design 18
Figure 8.a When informed consent for this additional study was obtained from patients before initiation of protocol treatment of the main study 23
Figure 8.b When informed consent for this additional study was obtained from patients after the initiation of the protocol treatment of the main study 24
Table 9.a Gene testing methods and laboratories 27

2.0 STUDY SUMMARY

**Sponsor:** Takeda Pharmaceutical Company Limited
**Study drug:** Panitumumab
**Study title:** An exploratory study of treatment sensitivity and prognostic factors in a phase 3, randomized, controlled study comparing the efficacy and safety of mFOLFOX6 + bevacizumab therapy versus mFOLFOX6 + panitumumab therapy in patients with chemotherapy-naïve, *RAS* (*KRAS/NRAS*) wild-type, unresectable, advanced/recurrent colorectal cancer
**Protocol number:** Panitumumab-4004
**Type of study:** Exploratory study
**Clinical study design:** 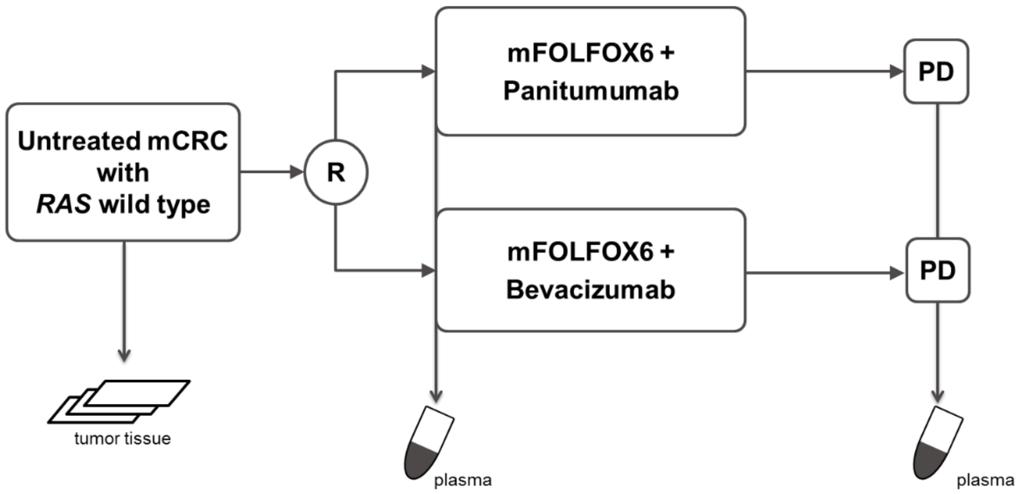 PD, progressive disease; R, registration/randomization; Untreated mCRC with *RAS* wild-type, chemotherapy-naïve patients with *RAS* wild-type, unresectable, advanced/recurrent colorectal cancer.
**Objectives:**<br>The objectives of this study are to investigate biomarkers that may be predictors of efficacy, to perform exploratory research on biomarkers for patient stratification, to examine the relationship between levels of biomarkers in plasma free DNA and in tumor tissue, to observe differences in ctDNA mutations before and after protocol treatment, and to confirm the usefulness of ctDNA as a less-invasive biomarker analysis platform for patients with metastatic CRC. This is an additional study of a phase 3, randomized, controlled study comparing the efficacy and safety of mFOLFOX6 + bevacizumab therapy versus mFOLFOX6 + panitumumab therapy in patients with chemotherapy-naïve, *RAS*(*KRAS*/*NRAS*) wild-type, unresectable, advanced/recurrent colorectal cancer (PARADIGM Study; Protocol No.: Panitumumab-3001; hereinafter referred to as the “main study”).
**Participants:**<br>Of the 800 patients who are enrolled in the main study (PARADIGM Study)—at sites where approval of the head of the study site is obtained based on review and approval of the Institutional Review Board (IRB), etc, for the protocol for the additional study including provision of samples to external parties—the participants in this additional study will be those who provided consent for this additional study.

Planned sample size:

Of the patients who are enrolled in the main study, those who provided written consent for the additional study will be accumulated to the extent possible. The sample size of the main study is as follows:

mFOLFOX6 + panitumumab combination therapy: 400 patients

mFOLFOX6 + bevacizumab combination therapy: 400 patients

Number of study sites:

Of the study sites that participated in the main study, those for which the approval of the head of study site was obtained, on the basis of review by the IRB, for conduct of the additional study (approximately 200 medical institutions at maximum)

Inclusion criterion:

Patients who are enrolled in the main study and who personally provide written consent after the contents of the additional study are adequately explained to them

Exclusion criterion:

Patients who are determined by the investigator or researchers to be not suitable for participation in the additional study

Endpoints:

Primary endpoint:

Evaluation of the relationship between OS in the main study and mutation of each gene (eg, *BRAF*, *PIK3CA*, *EGFR*, *KRAS*,* and *NRAS**) in tumor samples from the baseline of the main study

*: Unified testing of *KRAS* and *NRAS* mutations will be carried out separately from assessments used for determining eligibility for the main study

Secondary endpoints:

Evaluation of the relationship between other endpoints of efficacy obtained in the main study and each tumor-associated gene in tumor samples from the baseline of the main study. The other endpoints of efficacy include the following:

1. PFS
2. Response rate
3. Duration of response
4. Proportion of patients who proceeded to surgical resection
5. Proportion of patients with early tumor shrinkage
6. Degree of maximum tumor shrinkage (depth of response)

Exploratory endpoints:

1. Evaluation of the relationship between tumor samples collected from the baseline of the protocol treatment of the main study and each biomarker in plasma free DNA.
2. Evaluation of the relationship between endpoints of efficacy and each biomarker in plasma free DNA from the baseline of the protocol treatment of the main study
3. Evaluation of the relationship between efficacy and changes in each biomarker in plasma free DNA from baseline to the discontinuation of the protocol treatment of the main study
4. Evaluation of the relationship between efficacy and changes in each biomarker in the whole tumor from baseline to the discontinuation of the protocol treatment of the main study

**Statistical procedures:**<br>By presence or absence of each gene mutation in tumor tissue, Kaplan-Meier survival curves of time to event will be shown by treatment group. A quartile point of survival by treatment group and point estimates with two-sided 95% confidence intervals for survival rates at certain time points will be calculated.
**Rationale for determination of the planned sample size:**<br>Given that this is an additional study of the main study, the planned sample size will be within the number of patients who are enrolled in the main study. Because this is an exploratory study, there is no rationale for determination.
**Period of the study:**<br>Total period of the study: April 2015* to June 2020 (63 months)<br>Enrollment period: April 2015* to June 2017 (27 months)<br>Follow-up period: 36 months after the end of enrollment<br>*: Registration will be started after *KRAS/NRAS* testing obtains marketing and reimbursement approval and *KRAS/NRAS* measurement becomes available at each study site.

3.0 LIST OF ABBREVIATIONS

Abbreviation	Full expression	Japanese
BRAF	v-raf murine sarcoma viral oncogene homolog B1	*BRAF*
COI	conflict of interest	利益相反
CRC	colorectal cancer	
DNA	deoxyribonucleic acid	デオキシリボ核酸
EGFR	epidermal growth factor receptor	上皮成長因子受容体
IC	informed consent	
ICH-GCP	International Conference on Harmonisation–Good Clinical Practice	
ID	identification	
IRB	Institutional Review Board	
JAPIC	Japan Pharmaceutical Information Center	
KRAS	Kirsten rat sarcoma-2 virus	Kristen ラット肉腫-2 ウイルス
mFOLFOX	modified chemotherapy regimen including fluorouracil, levofolinate calcium, and oxaliplatin	
NRAS	neuroblastoma rat sarcoma	*NRAS*
OS	overall survival	全生存期間
PFS	progression-free survival	無増悪生存期間
PI3K	phosphoinositide 3-kinase	イノシトールリン脂質 3 リン酸化酵素
PIK3CA	phosphoinositide 3-kinase, catalytic, alpha polypeptide	ホスファチジルイノシトール-4,5-二リン酸 3-キナーゼ触媒サブユニット α ポリペプチド
RAS	rat sarcoma	ラット肉腫
VEGF	vascular endothelial growth factor	血管内皮細胞増殖因子

4.0 INTRODUCTION

4.1 Background

Molecular-targeted agents targeting intracellular signaling, such as the vascular endothelial growth factor (VEGF) pathway and the epidermal growth factor receptor (EGFR) pathway, have been developed as a therapeutic strategy for unresectable, advanced/recurrent CRC. These agents include bevacizumab, an anti-VEGF antibody drug, and cetuximab and panitumumab, anti-EGFR antibody drugs. *KRAS* mutations have been investigated as a biomarker for the efficacy of anti-EGFR antibody drugs. The results of a retrospective analysis of some pivotal studies, such as the PEAK Study,[1] FIRE-3 Study,[2] and CALGB/SWOG80405 Study[3] showed that patients with *KRAS* and *NRAS* mutations received no therapeutic benefit from anti-EGFR antibody drugs. To date, no useful biomarker has been identified for bevacizumab, an anti-VEGF antibody drug.

4.2 Rationale for planning the additional study

Panitumumab, an anti-EGFR antibody, was used in a phase 3, randomized, controlled study to investigate the efficacy and safety of mFOLFOX6 + bevacizumab combination therapy versus mFOLFOX6 + panitumumab combination therapy in patients with chemotherapy-naïve, *RAS* (*KRAS/NRAS*) wild-type, unresectable, advanced/recurrent CRC (PARADIGM Study; study protocol number: Panitumumab-3001; hereinafter referred to as “the main study”). Panitumumab was also used in a phase 3 clinical study (PRIME Study) in which FOLFOX4 monotherapy was compared with FOLFOX4 therapy + panitumumab (given at a dose of 6 mg/kg every 2 weeks) in two arms as a first-line treatment of unresectable, advanced/recurrent CRC. In the PRIME Study, the median PFS in patients with *KRAS* wild type was 9.6 months in the FOLFOX4 + panitumumab arm, which is significantly longer than the 8.0 months in the FOLFOX4 alone arm (hazard ratio, 0.80; p=0.02).[4] In contrast, the efficacy of panitumumab combination therapy was not shown in patients with *KRAS* mutations in exon 2; *KRAS* mutations in exon 2 were found to be a predictor of treatment failure for anti-EGFR antibody drugs.

In a phase 3, randomized, controlled study (20040408) to demonstrate the efficacy of panitumumab monotherapy as third-line treatment after standard therapy in patients with unresectable, advanced/recurrent CRC, an exploratory analysis of biomarkers was conducted. It was revealed that the presence or absence of not only *KRAS* exon 2 mutations but also the *KRAS* exon 3 or 4 or *NRAS* exon 2, 3, or 4 mutations was likely to predict the efficacy of panitumumab.[5]

In a phase 2 study (PEAK Study) of mFOLFOX6 + panitumumab versus mFOLFOX6 + bevacizumab as a first-line therapy in patients with unresectable, advanced/recurrent CRC, PFS and OS were analyzed in patients with *KRAS* exon 2, 3, or 4 and *NRAS* exon 2, 3, or 4 mutations as the *RAS (KRAS/NRAS)* mutations (hereinafter referred to as *RAS* genes) and in patients without these mutations (wild-type *RAS*). In the patients with wild-type *RAS*, the OS was significantly prolonged. There was no significant difference in PFS.[1]

Among the patients who were enrolled in the PRIME Study and were determined to have wild-type *KRAS* exon 2 (by the Therascreen K-RAS MUtation Kit [Roche]), the relationships between the presence or absence of *KRAS* exon 3 (codon 61) or 4 (codon 117 or 146); *NRAS* exon 2 (codon 12 or 13), 3 (codon 61), or 4 (codon 117 or 146); or *BRAF* exon 15 (codon 600) mutations in tumors (by the Sanger method of DNA sequencing and WAVE-based SURVEYOR Scan Kit [Transgenomic]) and PFS or OS were reported.[6]

The results of the PRIME Study suggested that, for first-line treatment in patients with the wild-type *RAS*, excluding not only *KRAS* exon 2 in codon 12 or 13 but also other less frequent *KRAS* and *NRAS* mutations, mFOLFOX + panitumumab therapy might improve PFS and OS more than mFOLFOX monotherapy. In contrast, for wild-type *KRAS* exon 2 in patients with either *KRAS* or *NRAS* mutations,

there was no improvement in PFS or OS. Therefore, mutations other than *KRAS* exon 2 were assumed to be predictors of treatment failure.

Therefore, the participants in the main study (PARADIGM Study) were selected to be patients with unresectable, advanced/recurrent CRC without mutations in any of *KRAS* or *NRAS* exon 2 (codon 12 or 13), exon 3 (codon 59 or 61), and exon 4 (codon 117 or 146), for which the maximum effect of panitumumab can be exerted at present. In addition to *RAS* mutations, *BRAF* and *PIK3CA* mutations, *PTEN* deletion and *AKT* expression have been reported as factors likely to be associated with treatment failure of anti-EGFR antibody treatment.[7] Furthermore, the amplification of *ERBB2 (HER2)*, *KRAS*, and *MET* genes has been reported to be a mechanism of acquiring resistance to anti-EGFR antibody treatment. In addition, administration of cetuximab, an anti-EGFR antibody drug, has been reported to cause the S492R EGFR ectodomain mutation that affects the binding property of cetuximab[8] and has been considered to be a factor for acquiring resistance to cetuximab.[9-11]

The PRIME Study results showed that, in patients with neither *RAS* nor *BRAF* mutations, mFOLFOX + panitumumab therapy improved PFS and OS more than mFOLFOX monotherapy, but when *BRAF* mutations were present, the degree of improvement decreased.[6]

PIK3CA mutations have been known to be a possible biomarker for predicting the efficacy of aspirin as adjuvant therapy in CRC.[11] *PIK3CA* mutations are frequent in patients with *KRAS* mutations; thus, *PIK3CA* mutations have been reported to be a poor prognostic factor, but no certain determination as to whether or not they can be of a predictor of the efficacy of anti-EGFR antibody drugs has been made at present.

Post hoc analyses[12,13] of the PRIME and 20050181 studies of panitumumab showed the effect of panitumumab on EGFR expression regardless of its degree.

Thus, gene analysis of tumor tissues appears to be very useful for treatment prediction and evaluation of resistance acquisition. However, it has been pointed out that there are issues to overcome: evaluation of biopsy samples from a single site cannot be used to observe the gene mutation of the whole tumor because of the heterogeneity of the tumor itself; biopsies cannot be performed frequently because frequent biopsies are invasive and can accelerate temporal gene evolution of the cancer; and evaluation of resistance acquisition is difficult.

Recently, it has been reported that DNA derived from tumor cells (cell-free DNA or circulating free DNA) is shed into the bloodstream from dead cells by apoptosis, etc, in the tumor tissues and it is possible to measure this tumor cell–derived DNA in blood samples. This analysis is less invasive than direct sampling of tumor tissues and makes real-time monitoring of each tumor possible. Cell-free DNA is considered to have been detached from comparatively random sites on the primary lesion and metastatic lesions, and therefore it can overcome inhomogeneity. Because the analysis can be performed on blood samples, it is also expected that less invasive measurement of gene mutation that would cause resistance acquisition may be possible.

After treatment with anti-EGFR antibody drugs in patients with wild-type *KRAS* CRC, *KRAS* mutations have been reported to be detected from tumor cell–derived DNA in blood samples.[14,15] In addition, in CRC patients who were enrolled in the CORRECT Study, which was a phase 3 study of regorafenib, *KRAS* mutations in tumor tissues and plasma free DNA before treatment with regorafenib were examined using the BEAMing method, a type of digital PCR. The results revealed a concordance rate of *KRAS* mutations of 76%, and the majority of discordant *KRAS* mutations were reported to be the wild type in tumor tissues and the mutant type in blood samples (plasma).[16] These results suggest that occurrence of mutant genes in patients with wild-type *KRAS* appears to be caused by proliferation of *KRAS* gene mutant clones during administration of anti-EGFR antibodies and draw attention to this resistance mechanism against anti-EGFR antibodies.

Furthermore, a procedure to use a next-generation sequencer for cell-free DNA analysis has been developed, and not only analyses of gene mutations but also the copy number of *KRAS*, *MET*, *ERBB2*, and *EGFR* have been reported in patients with CRC treated with anti-EGFR antibody.[17] However, the above-mentioned studies all investigated technical possibilities in a small portion of patients, and these

investigations are inadequate to prove that cell-free DNA in blood is actually helpful as a biomarker. This study uses a controlled, prospective population, and, therefore, it is considered that a higher quality investigation may be possible.

In recent years, in addition to those mentioned above, a number of infrequent mutations, copy number abnormalities, and gene rearrangements have been reported as a result of genetic analysis of CRC with a next-generation sequencer in Europe and the United States, presented by Cancer Genome Atlas. However, how these abnormalities are related to prognosis and treatment prediction is not yet known, and there is a possibility that new biomarkers may be identified in the future.

Gene mutations have not been reported as a treatment predictor for anti-VEGF antibody. Nonetheless, a relatively large sample size is planned, and this additional study to introduce a new measurement technique may well be able to identify a new biomarker.

Furthermore, in Japan, where no large-scale data of genome analysis of CRC exists, as it does in Europe and the United States, the data on Japanese patients that can be obtained from this study is considered very significant.

Testing for cell-free DNA in blood to (1) investigate overcoming the heterogeneity of tumors, (2) perform individualized molecular monitoring of tumor (because of the short half-life), and (3) observe gain-of-function mutation (resistance acquisition) is assumed to be helpful for developing an analysis of prognosis with higher accuracy and a less invasive and temporal analysis of treatment prediction for molecular biological agents such as EGFR and VEGF antibody in patients with CRC.

On the basis of the above findings, we planned this additional study to investigate biomarkers of panitumumab and bevacizumab other than *KRAS* and *NRAS* to be able to conduct more detailed case selection from genetic mutation status of tumors and to use plasma free DNA to understand the status of CRC immediately before initiation of treatment or at discontinuation of the protocol treatment to contribute to improving outcomes of CRC.

5.0 OBJECTIVES AND ENDPOINTS OF THE STUDY

5.1 Objectives

The objectives of this study are to investigate biomarkers that may be predictors of efficacy, to perform exploratory research on biomarkers for patient stratification, to examine the relationship between levels of biomarkers in plasma free DNA and in tumor tissue, to observe differences in ctDNA mutations before and after protocol treatment, and to confirm the usefulness of ctDNA as a less-invasive biomarker analysis platform for patients with metastatic CRC.

5.2 Planned endpoints

5.2.1 Primary endpoint

Evaluation of the relationship between OS and mutation of each gene (eg, *BRAF*, *PIK3CA*, *EGFR*, *KRAS*,* and *NRAS**) in tumor samples from the baseline of the main study.

*: Unified testing of *KRAS* and *NRAS* mutations will be carried out separately from the central assessments used for determining eligibility for the main study.

5.2.2 Secondary endpoints

Evaluation of the relationships between other efficacy endpoints of the main study and each tumor-associated gene in tumor samples from the baseline of the main study.

1. PFS
2. Response rate
3. Duration of response
4. Proportion of patients who proceed to surgical resection
5. Proportion of patients with early tumor shrinkage
6. Degree of maximum tumor shrinkage (depth of response)

5.2.3 Exploratory endpoints

1. Evaluation of the relationship between tumor samples collected from baseline of the protocol treatment of the main study and each biomarker in plasma free DNA
2. Evaluation of the relationship between endpoints of efficacy and each biomarker in plasma free DNA from the baseline of the protocol treatment in the main study
3. Evaluation of the relationship between efficacy and changes in each biomarker in plasma free DNA from baseline to the discontinuation of the protocol treatment in the main study
4. Evaluation of the relationship between efficacy and changes in each biomarker in the whole tumor from baseline to the discontinuation of the protocol treatment in the main study

5.3 Rationale for selection of the endpoints

5.3.1 Primary endpoint

The primary endpoint has been selected for evaluating whether each gene mutation in tumor tissue can be of a predictor of OS, which is the primary endpoint in the main study.

5.3.2 Secondary endpoints

The secondary endpoints have been chosen to assess whether each gene mutation in tumor tissue can be a biomarker for measures of efficacy that are specified to be the secondary endpoints in the main study.

5.3.3 Exploratory endpoints

The exploratory endpoints have been selected to evaluate whether a correlation of each biomarker in tumor tissue and plasma free DNA enables the detection of gene mutations in tumor tissue. The correlation between change in each biomarker in plasma free DNA from baseline to the discontinuation of the protocol treatment in the main study and efficacy has been chosen to reveal the mechanism of resistance to anti-EGFR antibody drugs and to examine whether or not findings contributing to the future of individualized medicine can be obtained.

For patients from whom a tumor sample was obtained during discontinuation of the protocol treatment (from 28 days after discontinuation of the protocol treatment to immediately before the start of the next treatment), whether change in each biomarker is observed before and after the treatment in plasma free DNA and in the tumor sample will be investigated.

6.0 STUDY DESIGN AND MEASUREMENT ITEMS

6.1 Study design

In this additional study of tumor tissues sampled during surgery or by endoscopy from patients who are enrolled in the main study (PARADIGM Study) and provide consent for the additional study, mutations, amplification, and rearrangement of predefined tumor-associated genes will be investigated with DNA from tumor samples collected for assessing *RAS* mutations and plasma free DNA collected before administration of cycle 1 and at the discontinuation of the protocol treatment in the main study.

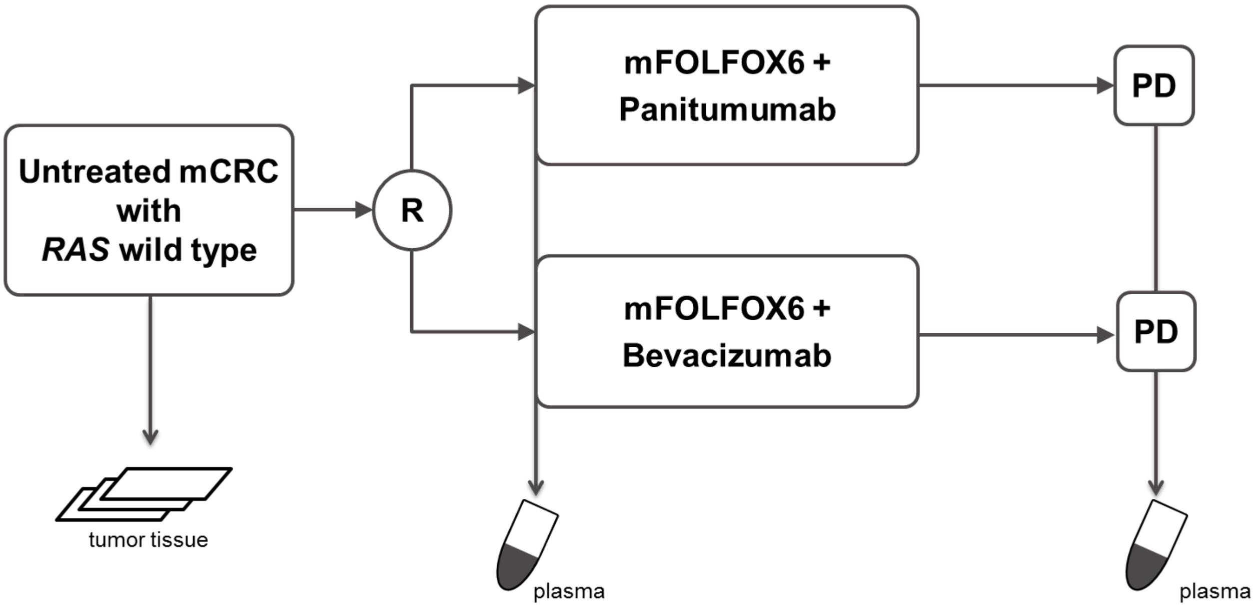

Figure 6.a Outline of study design

6.2 Participants

Of the 800 patients who are enrolled in the main study (PARADIGM Study), at sites where approval of the heads of the study sites is obtained based on review and approval of the IRB for the protocol for the additional study, including provision of samples to external parties, the participants in this additional study will be those who provided consent for this additional study.

6.3 Measurement items

Investigation of genetic mutation, amplification, and rearrangement of tumor-associated genes such as *BRAF*, *PIK3CA*, *EGFR*, *KRAS*, and *NRAS*, which are associated with protocol treatment of the main study. See Attachment 3 for the tumor-associated genes selected for the investigation.

6.4 Rationale for selection of the measurement items

Rationales for the selection of the planned main biomarkers are described below. However, new findings on biomarkers and measurement techniques for molecular-targeted agents are becoming available every day. Thus, there is a possibility that new measurement items and tests involving new measurement methods may be added as a result of findings obtained while the additional study is being conducted. Hence, tumor-associated genes including, but not limited to, those set forth below may be evaluated.

1. *BRAF*
   BRAF is serine-threonine kinase composing the RAS/MAPK pathway. *BRAF* mutations are identified in 5% to 15% of patients with CRC, and 90% of those mutations involve glutamate being substituted for valine at codon 600 (V600E). *KRAS* mutations are almost never identified in patients with CRC with *BRAF* mutations; thus, *KRAS* and *BRAF* mutations are in a mutually exclusive relationship. In patients with metastatic CRC with *BRAF* mutations, the efficacy of anti-EGFR antibody drugs has not been clearly demonstrated;[18,19] but, because the incidence of *BRAF* mutations is low, the evaluation of *BRAF* as a prognostic factor is difficult. In addition, an analysis in the PRIME Study revealed that mFOLFOX + panitumumab therapy improved PFS and OS more than mFOLFOX monotherapy did in patients with neither *RAS* nor *BRAF* mutations; but, when *BRAF* mutations were present, the degree of improvement decreased.[6] Consequently, it is not yet clear whether anti-EGFR antibody drugs are effective for *BRAF* mutation CRC; it will be meaningful to further evaluate this relationship in this additional study. In addition, it is not yet known whether BRAF mutations are a prognostic factor for bevacizumab, and this relationship will be examined in this additional study.

2. *PIK3CA*
   PI3K is lipid kinase that catalyzes the phosphorylation of PIP2 to form PIP3. PIP3 is transmitted as a proliferation signal via downstream PDK1 or AKT. In CRC, the *PIK3CA* gene plays a more important role than *PI3K*. *PIK3CA* mutations are noted in approximately 15% of patients with CRC, and mutations in exon 9 and exon 20 account for the majority of those mutations. *PIK3CA* mutation CRC is reported to be frequent in patients with *KRAS* mutation CRC and a poor prognostic factor,[20] but it is also reported to have no correlation with prognosis.[21]
   Consequently, no certain determination has been made as to whether the presence or absence of *PIK3CA* mutations can be an effective predictor of anti-EGFR antibody drugs; thus, it is considered to be meaningful to further evaluate it in this additional study. In addition, no certain view on the relationship between bevacizumab and *PIK3CA* mutations has been obtained, but their potential for being a prognostic factor will be examined.

3. *EGFR*
   The results of an in-vitro study show that long-term cetuximab exposure in cetuximab-sensitive human CRC cells causes cetuximab-resistant *EGRF* S492R mutations.[22]
   Also, in patients with wild-type *KRAS*, advanced/recurrent CRC resistant to chemotherapy combined with anti-EGFR antibody drugs, mutations of *EGFR* genes (mainly S492R mutation), which had not been noted in pretreatment tumor tissue, were detected in plasma in 7% of the patients.[8] These mutations have been assumed to occur at the sites of cetuximab binding to EGFR, thereby decreasing the binding property of cetuximab. No similar report has been made for panitumumab, but an analysis of *EGFR* mutations is important because *EGFR* mutations are an expected mechanism of resistance to panitumumab treatment.

4. *KRAS*
   KRAS is a low molecular weight, guanosine triphosphate–binding protein that plays a role in transmitting signals downstream from EGFR. *KRAS* mutations are identified in 35% to 40% of patients with CRC, and the majority of the mutations are in exon 2 at codon 12 (approximately 80%) and codon 13 (approximately 20%). In the PRIME Study, the efficacy of panitumumab combination therapy was not shown in patients with *KRAS* mutations in exon 2; thus, *KRAS* mutations in exon 2 were found to be a predictor of treatment failure for anti-EGFR antibody drugs.[4] Furthermore, an analysis in the PRIME Study suggested that mFOLFOX + panitumumab therapy would not be effective in patients without *KRAS* exon 2 mutations but with other less

frequent *KRAS* mutations.[6]
In this additional study, patients with wild-type *KRAS* exon 2 (codons 12, 13), exon 3 (codons 59, 61), and exon 4 (codons 117, 146) will be selected. Unified testing will also be performed to assess concordance between the commercial test kit and the research test kit. (If *KRAS* mutations are identified at this stage, a relationship with efficacy indices will be also examined.)

5. *NRAS*
NRAS mutations are noted in 3.5% of patients with CRC. In Study 20040408 investigating the efficacy of panitumumab monotherapy as third-line treatment, an analysis of exploratory biomarkers was performed and showed that the presence or absence of *NRAS* exon 2, 3, and 4 mutations might be a predictor of the efficacy of panitumumab as well.[5] Anti-EGFR antibody drugs may not have good merits for patients with *NRAS* mutations.
In this additional study, patients with wild-type *NRAS* exon 2 (codons 12, 13), exon 3 (codons 59, 61), and exon 4 (codons 117, 146) will be selected, but unified testing will be separately performed to add an examination on concordance of assessments. (If *NRAS* mutations are identified at this stage, a relationship with efficacy indices will be also examined.)

6.5 Planned sample size

The number of patients in this additional study will remain within the number of patients registered in the main study (800 patients as a planned sample size). See Section 12.3.

6.6 Discontinuation of the entire study or of the study at a study site

6.6.1 Discontinuation of the entire study

When the premature termination of the main study is decided, the collection of new samples (tumor and blood samples) in the additional study should be discontinued.

6.6.2 Criterion for discontinuation of protocol treatment at a study site

If a serious violation of Ethical Guidelines on Medical Research Involving Human Subjects, ICH-GCP, study protocol, or contract by a study site (including the investigator) is found; or conduct of a proper clinical study is impossible; or discontinuation is recognized by contractual agreements, then the sponsor may demand that a study site discontinue the study.

6.6.3 Procedures of study suspension and discontinuation of entire study or study at a study site

When the sponsor or committees at the study sites, such as the IRB, decide to interrupt or prematurely terminate the entire additional study or the additional study at a study site, the sponsor will instruct

procedures specified for the additional study. When interrupting or discontinuing the study, the concerned study site should follow the procedure.

6.7 Procedures for revision of the protocol

When a need arises to revise the protocol, the sponsor and the Study Steering Committee chairpersons will discuss the issue to make a decision.

The study protocol is to be revised only when a matter falls under the following items. When revised, details of the protocol revision will be notified to investigators at all study sites. When the investigator of each study site receives the notification, he/she will confirm the revision and submit an agreement to the sponsor as evidence of agreement to the revision of the study protocol.

The study protocol may be revised in the following cases:

1. Change or addition of objectives
2. Addition of test(s) (frequencies/items) or change in the test method that increases burden on the participants
3. Serious change in, or addition to, exclusion/inclusion criteria
4. Change in planned number of patients
5. Change that has been determined to be a serious change in discussion between the sponsor and the Study Steering Committee chairperson

When the investigator of each study site receives the notification, he/she should arrange for the revised protocol to be reviewed by the IRB again, according to the stipulation at each study site, and obtain approval of the head of the study site.

7.0 INCLUSION/EXCLUSION CRITERIA FOR PARTICIPANTS

7.1 Inclusion criterion

The participants in the additional study will be patients who meet the following criterion:

1. Patients who are enrolled in the main study and personally provide written consent after the contents of the additional study are adequately explained to them

7.2 Exclusion criterion

Patients who meet the following criterion will not be included in the additional study:

1. Patients who are determined by the investigator or researchers to be not suitable for participating in the additional study

8.0 PROTOCOL, ENDPOINTS, AND OBSERVATION PROCEDURE

The investigator or researchers should collect tumor and blood samples in accordance with the procedures described below.

8.1 Procedures during the study period

The investigator or researchers should conduct tests of the following items at the time points shown in Figures 8.a or 8.b, depending on when informed consent was obtained from participants in this additional study.

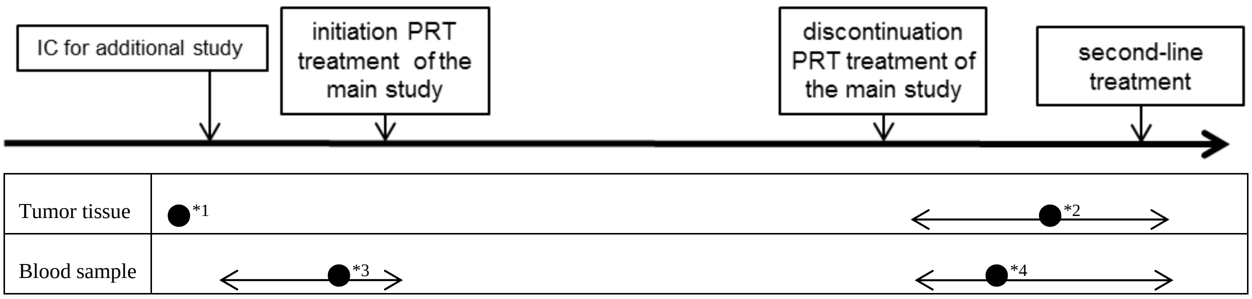

IC, informed consent; PRT, protocol treatment.

*1: Samples collected during surgery or by biopsy before acquisition of informed consent and, in principle, the tumor tissues used for evaluation of *RAS* genes in the main study will be submitted. When the tumor tissues used for evaluation of the *RAS* gene in the main study cannot be submitted, submission of different tumor tissues will be accepted.

*2: When tumor tissues were collected from patients during discontinuation of protocol treatment (from the discontinuation day of protocol treatment to the initiation day of next treatment), and when submission of those tumor tissues is possible, the participant should submit the tumor tissues. Clinically unnecessary intervention (rebiopsy, surgery, etc) to collect a sample during discontinuation of protocol treatment is prohibited.

*3: After acquisition of informed consent from participants in this additional study, blood samples should be collected to initiate the study.

*4: Blood samples should be collected during the discontinuation of the main study protocol (from the discontinuation day of the study protocol to the initiation day of the next treatment).

Figure 8.a When informed consent for this additional study was obtained from patients before initiation of protocol treatment of the main study

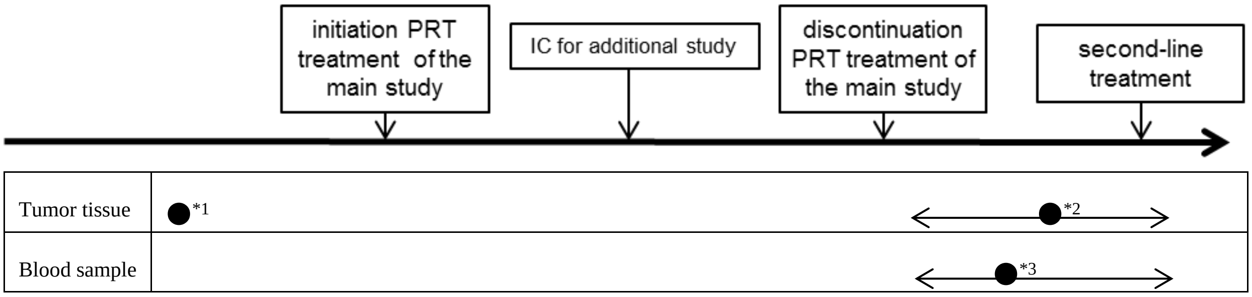

IC, informed consent; PRT, protocol treatment.

*1: Samples collected during surgery or by biopsy before acquisition of informed consent and in principle, the tumor tissues used for evaluation of *RAS* genes in the main study will be submitted. When the tumor tissues used for evaluation of the *RAS* gene in the main study cannot be submitted, submission of different tumor tissues will be accepted.

*2: When tumor tissues were collected from patients during discontinuation of protocol treatment (from the discontinuation day of protocol treatment to the initiation day of next treatment), and when submission of those tumor tissues is possible, the participant should submit the tumor tissues. Clinically unnecessary intervention (rebiopsy, surgery, etc) to collect a sample during discontinuation of protocol treatment is prohibited.

*3: Blood samples should be collected during the discontinuation of the main study protocol (from the discontinuation day of the study protocol to the initiation day of the next treatment).

Figure 8.b When informed consent for this additional study was obtained from patients after the initiation of the protocol treatment of the main study

8.1.1 Acquisition of informed consent

The consent of the patients should be received before undertaking study procedures.

A patient identification (ID) code assigned to each patient at the time of providing an explanation for informed consent will be that assigned to that patient in the main study.

The method for obtaining informed consent is described in Section 14.3.

8.1.2 Patient background characteristics and laboratory tests

Demographic data and laboratory tests collected in the main study will be used in the additional study as well, so no new data entry will be needed for this additional study.

When samples from different sites from those of the *RAS* test are submitted, the following information should be stated in the testing request form.

- Sample collecting sites (primary lesion [rectum/colon] / metastatic lesion)
- Sample used for diagnosis:
  - Biopsy sample
  - Surgical sample
- Date of collecting sample

8.1.3 Tumor samples

8.1.3.1 Test samples

Among tumor samples collected during surgery or by biopsy before acquisition of informed consent of the main study and this additional study, in principle, the tumor tissues used for evaluation of *RAS* gene in the main study will be used. When the tumor tissues used for evaluation of *RAS* gene in the main study cannot be submitted, submission of different tumor tissues will be accepted. As for patients from whom tumor samples were collected during discontinuation of the protocol treatment (from 28 days after discontinuation of the protocol treatment to the initiation of next treatment), samples collected after discontinuation should also be submitted if available. Clinically unnecessary intervention (rebiopsy, surgery, etc) to collect a sample during discontinuation of protocol treatment is prohibited.

8.1.3.2 Submission of tumor samples

The investigator, researchers, or study collaborator will send samples with materials prepared by LSI Corporation. LSI Corporation should send materials necessary for sample collection in advance to the study sites where patients can be enrolled for the additional study.

Tumor samples should be paraffin-embedded specimens sliced to a thickness of 10 μm and mounted on glass slides. Fifteen of these unstained pathological specimens should be submitted to LSI Medience Corporation (hereinafter referred to as “LSI Corporation”). If the number of specimens is less than 15, then as many specimens as possible should be submitted. Paraffin-embedded specimen may also be submitted as it is without being sliced. LSI Corporation will slice the necessary tissue sections from the paraffin-embedded specimen submitted and mount them on glass slides. Paraffin-embedded specimens that were submitted and unused will be returned to the study site as requested. The investigator, researcher, or study collaborator will state the date of collecting the tumor sample for submission on a testing request form.

Details regarding submission of tumor samples should be stated in a procedure prepared separately.

8.1.4 Blood samples

8.1.4.1 Test samples

Blood will be sampled at two time points: before the start of cycle 1 of the protocol treatment in the main study and at the discontinuation of the protocol treatment (from discontinuation day of the protocol treatment to the initiation of the next treatment), from patients who provided informed consent for the additional study before the start of cycle 1 of the protocol treatment in the main study.

Regarding patients from whom informed consent for this additional study was obtained after the start of cycle 1 of the protocol treatment in the main study, blood samples should be collected during discontinuation of the protocol treatment.

8.1.4.2 Collection of blood samples

The investigator, researchers, or study collaborator will collect samples with materials prepared by LSI Corporation. For collection of blood, two 8-mL pre-anticoagulated tubes with EDTA (ethylenediaminetetraacetic acid) should be used.

For blood collection before the start of cycle 1 of the protocol treatment, blood samples should be collected after the acquisition of informed consent for this additional study and up until immediately

before the initiation of the protocol treatment. For blood collection during discontinuation of the protocol treatment, samples should be collected by the initiation of the next treatment.

When separation of plasma is performed at the study site, the blood sample should be centrifuged at 3,000 rpm for 10 minutes and the resulting plasma should be dispensed from the blood-collecting tube after centrifugation and stored in a freezer set at −20°C or lower until LSI Corporation recovers it.

When separation of plasma cannot be performed at the study site, samples should be stored in a refrigerator (that prevents them from being frozen) until LSI Corporation recovers them. Samples should be submitted to LSI Corporation on the same day as blood collection. Sample collection should be performed Monday through Friday. Blood collection should not be performed on a Saturday, a Sunday, or the day before a national holiday because separation of plasma should be performed within 48 hours after the collection of samples.

Information on date and time of blood sample collection should be stated in a testing request form.

8.1.4.3 Submission of blood samples

LSI Corporation should send materials necessary for sample collection and transport beforehand to the study sites where patients can be enrolled in the additional study.

The researchers or study collaborator should be sure to request that LSI Corporation collect samples before collecting blood samples or plasma samples.

Details on the collection and submission of blood samples are presented in a separately issued procedure.

9.0 MEASUREMENT OF SAMPLE

Gene mutation testing will be performed on the DNA samples extracted from the tumor and blood samples.

Table 9.a shows the gene testing methods and laboratories for the tumor and blood samples.

Table 9.a Gene testing methods and laboratories

Sample type	Testing method	Laboratory
Tumor tissue	Next-generation sequencing	Broad Institute (US)
Blood	Next-generation sequencing	Personal Genome Diagnostics, Inc. (US)

9.1 Storage and disposal of samples

Samples collected for this additional study will be stored at LSI Corporation and the laboratories for gene testing shown in Section 9.0. Before DNA extraction, tumor samples will be stored at room temperature, while DNA samples extracted from tumor samples and blood samples will be stored in a deep freezer set at −80 °C.

The submitted tumor samples, blood samples, and tumor tissues, as well as DNA samples obtained from the blood samples, may be secondarily used for examinations of biomarkers to be revealed in the future. Consequently, in this additional study, “consent for secondary use of samples” will be verified with the patients, and samples from the patients who provided consent will be stored for a maximum of 20 years after the end of the additional study for the purpose of using the samples for future medical research other than the examination of biomarkers specified in the additional study. These samples will be properly discarded as medical waste after deleting the patient numbers at the time when it is determined that they will not be used or the storage period is terminated, and records on disposal will be kept. In addition to this, the samples will be discarded if the patients who provided the samples withdraw their consent, the patient numbers cannot be recognized (as a result of issues with labels and computers), the samples become mislabeled, or disposal is found by the researchers to be needed for other reasons. At each site where samples that should be discarded because of reasons such as withdrawal of consent are stored, the investigators at that site should discard the samples. When stored samples are secondarily used for medical research in the future, a protocol should be prepared again, and approval of the IRB should be obtained as necessary.

10.0 COMMITTEES ESTABLISHED FOR THE ADDITIONAL STUDY

10.1 Study Steering Committee

The Study Steering Committee will be established for effectively promoting the additional study.

The Study Steering Committee will be composed of the sponsor, study representatives, a pathologist, and Study Steering Committee members. The sponsor, or a person designated by the sponsor, will serve as the secretariat. Throughout the study period, the Study Steering Committee will not be notified about the details of treatment randomization.

The details of the Study Steering Committee are specified in a separately issued procedure.

For the Clinical Study Steering Committee members, see Section 1.2.

10.2 Pathology specialist

The pathology specialist will provide advice on the handling of the tumor samples and blood samples and will provide expertise in interpreting results in this additional study. The details of the role of the pathologist are specified in a separately issued written procedure.

For the pathology specialist, see Section 1.2.

10.3 Clinical genetics specialist

The clinical genetics specialist will provide advice on analysis results from the additional study and will help assess the obtained results. Detailed roles of the clinical genetics specialist are specified in a separately issued written procedure.

For the clinical genetics specialist, see Section 1.2.

11.0 RETENTION OF RECORDS

11.1 Case report form

A case report form will not be prepared for this additional study. The date of acquisition of the consent form for this additional study should be included in the case report form of the main study.

11.2 Storage of records

The investigator or the head of each study site should store the following materials, including those specified in Section 13.1, and study-specific documents for use by the investigation or audit by regulatory authorities and the sponsor or their designee. The materials include a list of patient screening, a patient identification code list, medical records, signed and dated original consent forms, etc. The investigator and the head of study site should also store the essential documents for up to 5 years after the date of the report about final completion of the study or for 3 years after the date of the report about the final announcement of study results, whichever comes later. However, when the sponsor requires a longer storage period, the head of study site will discuss the period and methods of storage with the sponsor.

The investigator and the head of study site will store the essential documents until the sponsor notifies that storage is no longer necessary.

12.0 STATISTICAL PROCEDURES

12.1 Statistical analysis plan

Prior to the start of the statistical analyses, an analyst should prepare a statistical analysis plan (first version). The statistical analysis plan should describe the definitions of endpoints and details of analysis methods for handling all the objectives of the study.

12.1.1 Analysis population

As an analysis population in this additional study, a "full analysis set" (FAS) will be established. The FAS to be used as the primary analysis population for the analysis of the primary endpoint will be defined as "patients in the FAS in the main study from whom measured values of the biomarkers are obtained." The detailed definition of the analysis population is specified separately in the statistical analysis plan.

The analyst should make a final decision on the appropriateness of the definition of the analysis population and rules for handling of case data from the population in analyses, upon discussion with a statistical analysis manager, before data fixation.

12.1.2 Analyses of demographic and other baseline characteristics

Demographic and other baseline characteristics will be descriptively summarized by treatment group.

12.1.3 Analysis of the primary endpoint

The primary endpoint is evaluation of the relationship between OS and mutation of each gene (eg, *BRAF*, *PIK3CA*, *EGFR*, *KRAS*, and *NRAS*) in tumor tissue.

The following analysis will be performed with the FAS: by presence or absence of each gene mutation in tumor tissue, Kaplan-Meier survival curves of time to event will be shown by treatment group. A quartile point of survival by treatment group, and point estimates with two-sided 95% confidence intervals for survival rates at certain time points, will be calculated.

12.2 Interim analysis and criteria for premature termination

No interim analysis will be implemented.

12.3 Determination of the planned sample size

This is an additional study of the main study, and thus the planned sample size will be within the number of patients who are enrolled in the main study. Because this is an exploratory study, there is no rationale for determination.

13.0 QUALITY CONTROL AND QUALITY ASSURANCE

13.1 Monitoring of the study sites

The sponsor or a person designated by the sponsor should periodically perform monitoring at the study sites throughout the study period to verify that the study is conducted in accordance with each detail described in the study protocol. In the additional study, central monitoring, and, as necessary, site visit monitoring, will be implemented. In the site visit monitoring, data recorded in the case report form will be cross-checked with source documents to confirm that the data are accurate. The source documents are the originals of documents, data, and records. The investigators and the head of the study site should ensure that the sponsor, or the person designated by the sponsor and committees at the study site such as the IRB, has access to the source documents.

The sponsor or the person designated by the sponsor should review records, including a list of the patient codes, medical charts, and the original signed and dated informed consent forms, to verify that the study is properly conducted in compliance with the study protocol. The investigators and other persons involved in the study should allow sufficient time for undertaking monitoring operations and make an effort to cooperate when a visit at the study site is made for monitoring.

A detailed procedure about monitoring will be stated in the quality monitoring plan.

13.1.1 Central monitoring

Central monitoring will be carried out to check whether the study is implemented safely and in accordance with the protocol, according to data reported through the case report form, and that data are accurately collected. In principle, the central monitoring should be performed twice per year, and a periodic monitoring report should be prepared. The periodic monitoring report will be evaluated by the Study Steering Committee, and feedback will be given to the study site as necessary.

Details of the central monitoring are described in a separately issued procedure.

13.1.2 Site visit monitoring

Site visit monitoring is conducted with the following objectives: to check the data of the additional study recorded in the case report form in the main study against source documents to confirm that the study has been conducted safely and in compliance with the protocol, and also to check that data have been collected accurately.

In site visit monitoring, source data verification will be performed on some of the registered patients.

The frequency and procedure of site visit monitoring will follow the monitoring plan defined separately.

13.2 Deviation from Ethical Guidelines on Medical Research Involving Human Subjects and study protocol

The investigator or researchers make record of all deviations from Ethical Guidelines on Medical Research Involving Human Subjects, ICH-GCP, and study protocol. The investigator or researchers must promptly notify the head of the study site and the study sponsor, in writing, when a deviation is identified. As required, the investigator or researchers must consult with the sponsor about revision of

study protocol. When the protocol is revised, it is submitted to the head of the study site to obtain approval from the committee of the study site, such as the IRB.

13.3 Quality assurance and investigation by the regulatory authority

The sponsor or a person designated by the sponsor should, as necessary, perform audits at the study site. In such a case, auditors appointed by the sponsor should contact the study site beforehand and schedule a visit for an audit. The auditors may request to visit a place where samples for laboratory tests are collected and other places used during the study period. The investigator and the head of the study site should ensure that the auditors have access to all the source documents mentioned in Section 13.1.

14.0 ETHICAL CONDUCT OF THE STUDY

This additional study will be conducted in compliance with the protocol and ethical principles based on the Declaration of Helsinki, Ethical Guidelines on Medical Research Involving Human Subjects, and ICH-GCP to preserve the interests of study participants. Each investigator should conduct the study based on regulatory requirements and in compliance with the responsibilities of the investigator described in Appendix A.

14.1 Conflict of interest

This study is conducted under the auspices of the sponsor.

Prior to the study, the investigator should attest that they have no COI with regard to this study, according to the stipulation of the site.[23-27]

The study site should comply with all the requirements specified by a committee such as the IRB. The requirements include the COI self-declaration, protocol, and informed consent form.

14.2 Approval of committees at the study site such as the IRB

Committees at the study site, such as the IRB, will be composed in accordance with regulations in regions participating in the additional study. The sponsor, or a person designated by the sponsor, should obtain a document listing the name and job title of each committee member.

The sponsor, or the person designated by the sponsor, should provide committees at the study site, such as the IRB, with related documents for review and approval of the protocol. Also, in addition to the protocol for the additional study, copies of the informed consent document and, as necessary, materials on patient recruitment and advertisement and other documents required by regulations, shall be submitted to the central committee or committees at the study site, such as the IRB, to request approval.

The sponsor should notify the study site and the investigators and researchers after confirming the appropriateness of the regulatory documents of the study site. Protocol procedures, such as obtaining consent, should not be started until the study site, the investigator, and researchers receive the notification. The study site shall comply with all requirements stipulated by the committees at the study site such as the IRB. These include notification to the committees at the study site, such as the IRB, of the submission of a protocol revision, revision of the informed consent document, revision of materials for patient recruitment and safety in accordance with regulatory requirements, report of the implementation status of the study made at intervals set by the committees at the study site such as the IRB, and report on the study completion. The sponsor or the person designated by the sponsor should obtain the approval document of the committees at the study site, such as the IRB, for the above-mentioned matters and for all of the related documents.

14.3 Informed consent document and consent of patients

The informed consent form contains specific requirements of the Declaration of Helsinki, Ethical Guidelines on Medical Research Involving Human Subjects, ICH-GCP, and all applicable laws and regulations. The informed consent form specifies how patients' personal and medical information will be used in this additional study (both inside and outside of Japan) and provides information about disclosure of that information to a third party. The informed consent form also explains the general idea and purpose of the study and its possible risks and benefits, clarifies the conditions for study participation, and states the fact that patients can discontinue study participation at any time without giving reasons

and without loss of benefits in treatment. In principle, the following items are explained in the informed consent form:

1. About the informed consent
2. Title of the study and the fact that the study has been approved by the head of the study site
3. Name of the Study Steering Committee Chairman, name of the study site, and name of the investigator
4. Objectives and significance of the study
5. Study method (including purpose of use of the samples and information obtained from patients) and duration
6. Planned number of patients who will participate
7. Reason for being selected as a participant
8. Burden on patients and anticipated risks and benefits
9. Consent for participation
10. The fact that patients can withdraw from participation at any time, even if they agreed that the study would be conducted or extended
11. The fact that patients will not suffer any disadvantages, even if they do not consent to participate in the study or extend their participation in the study or if they withdraw from the study
12. In what ways information from the study will be disclosed
13. The fact that, when requested from patients, the study protocol and materials regarding the study method will be available, within a range that would not interfere with protection of personal data of other patients and originality of the study, and how to obtain and inspect them
14. Handling of personal information, etc (including anonymization method)
15. Storage and disposal methods of samples and information
16. Status of funding source for the study, etc; COI for the study site involved in the study and personal profit, etc; COI for researchers, etc, engaged in the study
17. Intellectual property
18. Management of consultation with patients and their caregivers
19. Burden of expense
20. Whether or not compensation for health hazard caused by the study is provided and its content
21. Possibility of using samples and data for future studies
22. The fact that persons engaged in monitoring and inspection, and IRB members, are able to inspect samples and information of the study participants within a range as required given that privacy of patients will be protected
23. The fact that consent cannot be obtained from others besides the patient himself/herself

The investigator is responsible for preparation, content, and IRB approval of the informed consent form. The informed consent form should be approved by the IRB before use.

The informed consent form should be written in language easily understood by patients. The investigator or researchers are responsible for providing detailed explanation of the informed consent form to patients. Information should be provided orally and in writing as best as possible by the method deemed appropriate by the IRB.

The investigator or researchers should ensure that the patients have (1) an opportunity to inquire about the study and (2) sufficient time to come to a decision about study participation. When a patient decides to participate in the study, the patient should sign or write name / affix seal and date the consent form prior to study participation. The investigator or researchers should request that the patient sign or write name / affix seal with black or blue ballpoint pen using a legal name and not a popular name. The investigator or researchers should also sign or write name / affix seal and date the consent form prior to patient participation. In addition, when the study collaborator gives a supplemental explanation, the study collaborator concerned should also sign or write name / affix seal and date the consent form.

The investigator or researchers should store the original consent form that was signed or contains the name / affixed seal. The investigator or researchers should document in the patient's medical record the date when the patient signed or wrote name / affixed seal on the consent form. A copy of the consent form, with signature or name typed with name seal affixed, should be provided to the participant.

The investigator or researchers should take the same procedures as those for obtaining the initial consent to newly obtain consent from the concerned patient when the informed consent form is revised. The date of obtaining new consent should be recorded in the patient's medical record, and a copy of the revised consent form should be provided to the patient.

14.4 Patient confidentiality

The sponsor and persons designated by the sponsor should comply with the principles of the protection of the patients' right against invasion of privacy. In the additional study, the sponsor's clinical study database and study-related documents should be related to raw data by means of patient ID codes. Limited information on the patient, such as gender, age, and birthday, may be used within the scope of all applicable laws and regulations for identifying the patients and verifying the accuracy of the patient ID codes.

In order to confirm that the study is conducted in compliance with the protocol for the additional study, the sponsor should request that the investigator permit monitors or persons designated by the sponsor, representatives of the regulatory authorities, auditors appointed by the sponsor, and the IRB to have access to laboratory data, electrocardiograms, hospitalization records during the period of study participation, and original medical records (raw data or documents) such as autopsy results. When receiving informed consent from the patients, the investigators should obtain consent from the patients for access to the original medical charts by monitors, representatives of the regulatory authorities, and other relevant persons (see Section 14.3).

When providing copies of the source documents to the sponsor, the investigators should delete information enabling the identification of individuals (the name and address of the patient and other information identifying individual patients not recorded in the case report form).

14.5 Consultation services for patients and persons involved

The investigator provides a consultation service to respond to questions from patients and caregivers regarding the study. Details about the service will be stated in the consent form.

14.6 Advantages and disadvantages to participants

14.6.1 Advantages to participants

If the additional study enhances knowledge on biomarkers such as genes for predicting the efficacy of anti-EGFR antibody drugs such as panitumumab in patients with CRC, drug susceptibility and prognoses can be more properly predicted. It is expected that this progress will not only clinically contribute to the treatment of CRC but will also lead to the development of therapies suitable for individuals.

14.6.2 Disadvantages to participants

In the additional study, only tumor samples (surgical or endoscopic biopsy samples) and blood samples will be used. For the tumor samples, those collected before enrollment in the additional study or accidentally collected after the discontinuation of the additional study will be employed. The blood samples will be collected in accordance with blood sampling in routine medical examination. Therefore, physical risk and risk for disadvantage associated with sample collection are likely to be minimal.

In the gene testing, only genes related to tumor proliferation will be tested and other genes or the overall genetic structure will not be examined. Consequently, the Ethics Guidelines for Human Genome/Gene Analysis Research are not applicable, but personal information will be kept anonymous and carefully controlled in the light of the intent of the Guidelines. Hence, there may be no damage to the patients' human rights or privacy.

14.6.3 Disclosure of test results

Test results will be disclosed to patients when requested. However, when test results are disclosed to patients, it should be taken into consideration that this additional study is an exploratory study whose results should be proved by separate clinical study, and that accuracy and reliability of the laboratory procedure are immature as information to be disclosed to patients, and the presence of uncertainty cannot be denied.

14.7 Guidelines for publication, disclosure, and study registration

14.7.1 Report, publication, and disclosure of test results

The investigator should report the summary of test results in writing to the head of the study site and at the same time should provide the sponsor with all the results and data obtained from the study. Only the sponsor may disclose the study information to other investigators, researchers, or regulatory authorities during the study period, except for cases required by laws and regulations. The sponsor will be responsible for publication of the protocol and study-related results (including the public website), except for cases permitted in the study contract.

The sponsor may make public the data and information obtained from the study (including the data and information provided by the investigator) based on the agreement with the Study Steering Committee chairperson.

The investigator or researchers should obtain the prior written consent of the sponsor when making public the information obtained in this additional study at a specialized academic meeting, etc.

The investigator should report to the head of the study site when the test results of the study are published.

14.7.2 Clinical study registration

Takeda Pharmaceutical Company Limited will register all clinical studies involving patients conducted in the world to at least ClinicalTrials.gov and a website for disclosure (Japan Pharmaceutical Information Center [JAPIC]) prior to the start of the clinical studies to ensure that clinical study information is published in a timely manner and will comply with applicable laws, regulations, and guidelines. The status of patient recruitment in cities and countries where the studies are conducted, as well as the contact information of Takeda Pharmaceutical Company Limited, will be registered so that the information can be reviewed by the general public.

14.7.3 Disclosure of study results

Takeda Pharmaceutical Company Limited should, regardless of the results, present clinical study results on the ClinicalTrials.gov website and the website for disclosure (JAPIC), as stipulated by applicable laws and/or regulations.

14.8 Attribution of study results and intellectual property right

The results of the additional study will belong to Takeda Pharmaceutical Company Limited. Also, intellectual rights for drug products manufactured and distributed by Takeda Pharmaceutical Company Limited belong to Takeda Pharmaceutical Company Limited.

14.9 Insurance/compensation for damages

The patients participating in this study will be compensated for any injury resulting from participation in the study according to local regulations applicable to the study site. It should be noted that any treatment provided will be covered by health insurance, and no monetary compensation will be provided.

15.0 REFERENCES

1. Schwartzerg LS, et al. J Clin Oncol. 31(suppl):abstract 3631.
2. Heineman V, et al. ASCO 2013 (abstract LBA3506).
3. Lenz H, et al. Ann Oncol 2014;25(suppl 4):v1.
4. Douillard JY, et al. J Clin Oncol 2010;28(31):4697–705.
5. Peeters M, et al. Clin Cancer Res 2013;19(7):1902–12.
6. Douillard JY, et al. N Engl J Med 2013;369(11):1023–34.
7. De Roock W, et al. Lancet Oncol 2011;12(6):594–03.
8. Morelli P, et al. J Clin Oncol 2013;(suppl 15):abstract 3512.
9. Peng PJ, et al. Cancer Chemother Pharmacol 2013;72(2):323–8.
10. Esposito C, et al. Cancer Biol Ther 2013;14(12):1143–6.
11. Liao X, et al. N Engl J Med 2012;367:1596–606.
12. Siena S, et al. J Clin Oncol 2010;28(suppl 15s):abstract 3566.
13. Peeters M, et al. J Clin Oncol 2010;28(suppl 15s):abstract 3565.
14. Misale S, et al. Nature 2012;486:532–6.
15. Diaz LA Jr, et al. Nature 2012;486(7404):537–40.
16. Stein A, et al. Z Gastroenterol 2013;51:K62.
17. Mohan S, et al. PLoS Genet 2014;10(3):e1004271.
18. Peeters M, et al. Clin Cancer Res 2013;19:1902–12.
19. Di Nicolantonio F, et al. J Clin Oncol 2008;26:5705–12.
20. De Roock W, et al. Lancet Oncol. 2010;11(8):753–62.
21. Prenen H, et al. Clin Cancer Res 2009;15(9):3184–88.
22. Montagut C, et al. Nat Med 2012;18:221–3.
23. *Report of the Conflict of Interest Working Group*. Ministry of Education, Culture, Sports, Science and Technology. November 1, 2002.
24. *Guidelines for Establishment of Conflict of Interest Policies for Clinical Studies*. Study Group for Ethics and Conflict of Interest for Clinical Studies. March 2006.
25. *Guidelines for Management of Conflict of Interest (COI) in Scientific Researches*. Ministry of Health, Labour and Welfare. March 31, 2008.
26. Clinical Working Group, Committee for COI. *Guidelines for COI Management in Medical Research*. Japanese Association of Medical Sciences. February 2011.
27. *Common Guidelines for Conflict of Interest for Clinical Studies*. Japanese Society of Internal Medicine; Japan Society of Hepatology; Japanese Circulation Society; Japan Endocrine Society; Japan Diabetes Society; Japanese Respiratory Society; Japanese Society of Hematology; Japanese Society of Allergology; and Japanese Association for Infectious Diseases. August 2011.

APPENDIX A RESPONSIBILITIES OF THE INVESTIGATOR

1. To appropriately conduct the clinical study in compliance with the protocol, Ethical Guidelines on Medical Research Involving Human Subjects, ICH-GCP, and in consideration of the human rights, safety, and welfare of patients
2. When assigning a part of important duties related to this additional study to researchers or study collaborators, to prepare a list of assigned duties and persons, submit it in advance to the head of study site, and to obtain approval
3. To prepare the informed consent form and revise it as necessary
4. To check the contents of the study contract
5. To provide sufficient information on the protocol, drug, and duties of each person to researchers and study collaborators, and to provide them with guidance and supervision
6. To select patients who satisfy the protocol, give explanation using written information, and obtain consent in writing
7. To be responsible for all medical judgments related to the study
8. To respond to requests by the head of the study site, and at least once yearly to report the most up-to-date information on progress to the head of the study site
9. To request the COI committee of each study site to review and approve that there are no COI issues with this study
10. To ensure, together with the head of the study site, that sufficient medical care is provided to patients for all study-related clinically problematic adverse events throughout the period of each patient's study participation and thereafter
11. When a participant is treated at another medical institution or department, and after obtaining the patient's consent, to inform a physician at that medical institution or department, in writing, of the patient's study participation and study completion/discontinuation, and then to prepare the record
12. To discuss a revision of the protocol, etc, when proposed by the sponsor
13. To report the study completion in writing to the head of study site

Statistical Analysis Plan

DATA ANALYSIS ACTIVITIES FOR PARADIGM STUDY

Takeda Pharmaceutical Company Limited

Date of preparation: July 7, 2022
Contract analysis organization: Hitoshi Fujimiya
(Dynacom DMS Control No.: DMS-TKD-003),
Contract analysis manager at Dynacom Inc.

1. Revision history

Revision History	Version date	Revision point	Person in charge	Approval seal
Version 1	2022.01.24	Original version	Hitoshi Fujimiya	
Version 2	2022.06.	Configuration changes<br>• Mobilization step: 5. to 5.2, 6.5, 6.4.1–6.4.10 to 6.6.1–6.6.10 Added: 5.1, 6.4<br>Content change<br>• 2. Summary of Statistical Analysis<br>• 3. Revision of objectives and compliance matters<br>• 4. Correction<br>• 5.2 Data addition, name change<br>• 6.2 Processing added<br>• Addition of 6.5 Nos. 2–4<br>• 6.6 Changes in genes to be analyzed<br>• 6.6.1 No. 9 and 6.6.2 No. 9 were added<br>• 6.6. Corrections of the contents of 6.6.1–6.6.10 Analysis methods<br>Terminology Correction<br>• Intervention—Treatment Group<br>• Tumor Location One Primary Tumor Location<br>• Clinical Information—Demographics		
Version 3	2022.07.01	Content change<br>• 6.6 Addition of genes to be analyzed and details of analysis methods<br>Correction		
Version 4	2022.07.07	Content change<br>• 6.6 Changes in target genes and analysis methods<br>Correction		

2. Overview of statistical analysis

<table>
<tr><td>Title</td><td colspan="3">DATA ANALYSIS ACTIVITIES FOR PARADIGM STUDY</td></tr>
<tr><td>Object</td><td colspan="3">A phase 3, randomized, comparative study to compare the efficacy and safety of mfolfox6 + bevacizumab combination therapy with mFOLFOX6 + panitumumab combination therapy in chemotherapy-naïve patients with unresectable advanced recurrent colorectal cancer who are wild type of RAS Gene (KRAS/NRAS gene) using demographic and genetic variant information of subjects, factors related to the efficacy of each therapy will be explored.</td></tr>
<tr><td>Sponsor</td><td colspan="3">Medical Affairs, Oncology Japan, Takeda Pharmaceutical Company Limited, Jumpe Shoeda</td></tr>
<tr><td>Contract analysis manager</td><td colspan="3">Dynacom Inc. Hiroshi Fujimiya</td></tr>
<tr><td>Analyst</td><td colspan="3">Dynacom Inc. Hitoshi Fujimiya, Chiaki Ito, Yuto Ogawa, Ikumi Mabuchi</td></tr>
<tr><td>Product Security Pharmacist</td><td colspan="3">Dynacom Inc. QC Group, Kiyomi Ouchi</td></tr>
<tr><td>Analysis Data Study No.</td><td colspan="3">PARADIGM biomarker study (NCT02394834)</td></tr>
<tr><td>SOP number for data transfer</td><td colspan="3">Specify separately</td></tr>
<tr><td>Number of data</td><td colspan="3">Colorectal cancer: Approximately 800 events</td></tr>
<tr><td rowspan="7">Analysis flow</td><td>Item</td><td>Overview</td><td>Remarks</td></tr>
<tr><td>Target data</td><td>• Objective variables: including overall survival (OS)
• Explanation variables: PGDx gene variant information, other clinical information, etc.</td><td>See Section 5 for details.</td></tr>
<tr><td>Cleansing, etc.</td><td>Type of data (continuous, category 1, NA), presence or absence of obstacles, cleaning of genetic data</td><td></td></tr>
<tr><td>Data tabulation</td><td>Min, Mean, Median, Quartiles, Max, NA count</td><td></td></tr>
<tr><td>Analytical methods</td><td>Survival analysis, etc.</td><td>See Section 6 for details.</td></tr>
<tr><td>Report summary</td><td>• Report (Word or PDF file)
• Analysis log file (HTML file) including graphs, etc.
• Data after cleansing or variable transformation (Excel or CSV file)
• Numeric files of analysis results (Excel or CSV file)</td><td></td></tr>
<tr><td></td><td>End of Document</td><td></td></tr>
<tr><td>Compliance</td><td colspan="3">At this study, Takeda Pharmaceutical Company Limited and Dynacom Inc. shall conduct the Operation in compliance with the “Ethical Guidelines for Medical and Health Research Involving Human Subjects,” “Law for Ensuring the Quality, Efficacy, and Safety of Drugs and Medical Devices” “Ethical Principles set forth in the Declaration of Helsinki,” and other relevant notifications.
All of the following are referred to collectively as the “Ethical Guidelines, etc.” and “Act on the Protection of Personal Information (May 30, 2003 • Law No. 57 Item)” applicable to the Operation. If there is any discrepancy, the Ethical Guidelines, etc. shall be followed. Data migration services (DMS) for data, documents, and related materials related to all analyses in the Contract Analysis Business Management System to be stored appropriately.</td></tr>
</table>

3. Table of contents

1. Revision history....2
2. Overview of statistical analysis....3
3. Table of contents....4
4. Objectives....5
5. Data for statistical analysis....5
5.1 Analysis sets....5
5.2 Items and specifications of target data....5
6. Statistical analysis....7
6.1 Data cleansing....7
6.2 Gene variant data cleaning....8
6.3 Data tabulation before analysis....8
6.4 Analysis items and contents of subject background information....8
6.5 Analysis items and contents of gene variant information....8
6.6 Efficacy analysis items and contents....8
6.6.1 Overall survival (OS)....9
6.6.2 Progression-free survival (PFS)....10
6.6.3 Response rate (RR)....11
6.6.4 Duration of response (DOR)....12
6.6.5 Curative resection rate....12
6.6.6 Early tumor shrinkage rate....13
6.6.7 Maximum degree of tumor shrinkage (depth of response)....14
6.6.8 Disease control rate (DCR)....14
6.6.9 Time-to-treatment failure (TTF)....15
6.6.10 Survival from the start of second-line treatment....16
7. Precautions and analysis software to be used....17
8. Analysis implementation structure....17
8.1 Contact information for analysis data....17
8.2 Commissioned analysis system....17
8.2.1 Contract analysis manager....17
8.2.2 Person in charge of statistical analysis....17
8.2.3 Product security pharmacist....17

4. Objectives

A phase 3, randomized, comparative study to compare the efficacy and safety of mFOLFOX6 + bevacizumab combination therapy with mFOLFOX6 + panitumumab combination therapy in chemotherapy-naïve patients with unresectable advanced recurrent colorectal cancer with wild type of *RAS* gene (*KRAS*/*NRAS* gene) (PARADIGM study, hereafter called "this study"). The factors related to the efficacy of each therapy will be explored using demographic and genetic variant information of subjects.

5. Data for statistical analysis

5.1 Analysis sets

This population consists of all subjects included in the full analysis set (FAS) for this study for whom information on genetic variants is available.

5.2 Items and specifications of target data

(1) Efficacy Endpoints

Item name	Number of measurements	Specification	Remarks
**Overall survival (OS)**	Some	Number of days (continuous), censored (binary)	
**Progression-free survival (PFS)**	Some	Number of days (continuous), censored (binary)	
**Response rate (RR)**	Some	Presence or absence of response (binary value)	
**Duration of response (DOR)**	Some	Number of days (continuous), censored (binary)	
**Curative resection rate**	Some	Curative resection (binary)	
**Early tumor response rate**	Some	Presence or absence of early tumor shrinkage (binary)	
**Maximum degree of tumor shrinkage (depth of response)**	Some	≥ -100, continuous	Consider categorical variables as needed
**Disease control rate (DCR)**	Some	Presence or absence of disease control (binary value)	
**Time-to-treatment failure (TTF)**	Some	Number of days (continuous), censored (binary)	
**Survival from the start of second-line treatment**	Some	Number of days (continuous), censored (binary)	

(2) Demographics

Item name	Number of measurements	Specification	Remarks
**Age at enrollment (years)**	All subjects	Continuous	
**Primary tumor location (right/left/other)**	All subjects	Categorical	Left side: Descending colon, sigmoid colon, rectal sigmoid region, single or multiple occurrences in the rectum<br>Right side: Single or multiple occurrences in the cecum, ascending colon, and transverse colon<br>Others: When multiple primary lesions exist on the right and left sides
**Gender (male/female)**	All subjects	Categorical cannula	

**Age at enrollment (≤ 64 years/ ≥ 65 years)**	All subjects	Categorical	
**Information on primary organ (single/multiple)**	All subjects	Categorical	
**Site (cecum/ascending colon/ transverse colon/descending colon/ sigmoid colon/ rectosigmoid/rectum)**	All subjects	Categorical	Since multiple choices are allowed for this item, tabulate and analyze for each item.
**Number of organs with metastases (0/1/2 or more)**	All subjects	Categorical	
**Metastatic organs (liver/lung/abdomen) Membrane/lymph node/ bone/adrenal/skin/other)**	All subjects	Categorical	Since multiple choices are allowed for this item, tabulate and analyze for each item.
**Metastatic organ (liver only/other)**	All subjects	Categorical	
**Primary resection (absent/present)**	All subjects	Categorical	
**Metastasectomy (absent/present)**	All subjects	Categorical	
**Palliative colostomy (no/yes)**	All subjects	Categorical	
**Bypass operation (absent/present)**	All subjects	Categorical	
**Radiation therapy (radical irradiation) (absent/present)**	All subjects	Categorical	
**Preoperative/adjuvant chemotherapy (preoperative/postoperative/ preoperative/operative) (after/no)**	All subjects	Categorical	
***RAS* gene test**	All subjects		
**Site of collection (primary lesion/ liver/lung/lymph node/others)**	All subjects	Categorical	
**Specimen type (biopsy/surgical)**	All subjects	Categorical	
**Histological type (papillary/tubular adenocarcinoma [well-differentiated/ moderately differentiated]/poorly differentiated adenocarcinoma [solid/non-solid]) Mucinous/signet-ring cell/ adenocarcinoma (NOS)/others**	All subjects	Categorical	
**Test method (RASKET/others)**	All subjects	Categorical	
***KRAS* results**	All subjects		
**EXON 2 codon 12 (wild type/mutant/ not measured or not evaluable)**	All subjects	Categorical cannula	
**EXON 2 codon 13 (wild type/mutant/ not measured or not evaluable)**	All subjects	Categorical cannula	
**EXON 3 codon 59 (wild type/mutant/ not measured or not evaluable)**	All subjects	Categorical	
**EXON 3 codon 61 (wild type/mutant/ not measured or not evaluable)**	All subjects	Categorical	
**EXON 4 codon 117 (wild type/mutant/ not measured or not evaluable)**	All subjects	Categorical cannula	

**EXON 4 codon 146 (wild type/ mutant/not measured or not evaluable)**	All subjects	Categorical	
***NRAS* Results**	All subjects		
**EXON 2 codon 12 (wild type/mutant/ not measured or not evaluable)**	All subjects	Categorical cannula	
**EXON 2 codon 13 (wild type/mutant/ not measured or not evaluable)**	All subjects	Categorical cannula	
**EXON 3 codon 59 (wild type/mutant/ not measured or not evaluable)**	All subjects	Categorical cannula	
**EXON 3 codon 61 (wild type/mutant/ not measured or not evaluable)**	All subjects	Categorical cannula	
**EXON 4 codon 117 (wild type/ mutant/not measured or not evaluable)**	All subjects	Categorical	
**EXON 4 codon 146 (wild type/ mutant/not measured or not evaluable)**	All subjects	Categorical	
**Past history (absent/present)**	All subjects	Categorical cannula	
**Comorbidity (absent/present)**	All subjects	Categorical cannula	
**ECOG PS at enrollment (0/1/2/3/4)**	All subjects	Categorical cannula	
**Follow-up period (years)**	All subjects	Continuous	

Abbreviation: ECOG PS: Eastern Cooperative Oncology Group performance status

(3) Gene Variant Information

Item name	Number of measurements	Specification	Remarks
**PGDx genetic variant information**	Some	Mutation information and tumor fraction for each gene in ctDNA collected before and after treatment	Process and use as aggregated value or presence/absence information
**BROAD gene variant information**	Some	Mutation information of each gene in tumor tissues collected before and after treatment	Process and use as aggregated value or presence/absence information
**TCGA colorectal cancer gene variant information**	(External data)	Mutation information for each gene	Process and use as aggregated value or presence/absence information

6. Statistical analysis

The following priority and analysis flow will be followed. The results will be shared as appropriate, and policies will be modified/changed as necessary.

6.1 Data cleansing

(1) Check the data type (continuous, category, NA) and presence or absence of obstacles.
(2) Removal or appropriate replacement of non-numerical information
(3) Presence or absence of missing values, examination of imputation

6.2 Gene variant data cleaning

Filter out the Japan-specific SNPs (ToMMo 8.3KJPN) for PGDx gene variant information. In addition, BROAD variant information should be extracted according to the following criteria: SNV: VAF >10%, coverage >75, Indel: VAF >20%, coverage >75.

6.3 Data tabulation before analysis

(1) Consideration of handling of outliers (return to cleansing as necessary)
(2) Analysis data version assignment (If a correction occurs after proceeding to 6.3 or later, the history is kept while upgrading.)
(3) Tabulation of gene variant information and various clinical data

6.4 Analysis items and contents of subject background information

No.	Analytical methods	Data	Process description	Result
**1**	Descriptive statistics calculation	Demographics	Demographic variables will be summarized using descriptive statistics. For continuous variables, the minimum, median, maximum, 14 quartiles, 34 quartiles, quartile range, mean, and standard deviation will be calculated.<br>For categorical variables, frequencies and percentages will be calculated, where these analyses will be performed by primary tumor location, overall, and on the left and right sides, respectively, by treatment group.	Descriptive statistics

6.5 Analysis items and contents of gene variant information

The following analyses may also be performed on the whole gene set and by pathway. In addition, it is also possible to perform the analysis with the combined data of tumor tissue and ctDNA before treatment or the information of each mutation before and after the start of treatment.

No.	Analytical methods	Data	Process description	Result
1	Diagram of Oncomap	Gene variant information and efficacy endpoints various, treatment group, and site of primary lesion	Construct Oncomap with pretreatment gene variant information. The heatmap of each efficacy endpoint and the number of mutations will be displayed together. The samples will be sorted by treatment group or primary tumor location to allow distribution in each group.	Oncomap
2	Fisher's exact test	Gene variant information, treatment group, and primary lesion location	The proportion of pretreatment gene variants will be compared by Fisher's exact test after dividing the groups by treatment group, primary lesion location, etc. In addition, the proportion of variants increased or decreased before and after treatment will be compared between the treatment groups for each gene.	Fisher's exact *P* value
3	Wilcoxon test	Gene variant information and treatment groups	The proportion of clonal mutations before and after treatment will be tabulated for each patient. Differences between treatment groups before and after treatment will be compared using the Wilcoxon rank sum test.	Wilcoxon test *P* value
4	Goodness-of-fit test	Gene variant information	The total number of mutations will be calculated for each gene, and the goodness-of-fit test will be performed using the total number of mutations in TCGA as the expected value.	*P* value for goodness-of-fit test

6.6 Efficacy analysis items and contents

Each analysis shown below will be performed using the data of each gene variant shown in (1) to (13) below.
(1) *RAS*
(2) *BRAF* (V600E)
(3) *BRAF* (non-V600E)
(4) *HER2* (amplification)
(5) *MET* (amplification)
(6) *EGFR* (extracellular domain)
(7) *PTEN*
(8) *ALK*/*RET*/*NTRK1* (fusion)

(9) *PIK3CA*
(10) *MSI*
(11) *TMB* (10, 25, 50, and 100 mutations/Mb)
(12) *RAS*, *BRAF* V600 (Class I), *HER2* (amplification), *MET* (amplification), *EGFR* (extracellular) domain), *PTEN*, and *ALK*/*RET*/*NTRK1* (fusion)
(13) Presence or absence of *RAS* and *BRAF* V600E (Class I) mutations, as well as *BRAF* (Class II or II&III), HER2 (amplification), *MET* (amplification), *EGFR* (extracellular domain), *PTEN*; presence or absence of mutations in either *ALK*/*RET*/*NTRK1* (fusion) or *PIK3CA* genes
(14) Presence or absence of mutations in all factors and presence or absence of mutations in any factor by any combination of factors shown in (1)–(10) even if it is other than (13)

As for the 91 genes for which PGDx gene variant information is to be measured, univariate analysis and multivariate analysis, including interaction terms based on multiple genes in each pathway {*RTK-RAS* (*AKT1*, *ARID1A*, *ARID2*, *ATM*, *AXL*, *BRAF*, *EGFR*, *ERBB2*, *ERBB3*, *ERBB4*, *FGFR1*, *FGFR2*, *FGFR3*, *GAS6*, *GNAS*, *HRAS*, *IGF2*, *IRS2*, *KRAS*, *MAP2K1*, *NRAS*, *PIK3CA*, *PIK3CB*, *PIK3R1*, *PTPN11*, *SRC*), *WNT* (*ARID1A*, *AMER1*, *APC*, *AXIN2*, *CCND1*, *CDH1*, *CTNNB1*, *HNF1A*, *MYC*, *SMO*, *SOX9*, *TCF7L2*, *TP53*), *TGF-b* activation (*MYC*, *ACVR2A*, *SMAD2*, *SMAD3*, *SMAD4*, *TGFBR1*, *ARID1A*), *DDR* (*PIK3CA*, *TP53*, *MLH1*, *MSH3*, *MSH6 POLE*), *CIMP* (*ARID2*, *PIK3CB*, *TET2*), apoptosis (*PIK3R1*. *PTPN11*, *MCL1*), and *JAK-STAT* (*ARID1A*, *MYC*, *CCND1*, *IL6*, *JAK2*, *JAK3*, *STAT3*) or other routes, are allowed.

For the analysis of the candidate biomarkers shown in (1)–(12), in which OS is evaluated as efficacy, *P* value (two-sided significance level: 0.05) and Q-value (threshold: 0.1) will be calculated to determine the significance. Analyses using other indexes as efficacy and evaluation of biomarker candidates shown in (13), (14), and "Others " will be performed for exploratory purposes, and therefore the Q-value will be handled as a reference value and the *P* value will be used widely for examination.

6.6.1 Overall survival (OS)

No.	Analytical methods	Data	Process description	Result
1	Median calculation	OS, treatment group, primary tumor location, and gene variant information	The median survival time and 95% confidence interval (CI; log-log scale) will be calculated by dividing the groups by the presence or absence of gene variants, treatment group, primary lesion location, etc.	Median OS (95% CI)
2	Kaplan-Meier survival curve	OS, treatment group, primary information on location of nest and gene variant	Groups were divided by presence or absence of gene variants, treatment group, primary lesion location, etc.<br>A Kaplan-Meier survival curve will be prepared.	Survival curve
3	Log-rank test	OS, treatment group, primary lesion location, and gene variant information	Survival time will be compared by log-rank test in subgroups stratified by presence or absence of gene variants, treatment group, primary lesion location, etc.	Log-rank *P* value
4	Annual survival rate calculation	OS, treatment group, primary lesion location, and gene variant information	The patients will be divided into groups according to the presence or absence of gene variants, treatment group, primary lesion location, etc., and the annual survival rate and 95% CI will be calculated.	Annual survival rate (95% CI)
5	Cox regression analysis	OS, information on gene variants, and information on subject demographics	A Cox regression model will be constructed with the presence or absence of gene variants, tumor fraction, subject background information, etc. as explanatory variables. In addition, the hazard ratio and its 95% CI will be calculated.	Hazard ratio (95% CI)

**6**	Multivariate Cox regression analysis	OS, information on gene variants, and information on subject demographics	Candidate biomarker factors will be searched by constructing a multivariate model while selecting variables using machine learning methods such as Cox-Lasso and Elastic Net using gene variant information, tumor fraction, and subject background information as explanatory variables.	
**7**	Model assessment	OS, information on gene variants, and information on subject demographics	For the models constructed in the above rows 5 and 6, survival curves will be drawn separately for high-risk/low-risk subjects. The regression coefficient and C-index will be calculated to evaluate the model. The model predictions will also be used to estimate which treatment group is more suitable.	Survival curve, regression coefficient, and C-index
**8**	Swimmer plot	OS	The survival time of each case is visualized by a swimmer plot.	Swimmer plot
**9**	Stratified log-rank test	OS, treatment group, gene variant information	Patients will be stratified by age and the presence or absence of liver metastasis, divided into groups by the presence or absence of genetic variants, treatment group, etc., and survival time will be compared by stratified log-rank test.	Stratified log-rank test *P* value

6.6.2 Progression-free survival (PFS)

No.	Analytical methods	Data	Process description	Result
**1**	Median calculation	PFS, treatment group, site of primary lesion, and gene variant information	The median survival time and 95% CI (log-log scale) will be calculated by dividing the groups by the presence or absence of gene variants, treatment group, primary lesion location, etc.	Median PFS (95% CI)
**2**	Kaplan-Meier survival curve	PFS, treatment group, site of primary lesion, and gene variant information	Patients will be divided into groups according to the presence or absence of gene variants, treatment group, primary lesion location, etc., and Kaplan-Meier survival curves will be prepared.	Survival curve
**3**	Log-rank test	PFS, treatment group, site of primary lesion, and gene variant information	Survival time will be compared by log-rank test in subgroups stratified by presence or absence of gene variants, treatment group, primary lesion location, etc.	Log-rank *P* value
**4**	Annual survival rate calculation	PFS, treatment group, site of primary lesion, and gene variant information	The patients will be divided into groups according to the presence or absence of gene variants, treatment group, primary lesion location, etc., and the annual survival rate and 95% CI will be calculated.	Annual survival rate (95% CI)
**5**	Cox regression analysis	PFS, information on gene variants, and information on subject demographics	A Cox regression model will be constructed with the presence or absence of gene variants, tumor fraction, subject background information, etc. as explanatory variables. In addition, the hazard ratio and its 95% CI will be calculated.	Hazard ratio (95% CI)
**6**	Multivariate Cox regression analysis	PFS, information on gene variants, and information on subject demographics	Candidate biomarker factors will be searched by constructing a multivariate model while selecting variables using machine learning methods such as Cox-Lasso and elastic-Ne using the presence or absence of gene variants, tumor fraction, and subject background information as explanatory variables.	

**7**	Model assessment	PFS, information on gene variants, and information on subject demographics	For the models constructed in the above rows 5 and 6, survival curves will be drawn separately for high-risk/low-risk subjects. The regression coefficient and C-index will be calculated to evaluate the model. The model predictions will also be used to estimate which treatment group is more suitable.	Survival curve, regression coefficient, and C-index
**8**	Swimmer plot	PFS	The survival time of each case is visualized by a swimmer plot.	Swimmer plot
**9**	Stratified log-rank test	PFS, treatment group, gene variant information	Patients will be stratified by age and the presence or absence of liver metastasis, divided into groups by the presence or absence of genetic variants, treatment group, etc., and survival time will be compared by stratified log-rank test.	Stratified log-rank test *P* value

6.6.3 Response rate (RR)

No.	Analytical methods	Data	Process description	Result
**1**	RR calculation	Presence or absence of response, treatment group, primary lesion location, gene variant information	Patients will be divided into groups according to the presence or absence of gene variants, treatment group, primary lesion location, etc, and calculate the response rate and 95% confidence interval.	RR (95% CI)
**2**	Fisher's exact test	Presence or absence of response, treatment group, primary lesion location, and gene variant information	Patients will be divided into groups according to the presence or absence of gene variants, treatment group, primary lesion location, etc., and the proportions will be compared by Fisher's exact test.	Fisher's exact *P* value
**3**	Logistic regression analysis	Presence or absence of response, information on gene variants, and information on subject demographics	A logistic regression model will be constructed using the presence or absence of gene variants, tumor fraction, subject background information, etc. as explanatory variables. Odds ratio and its 95% CI will also be calculated.	Odds ratio (95% CI)
**4**	Multivariate logistic regression analysis	Presence or absence of response, information on gene variants, and information on subject demographics	Candidate biomarker factors will be searched by constructing a multivariate model while selecting variables using machine learning methods such as Logistic-Lasso and Elastic Net using the presence or absence of gene variants, tumor fraction, and subject background information as explanatory variables.	
**5**	Model assessment	Presence or absence of response, information on gene variants, and information on subject demographics	With the models constructed in the above rows 3 and 4, the response rate and 95% CI will be calculated separately for high-risk/low-risk subjects. The model will be evaluated by plotting receiver operating characteristic (ROC) curves and calculating regression coefficients and area under the ROC curve (AUC). The model predictions will also be used to estimate which treatment group is more suitable.	RR (95% CI), ROC curve, AUC

6.6.4 Duration of response (DOR)

No.	Analytical methods	Data	Process description	Result
1	Median calculation	DOR, treatment group, primary lesion location, and genetic variant information	The median survival time and 95% CI (log-log scale) will be calculated by dividing the groups by the presence or absence of gene variants, treatment group, primary lesion location, etc.	Median survival time (95% CI)
2	Kaplan-Meier survival curve	DOR, treatment group, primary lesion location, and genetic variant information	Patients will be divided into groups according to the presence or absence of gene variants, treatment group, primary lesion location, etc., and Kaplan-Meier survival curves will be prepared.	Survival curve
3	Log-rank test	DOR, treatment group, primary lesion site, and genetic variance information	Survival time will be compared by log-rank test in subgroups stratified by presence or absence of gene variants, treatment group, primary lesion location, etc.	Log-rank *P* value
4	Annual survival rate calculation	DOR, treatment group, primary lesion location, and genetic variant information	The patients will be divided into groups according to the presence or absence of gene variants, treatment group, primary lesion location, etc., and the annual survival rate and 95% CI will be calculated.	Annual survival rate (95% CI)
5	Cox regression analysis	DOR, genetic variant information, test Demographics	A Cox regression model will be constructed with the presence or absence of gene variants, tumor fraction, subject background information, etc. as explanatory variables. In addition, the hazard ratio and its 95% CI will be calculated.	Hazard ratio (95% CI)
6	Multivariate Cox regression analysis	DOR, genetic variant information, and subject background information	Candidate biomarker factors will be searched by constructing a multivariate model while selecting variables using machine learning methods such as Cox-Lasso and Elastic Net using the presence or absence of gene variants, tumor fraction, and subject background information as explanatory variables.	
7	Model assessment	DOR, genetic variant information, and subject background information	For the models constructed in the above rows 5 and 6, survival curves will be drawn separately for high-risk/low-risk subjects. The regression coefficient and C-index will be calculated to evaluate the model. The model predictions will also be used to estimate which treatment group is more suitable.	Survival curve, regression coefficient, and C-index
8	Swimmer plot	DOR	The survival time of each case is visualized by a swimmer plot.	Swimmer plot

6.6.5 Curative resection rate

No.	Analytical methods	Data	Process description	Result
1	Calculation of the proportion of curative resection	Presence or absence of curative resection, treatment group, site of primary lesion, and gene variant information	Patients will be divided into groups according to the presence or absence of gene variants, treatment group, primary lesion location, etc., and the curative resection rate and 95% CI will be calculated.	Curative resection rate (95% CI)
2	Fisher's exact test	Presence or absence of curative resection, treatment group, site of primary lesion, and gene variant information	Patients will be divided into groups according to the presence or absence of gene variants, treatment group, primary lesion location, etc., and the proportions will be compared by Fisher's exact test.	Fisher's exact *P* value
3	Logistic regression analysis	Presence or absence of curative resection, information on gene variants, and information on subject demographics	A logistic regression model will be constructed using the presence or absence of gene variants, tumor fraction, subject background information, etc. as explanatory variables. Odds ratio and its 95% CI will also be calculated.	Odds ratio (95% CI)

**4**	Multivariate logistic regression analysis	With curative resection<br>Number gene variant information, subject background information	Presence or absence of gene variants, tumor<br>Candidate biomarker factors will be explored by constructing a multivariate model while selecting variables using machine learning methods such as Logistic-Lasso and Elastic Net using fractions, subject background information, etc. as explanatory variables.	
**5**	Model assessment	Presence or absence of curative resection, information on gene variants, and information on subject demographics	For the models constructed in the above rows 3 and 4, the proportion of curative resection and 95% CI will be calculated separately for high-risk/low-risk subjects. The model will be evaluated by plotting ROC and calculating regression coefficients and AUC. The model predictions will also be used to estimate which treatment group is more suitable.	Curative resection rate (95% CI), ROC bending line, AUC

6.6.6 Early tumor shrinkage rate

No.	Analytical methods	Data	Process description	Result
**1**	Calculation of early tumor shrinkage rate	Presence or absence of early tumor shrinkage, treatment group, site of primary lesion, and gene variant information	Patients will be divided into groups according to the presence or absence of gene variants, treatment group, primary lesion location, etc., and the early tumor shrinkage rate and 95% CI will be calculated.	Early tumor shrinkage rate (95% CI)
**2**	Fisher's exact test	Presence or absence of early tumor shrinkage, treatment group, site of primary lesion, and gene variant information	Patients will be divided into groups according to the presence or absence of gene variants, treatment group, primary lesion location, etc., and the proportions will be compared by Fisher's exact test.	Fisher's exact P value
**3**	Logistic regression analysis	Presence or absence of early tumor shrinkage, information on gene variants, and information on subject background characteristics	A logistic regression model will be constructed using the presence or absence of gene variants, tumor fraction, and subject background information, etc. as explanatory variables. Odds ratio and its 95% CI will also be calculated.	Odds ratio (95% CI)
**4**	Multivariate logistic regression analysis	Presence or absence of early tumor shrinkage, information on gene variants, and information on subject background characteristics	Candidate biomarker factors will be explored by constructing a multivariate model while selecting variables using machine learning methods such as Logistic-Lasso and Elastic Net using the presence or absence of gene variants, tumor fraction, and subject background information as explanatory variables.	
**5**	Model assessment	Presence or absence of early tumor shrinkage, heredity<br>Child variant information, subject background information	Testing of high-risk and low-risk models constructed in the above rows 3 and 4<br>The early tumor shrinkage rate and its 95% CI will be provided. The model will be evaluated by plotting ROC curves and calculating regression coefficients and AUC. The model predictions will also be used to estimate which treatment group is more suitable.	Early tumor shrinkage rate (95% CI), ROC curve, AUC

6.6.7 Maximum degree of tumor shrinkage (depth of response)

No.	Analytical methods	Data	Process description	Result
1	Calculation of mean (median) depth of response	Maximum degree of tumor shrinkage, treatment group, primary lesion location, and gene variant information	The subjects will be divided into groups according to the presence or absence of gene variants, treatment group, primary lesion location, etc., and the mean (median) depth of response and 95% CI will be calculated.	Mean and median depth of response (95% CI)
2	Wilcoxon test	Maximum degree of tumor shrinkage, treatment group, site of primary lesion, gene variant information	The subjects will be divided into groups according to the presence or absence of gene variants, treatment group, primary lesion location, etc., and compared by Wilcoxon's rank sum test.	Wilcoxon test *P* value
3	Multivariate regression analysis	Maximum degree of tumor shrinkage, information on gene variants, and information on subject demographics	Candidate biomarker factors will be searched by constructing a multivariate model while selecting variables using machine learning methods such as Lasso and Elastic Net using the presence or absence of gene variants, tumor fraction, and subject background information as explanatory variables.	
4	Model assessment	Maximum degree of tumor shrinkage, information on genetic variants, and information on subject demographics	For the model constructed in the above row 3, draw a scatter plot and calculate the regression coefficient and determination coefficient to evaluate the model. The model predictions will also be used to estimate which treatment group is more suitable.	Regression coefficient, scatter plot, coefficient of determination
5	Waterfall plot	Maximum degree of tumor shrinkage	The maximum degree of tumor shrinkage in each case will be visualized by a waterfall plot. The mutation status of each gene will be added.	Waterfall plot

6.6.8 Disease control rate (DCR)

No.	Analytical methods	Data	Process description	Result
1	Calculation of DCR	Presence or absence of disease control, treatment group, primary lesion Information on location of nest and gene variant	Patients were divided into groups according to the presence or absence of gene variants, treatment group, primary lesion location, etc., and the DCR and 95% CI were calculated.<br>CIs will be calculated.	DCR (95% CI)
2	Fisher's exact test	Presence or absence of disease control, treatment group, primary lesion location, and gene variant information	Patients will be divided into groups according to the presence or absence of gene variants, treatment group, primary lesion location, etc., and the proportions will be compared by Fisher's exact test.	Fisher's exact *P* value
3	Logistic regression analysis	Presence or absence of disease control, information on gene variants, and information on subject background characteristics	A logistic regression model will be constructed using the presence or absence of gene variants, tumor fraction, subject background information, etc. as explanatory variables. Odds ratio and its 95% CI will also be calculated.	Odds ratio (95% CI)

**4**	Multivariate logistic regression analysis	Presence or absence of disease control, information on gene variants, and information on subject background characteristics	Candidate biomarker factors will be searched by constructing a multivariate model while selecting variables using machine learning methods such as Logistic-Lasso and Elastic Net using the presence or absence of gene variants, tumor fraction, subject background information, etc. as explanatory variables.	
**5**	Model assessment	Presence or absence of disease control, information on gene variants, and information on subject background characteristics	With the models constructed in the above rows 3 and 4, the DCR and 95% CI will be calculated by dividing the subjects into high risk/low risk. The model will be evaluated by plotting ROC curves and calculating regression coefficients and AUC. The model predictions will also be used to estimate which treatment group is more suitable.	DCR (95% CI), ROC curve, AUC

6.6.9 Time-to-treatment failure (TTF)

No.	Analytical methods	Data	Process description	Result
**1**	Median calculation	Time-to-treatment failure, treatment group, primary lesion location, and gene variant information	The median survival time and 95% CI (log-log scale) will be calculated by dividing the groups by the presence or absence of gene variants, treatment group, primary lesion location, etc.	Median survival time (95% CI)
**2**	Kaplan-Meier survival curve	Time-to-treatment failure, treatment group, primary lesion location, and gene variant information	Patients will be divided into groups according to the presence or absence of gene variants, treatment group, primary lesion location, etc., and Kaplan-Meier survival curves will be prepared.	Survival curve
**3**	Log-rank test	Time-to-treatment failure, treatment group, primary lesion location and gene variant information	Groups were divided by presence or absence of gene variants, treatment group, primary lesion location, etc.<br>Survival time will be compared using the log-rank test.	Log-rank *P* value
**4**	Annual survival rate calculation	Time-to-treatment failure, treatment group, primary lesion location, and gene variant information	The patients will be divided into groups according to the presence or absence of gene variants, treatment group, primary lesion location, etc., and the annual survival rate and 95% CI will be calculated.	Annual survival rate<br>(95% CI)
**5**	Cox regression analysis	Time-to-treatment failure, gene variant information, and demographics	A Cox regression model will be constructed with the presence or absence of gene variants, tumor fraction, subject background information, etc. as explanatory variables. In addition, the hazard ratio and its 95% CI will be calculated.	Hazard ratio (95% CI)
**6**	Multivariate Cox regression analysis	Time-to-treatment failure, gene variant information, and demographics	Candidate biomarker factors will be searched by constructing a multivariate model while selecting variables using machine learning methods such as Cox-Lasso and Elastic Net using the presence or absence of gene variants, tumor fraction, and subject background information as explanatory variables.	

**7**	Model assessment	Time-to-treatment failure, gene variant information, and demographics	For the models constructed in the above rows 5 and 6, survival curves will be drawn separately for high-risk/low-risk subjects. The regression coefficient and C-index will be calculated to evaluate the model. The model predictions will also be used to estimate which treatment group is more suitable.	Survival curve, regression coefficient, and C-index
**8**	Swimmer plot	Time-to-treatment failure	The survival time of each case is visualized by a swimmer plot.	Swimmer plot

6.6.10 Survival from the start of second-line treatment

No.	Analytical methods	Data	Process description	Result
**1**	Median calculation	Survival time from the start of second-line treatment, treatment group, primary lesion location, and gene variant information	The median survival time and 95% CI (log-log scale) will be calculated by dividing the groups by the presence or absence of gene variants, treatment group, primary lesion location, etc.	Median survival time (95% CI)
**2**	Kaplan-Meier survival curve	Survival time from the start of second-line treatment, treatment group, primary lesion location, and gene variant information	Patients will be divided into groups according to the presence or absence of gene variants, treatment group, primary lesion location, etc., and Kaplan-Meier survival curves will be prepared.	Survival curve
**3**	Log-rank test	Survival from the start of second-line treatment, treatment group, Primary lesion location and gene variant information	Patients will be divided into groups based on the presence or absence of gene variants, treatment groups, primary lesion location, etc., and survival time will be compared by log-rank test.	Log-rank *P* value
**4**	Annual survival rate calculation (annual survival rate)	Survival time from the start of second-line treatment, treatment group, primary lesion location, and gene variant information	The patients will be divided into groups according to the presence or absence of gene variants, treatment group, primary lesion location, etc., and the annual survival rate and 95% CI will be calculated.	Annual survival rate (95% CI)
**5**	Cox regression analysis	Survival time from the start of second-line therapy, information on gene variants, and information on subject background characteristics	A Cox regression model will be constructed with the presence or absence of gene variants, tumor fraction, subject background information, etc. as explanatory variables. In addition, the hazard ratio and its 95% CI will be calculated.	Hazard ratio (95% CI)
**6**	Multivariate Cox regression analysis	Survival time from the start of second-line therapy, information on gene variants, and information on subject background characteristics	Candidate biomarker factors will be searched by constructing a multivariate model while selecting variables using machine learning methods such as Cox-Lasso and Elastic Net using the presence or absence of gene variants, tumor fraction, and subject background information as explanatory variables.	

**7**	Model assessment	Survival time from the start of second-line therapy, information on gene variants, and information on subject background characteristics	For the models constructed in the above rows 5 and 6, survival curves will be drawn separately for high-risk/low-risk subjects. The regression coefficient and C-index will be calculated to evaluate the model. The model predictions will also be used to estimate which treatment group is more suitable.	Survival curve, regression coefficient, and C-index
**8**	Swimmer plot	Survival from the start of second-line treatment	The survival time of each case is visualized by a swimmer plot.	Swimmer plot

7. Precautions and analysis software to be used

(1) Should difficult mathematical problems arise in each statistical analysis, alternative approaches should be considered, and necessary changes should be made as appropriate.
(2) The analysis log file shall include the data version and script version used.
(3) Conduct a stakeholder review, and record the attendees and observations in the minutes.
(4) R version 4 will be used for the analysis processing.
(5) The library used should be output to the end of the analysis log file.

8. Analysis implementation structure

8.1 Contact information for analysis data

Takeda Pharmaceutical Company Limited
Japan Oncology Business Unit, Medical Affairs
Manager: Ikuo Mori DVM., PhD
Email: ikuo.mori@takeda.com
T103-8668 1-1 Nihonbashi Honcho 1-chome, Chuo-ku, Tokyo
TEL: 070-4286-1140

8.2 Commissioned analysis system

8.2.1 Contract analysis manager

Dynacom Inc.
Representative Director: Hitoshi Fujimiya PhD
Email: h.fujimiya@dynacom.co.jp
-6-1 Nakase 2, Mihama-ku, Chiba-city, Chiba 261-7125
WBG Mali East 25F
TEL: 043-213-8131
FAX: 043-213-8132

8.2.2 Person in charge of statistical analysis

Dynacom Inc.
Representative Director: Hitoshi Fujimiya PhD
Email: h.fujimiya@dynacom.co.jp
Chiaki Ito, Development Department
email: itoh@dynacom.co.jp
Ikumi Mabuchi, Development Department
Email: i-mabuchi@dynacom.co.jp
-6-1 Nakase 2, Mihama-ku, Chiba-city, Chiba 261-7125
WBG Mali East 25F
TEL: 043-213-8131
FAX: 043-213-8132

8.2.3 Product security pharmacist

Dynacom Inc.
Kiyomi Kouchi, Quality Check Department
Email: kouchi@dynacom.co.jp
-6-1 Nakase 2, Mihama-ku, Chiba-city, Chiba 261-7125
WBG Mali East 25F
TEL: 043-213-8131
FAX: 043-213-8132
