# Supplementary material for: Baseline ctDNA gene alterations as a biomarker of survival after panitumumab and chemotherapy in metastatic colorectal cancer
Source: Nat Med. 2024 Feb 12;30(3):730–9. doi: 10.1038/s41591-023-02791-w (PMC10957476; doi:10.1038/s41591-023-02791-w)
Supplement: Supplementary file 2 — Reporting Summary [file 41591_2023_2791_MOESM2_ESM.pdf]

nature portfolio

Corresponding author(s): Kohei Shitara

Last updated by author(s): Dec 14, 2023

Reporting Summary

Nature Portfolio wishes to improve the reproducibility of the work that we publish. This form provides structure for consistency and transparency in reporting. For further information on Nature Portfolio policies, see our Editorial Policies and the Editorial Policy Checklist.

Statistics

For all statistical analyses, confirm that the following items are present in the figure legend, table legend, main text, or Methods section.

n/a	Confirmed	
☐	☒	The exact sample size (*n*) for each experimental group/condition, given as a discrete number and unit of measurement
☐	☒	A statement on whether measurements were taken from distinct samples or whether the same sample was measured repeatedly
☐	☒	The statistical test(s) used AND whether they are one- or two-sided<br>*Only common tests should be described solely by name; describe more complex techniques in the Methods section.*
☐	☒	A description of all covariates tested
☒	☐	A description of any assumptions or corrections, such as tests of normality and adjustment for multiple comparisons
☐	☒	A full description of the statistical parameters including central tendency (e.g. means) or other basic estimates (e.g. regression coefficient) AND variation (e.g. standard deviation) or associated estimates of uncertainty (e.g. confidence intervals)
☐	☒	For null hypothesis testing, the test statistic (e.g. *F*, *t*, *r*) with confidence intervals, effect sizes, degrees of freedom and *P* value noted<br>*Give P values as exact values whenever suitable.*
☒	☐	For Bayesian analysis, information on the choice of priors and Markov chain Monte Carlo settings
☒	☐	For hierarchical and complex designs, identification of the appropriate level for tests and full reporting of outcomes
☒	☐	Estimates of effect sizes (e.g. Cohen's *d*, Pearson's *r*), indicating how they were calculated

Our web collection on statistics for biologists contains articles on many of the points above.

Software and code

Policy information about availability of computer code

Data collection	Clinical data were collected using the EDC Classic Rave (version 2020.2.0).
Data analysis	Statistical analyses were conducted using R (4.0.5), using following packages: gtsummary (1.7.0), survival (3.5-5), survminer (0.4.9), ggplot2 (3.4.2), forester (0.2.0), and Complex Heatmap (2.13.1).

For manuscripts utilizing custom algorithms or software that are central to the research but not yet described in published literature, software must be made available to editors and reviewers. We strongly encourage code deposition in a community repository (e.g. GitHub). See the Nature Portfolio guidelines for submitting code & software for further information.

Data

Policy information about availability of data

All manuscripts must include a data availability statement. This statement should provide the following information, where applicable:

- Accession codes, unique identifiers, or web links for publicly available datasets
- A description of any restrictions on data availability
- For clinical datasets or third party data, please ensure that the statement adheres to our policy

Data availability
The data sets, including individual participant data supporting the results reported in this article, will be made available within 3 months from initial request to researchers who provide a methodologically sound proposal. The initial contact for the request will be made with the corresponding author [Kohei Shitara]. The

data are not publicly available due to privacy/ethical restrictions and intellectual property reasons and will be provided after de-identification in compliance with applicable privacy laws, data protection, and requirements for consent and anonymization. Researchers will be requested to execute the contract with Takeda Pharmaceutical Company Ltd. for the usage of the data.

Human research participants

Policy information about studies involving human research participants and Sex and Gender in Research.

Reporting on sex and gender	A total of 254 patients of female sex and 479 patients of male sex were included in the analysis (see Table 1 in manuscript). No analysis was conducted based on gender.
Population characteristics	Of the 802 patients with RAS WT mCRC included in the PARADIGM efficacy analysis population, 733 patients (91.4%) provided informed consent for this biomarker study and had baseline blood plasma samples that were evaluable for ctDNA (Figure 1 in the manuscript). Among these 733 patients, 554 patients (75.6%) had left-sided primary tumors, 169 (23.1%) had right-sided primary tumors, and 10 (1.4%) had multiple primary lesions in both the left and right sides. A total of 432 patients (58.9%) were 65 to 79 years old, and 301 (41.1%) were 20 to 64 years old.
Recruitment	Participants were recruited by each investigator at the sites based on the study eligibility criteria. The recruitment process did not raise any concerns about selection bias.
Ethics oversight	The biomarker study protocol was approved by the institutional review boards or ethics committees at each participating center. This exploratory biomarker analysis included patients who were enrolled in the main study (PARADIGM) and provided informed consent for the additional biomarker study (NCT02394834). The protocol was reviewed and approved by the Certified Review Board of the National Cancer Center Hospital East, Japan.

Note that full information on the approval of the study protocol must also be provided in the manuscript.

Field-specific reporting

Please select the one below that is the best fit for your research. If you are not sure, read the appropriate sections before making your selection.

☒ Life sciences ☐ Behavioural & social sciences ☐ Ecological, evolutionary & environmental sciences

For a reference copy of the document with all sections, see nature.com/documents/nr-reporting-summary-flat.pdf

Life sciences study design

All studies must disclose on these points even when the disclosure is negative.

Sample size	This was an exploratory biomarker study of patients from a clinical trial. A total of 733 of the 802 patients in the primary study provided informed consent for this study, had baseline plasma samples evaluated for ctDNA, and were included in this biomarker study.
Data exclusions	No data were excluded from this exploratory study.
Replication	Because the study enrolled over 800 patients over a 2-year period at at 197 centers in Japan, and these patients were subsequently followed for survival for longer than 5 years, it would take at least 7 years to fully replicate the study. The time and cost involved in conducting a large clinical trial such as PARADIGM prohibit replication of the trial.
Randomization	In the primary study, patients were randomly allocated (1:1) to panitumumab plus mFOLFOX6 or to bevacizumab plus mFOLFOX6. Randomization was stratified by study site, age (20–64 vs 65–79 years), and presence or absence of liver metastases.
Blinding	The study was not blinded. The primary study, PARADIGM, was an open-label trial (i.e., unblinded), as predefined in the protocol. This exploratory biomarker study used samples from patients enrolled in the open-label study.

Reporting for specific materials, systems and methods

We require information from authors about some types of materials, experimental systems and methods used in many studies. Here, indicate whether each material, system or method listed is relevant to your study. If you are not sure if a list item applies to your research, read the appropriate section before selecting a response.

Materials & experimental systems

n/a	Involved in the study
☒	☐ Antibodies
☒	☐ Eukaryotic cell lines
☒	☐ Palaeontology and archaeology
☒	☐ Animals and other organisms
☐	☒ Clinical data
☒	☐ Dual use research of concern

Methods

n/a	Involved in the study
☒	☐ ChIP-seq
☒	☐ Flow cytometry
☒	☐ MRI-based neuroimaging

Clinical data

Policy information about clinical studies
All manuscripts should comply with the ICMJE guidelines for publication of clinical research and a completed CONSORT checklist must be included with all submissions.

Clinical trial registration

NCT02394834

Study protocol

This manuscript is reporting the results of an exploratory biomarker analysis. The protocol for the primary study (NCT02394795) was included as a supplementary item for the publication of the primary study manuscript (Watanabe J, et al. JAMA. 2023;329(15): 1271-1282.). The protocol and statistical analysis plan for this exploratory analysis are in included in the Supplementary Information for this manuscript.

Data collection

The data were entered into an electronic data capture system by the investigators and clinical research coordinators at each of the 197 clinical sites located throughout Japan. A listing of all investigators and their affiliations was provided in the supplementary materials of the primary publication for PARADIGM (Watanabe J, et al. JAMA. 2023;329(15):1271-1282). In this exploratory biomarker analysis, baseline plasma ctDNA (>10 ng/mL and >10 nM DNA) from enrolled patients was assessed using a custom next-generation sequencing (NGS)-based panel (PlasmaSELECT-R 91). The panel was designed to detect 90 mutations, 26 amplifications, and 3 rearrangements in mCRC-related genes, as well as microsatellite instability. Targeted genomic regions spanned 250 kb. Prespecified gene alterations for negative hyperselection for anti-EGFR antibody therapy were KRAS, NRAS, BRAF (V600E), PTEN, and extracellular domain EGFR mutations (exons 1–16 [1–620]), HER2 and MET amplifications, and ALK, RET, and NTRK1 fusions.

Outcomes

As prespecified in the Protocol, the primary endpoint of this exploratory study was to evaluate the relationship between overall survival and mutation of each gene (eg, BRAF, EGFR, KRAS, NRAS) in samples collected at baseline in the main study. Secondary endpoints were to evaluate the relationships between other efficacy endpoints (PFS, response rate, duration of response, proportion of patients who proceeded to surgical resection, proportion of patients with early tumor shrinkage, degree of maximum tumor shrinkage [depth of response]) of the main study and each tumor-associated gene in samples collected at baseline of the main study. The Protocol and Statistical Analysis Plan are available in the Supplementary Materials of this publication.
